# Optimal experimental design for efficient toxicity testing in microphysiological systems: A bone marrow application

**DOI:** 10.3389/fphar.2023.1142581

**Published:** 2023-03-31

**Authors:** Jonathan Cairns, Emilyanne Leonard, Kainat Khan, Conor Parks, Gareth Maglennon, Bairu Zhang, Stanley E. Lazic, Lorna Ewart, Rhiannon David

**Affiliations:** ^1^ Data Sciences and Quantitative Biology, Discovery Sciences, R&D, AstraZeneca, Cambridge, United Kingdom; ^2^ Integrated Bioanalysis, Clinical Pharmacology and Safety Sciences, R&D, AstraZeneca, Cambridge, United Kingdom; ^3^ Oncology Safety, Clinical Pharmacology and Safety Sciences, R&D, AstraZeneca, Cambridge, United Kingdom; ^4^ Pathology, Clinical Pharmacology and Safety Sciences, R&D, AstraZeneca, Cambridge, United Kingdom; ^5^ Safety Platforms, Clinical Pharmacology and Safety Sciences, R&D, AstraZeneca, Cambridge, United Kingdom; ^6^ Safety Innovation, Clinical Pharmacology and Safety Sciences, R&D, AstraZeneca, Cambridge, United Kingdom

**Keywords:** organ on a chip, experimental design, toxicity testing, bone marrow, flow cytometry

## Abstract

**Introduction:** Microphysiological systems (MPS; organ-on-a-chip) aim to recapitulate the 3D organ microenvironment and improve clinical predictivity relative to previous approaches. Though MPS studies provide great promise to explore treatment options in a multifactorial manner, they are often very complex. It is therefore important to assess and manage technical confounding factors, to maximise power, efficiency and scalability.

**Methods:** As an illustration of how MPS studies can benefit from a systematic evaluation of confounders, we developed an experimental design approach for a bone marrow (BM) MPS and tested it for a specified context of use, the assessment of lineage-specific toxicity.

**Results:** We demonstrated the accuracy of our multicolour flow cytometry set-up to determine cell type and maturity, and the viability of a “repeated measures” design where we sample from chips repeatedly for increased scalability and robustness. Importantly, we demonstrated an optimal way to arrange technical confounders. Accounting for these confounders in a mixed-model analysis pipeline increased power, which meant that the expected lineage-specific toxicities following treatment with olaparib or carboplatin were detected earlier and at lower doses. Furthermore, we performed a sample size analysis to estimate the appropriate number of replicates required for different effect sizes. This experimental design-based approach will generalise to other MPS set-ups.

**Discussion:** This design of experiments approach has established a groundwork for a reliable and reproducible *in vitro* analysis of BM toxicity in a MPS, and the lineage-specific toxicity data demonstrate the utility of this model for BM toxicity assessment. Toxicity data demonstrate the utility of this model for BM toxicity assessment.

## 1 Introduction

During the drug development process, accurate detection and prediction of safety endpoints pre-clinically is critical for progression to the clinic. Hence we require pre-clinical models that recapitulate adverse effects seen in humans and enable accurate prediction of clinical effects. Currently, pre-clinical safety assessment predominantly relies on two dimensional (2D) cell culture and *in vivo* animal models. However, translating results from these models to the clinic can be difficult, due to both the relative simplicity of 2D cell culture models and the differences between humans and animal models. Thus, there is a need for more physiologically relevant models. In recent years, complex cell culture models have emerged as a way to ‘bridge the gap’ between *in vitro* and *in vivo*. Three dimensional (3D) static models feature multiple cell types cultured in a hydrogel (collagen and/or fibronectin) or scaffolds (gelatin/hydroxyapatite), while Microphysiological Systems (MPS; organ-on-a-chip) build on this, creating a dynamic microenvironment through the addition of a fluidic component. Furthermore, mechanical stimulation (where appropriate) can be applied to recapitulate the structure and function of an organ more accurately (Bhatia and Ingber, 2014).

The improved relevance offered by 3D culture and MPS models, along with additional features including longer viable cell culture, has provided a great opportunity for application to the pharmaceutical pipeline. As it becomes increasingly practical, there is great scope for such experiments to scale up and span a greater experimental space–exploring a wide range of factors of interest, such as drug combinations, donors and dosing schedules. However, MPS studies are complex, with a number of potentially confounding factors that could introduce variability during the experiment, and could lead to irreproducible or non-robust conclusions if not accounted and controlled for. A statistically robust imaging setup for organ-chips was recently described which accounted for chip, row, and holder effects as sources of variability during the imaging process (Peel et al., 2019). However, since organ-chips are only recently being applied in research and development, there is not yet a standard study design that accounts for general MPS experimental factors. Various such factors group chips together, ranging from higher level (such as which incubator the chips are in, or who is operating them) to lower level (such as pump control units for microfluidic-based systems, holders for attaching accessory systems, or rockers to ensure that media stays in motion). Additionally, chips may be subdivided–either into separate channels, or into compartments separated by a permeable membrane (as is the case in liver chips, for example,).

**FIGURE 1:**
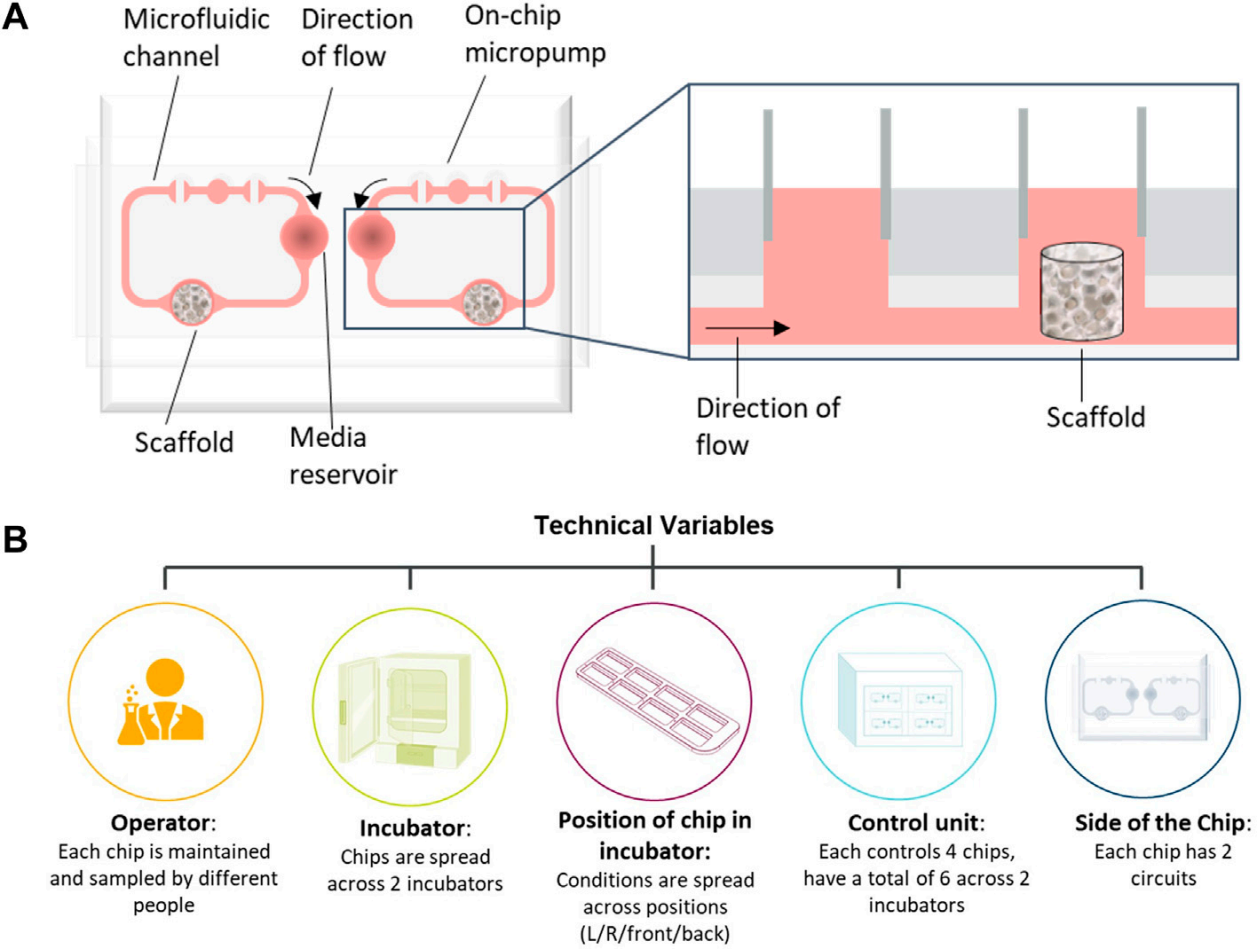
Bone marrow (BM) chip schematic and confounding factors. **(A)** Schematic of bone marrow microphysiological system (MPS) using the TissUse 2-Organ-Chip. **(B)** Schematic of possible confounding factors in the bone marrow MPS studies that have been accounted for in the study design.

To demonstrate how multifactorial MPS studies can be conducted in the face of many confounding factors, we describe the development and application of a statistical experimental design approach to a bone marrow MPS study. Bone marrow (BM) toxicity is an observed side effect of treatment with some oncology drugs that can be dose-limiting, requiring a reduction in the dose or necessitating dosing holidays, which can impact efficacy (Wang et al., 2006). It is vital therefore that pre-clinical safety models accurately predict BM toxicity. Currently, routine assessment of BM toxicity is conducted through two-dimensional (2D) cell culture and *in vivo* models, but both have their limitations; 2D culture only assesses progenitors rather than mature lineage-specific cells (Olaharski et al., 2009) and, while all blood cell lineages can be measured *in vivo*, BM assessment is a terminal endpoint and can only be measured at the end of the study, or requires large numbers of animals in each dose group to acquire BM toxicity data at multiple timepoints. Therefore, to predict human lineage-specific toxicity more accurately in the BM, there is a need for a more physiologically relevant model to mimic the heterogenous cell population. Our in-house human BM MPS is based on the model described by Sieber et al. (2018) using the TissUse two-organ microfluidic chip (Humimic Chip2) and a 3D ceramic scaffold (hydroxyapatite) with a pore size and structure that mimics the human cancellous bone (Figure 1A). CD34^+^ human stem and progenitor cells (HSPCs) are co-cultured onto a MSC-seeded scaffold, which provides the porous microenvironment. In addition, media is circulated *via* a microfluidic channel, which is crucial for maintaining the BM niche. This model has multiple potential confounding factors that need to be controlled to ensure the conclusions drawn from studies using this model are robust, including the incubator in which the chips are maintained, the control unit to which the chips are connected, and in the case of the Humimic Chip2, the side of the chip utilized for the scaffold (Figure 1B).

The aim of the current study was therefore to optimize the BM MPS experimental design by identifying and testing confounding factors. This approach was evaluated for a specific context of use: assessment of lineage-specific toxicity induced by two oncology drugs, olaparib and carboplatin. We demonstrate three key results as follows. First, we show that our multicolour flow cytometry set-up accurately labels the cells, both in terms of lineage and also their stage of maturation. This permits accurate quantification of treatment effect, down to the resolution of maturity level. Second, we show that it is viable to sample from chips repeatedly. This “repeated measures” design is dramatically more scalable and accounts for variability between chips, improving interpretation and permitting a far greater treatment space to be explored in one experiment. Third, we demonstrate an optimal way to arrange potential technical confounders, such as chip operator or control unit, in order to make our conclusions robust to them. We incorporate these factors into a mixed-model analysis pipeline. It increases the power relative to a naïve approach, thus maximising the value derived from studies that are expensive in terms of samples, time and money. Furthermore, our approach provides guidance on appropriate N for studies in practice.

Our results demonstrate an optimal experimental design approach to the design of MPS experiments that are generalisable to a variety of systems and scientific questions. By implementing a best practice approach for experimental design, we can maximise the reliability and reproducibility of safety predictions from MPS–not only in the context of the BM, but also with broader application, playing a role in driving the impact of organ chips in the pharmaceutical industry.

## 2 Materials and methods

### 2.1 BM MPS cell culture

Human Mesenchymal Stem Cells (MSCs; Lonza, Cambridge, United Kingdom) were cultured in Dulbecco’s Modified Eagle Medium (DMEM high glucose), supplemented with 5% (v/v) human platelet lysate (hPL, STEMCELL technologies, Cambridge, United Kingdom), 200 IU/mL penicillin, 200 μg/mL streptomycin and 1% (v/v) Glutamax and maintained for <5 passages post-thawing. A single MSC donor was used per experiment. MSCs (0.5 × 106 total cells) were seeded onto hydroxyapatite-coated zirconium oxide-based Sponceram^®^ 3D ceramic scaffolds (Zellwerk GmbH, Germany) in a 96 well ultra-low-attachment plate (ULA; Corning Biosciences, New York, United States) in media containing 10% foetal bovine serum (FBS). After 5–16 h, media and MSC-seeded ceramics were transferred to a 24 well ULA plate (Corning Biosciences) and cultured for 7 days with 100% media changes every 72–96 h.

After 1 week, ceramics were transferred to a 96 ULA plate, and Human BM CD34^+^ cells (HSPCs; Lonza) were seeded onto the MSC-seeded ceramics at a density of 1 × 104/200 μL per ceramic in StemSpanTM Serum-Free Expansion Medium (SFEM II; STEMCELL Technologies) supplemented with SCF (0.05 μg/mL), Flt-3-L (0.1 μg/mL), TPO (0.1 μg/mL; all from PeproTech, London, United Kingdom) (Chou et al., 2020). A single HPSC donor was used per experiment. HPSCs were incubated in the scaffold overnight following which the MSC-HSC- ceramic co-cultures were transferred into one compartment per circuit of the 2-Organ-Chips (TissUse GmbH, Berlin, Germany), while the other compartment served as a media reservoir. SFEM II medium (400 μL) with SCF (0.05 μg/mL), Flt-3-L (0.1 μg/mL), TPO (0.1 μg/mL), EPO (0.02 μg/mL) and G-CSF (0.001 μg/mL) (Chou et al., 2020), was added to each compartment of circuit (800 µL total volume per circuit) and the flow of recirculating media was directed through the medium reservoir first. Cultures were maintained for 7 days with a 50% media change after 72–96 h to allow differentiation of the HSPCs into cells of the different lineages. The donor information for the MSCs and HSPCs used in the studies are detailed in Table 1.

**TABLE 1:** Stem cell donors.

Experiment	Compound	Study design	MSC				HSC			
			Donor (Lot #)	Sex	Age	Race	Donor (Lot #)	Sex	Age	Race
1	Olaparib	Independent measures	0000423370	Female	23	Black	0000349536	Female	22	Black
2	Olaparib	Repeated measures								
3	Carboplatin	Repeated measures	0000451491	Male	25	Caucasian	0000680575	Female	21	Black

### 2.2 Cell treatment and collection

Following 7 days co-culture in the chip, scaffolds were treated with either olaparib or carboplatin for a defined period of time, followed by a period with no drug to allow for recovery of cells. The nominal concentrations, treatment schedules, and study design are detailed in Table 2. The concentrations of carboplatin and olaparib were selected to provide clinical translation based on AUC (van der Noll et al., 2020). Cells were collected for analysis at multiple timepoints throughout the study using one of two methods (i) independent measure where cells were sampled from the chips by flushing them from the scaffold. (ii) Repeated measure where cells which have moved out of the scaffold into the re-circulating media have been collected. This allows repeated sampling from a single circuit for the study duration. For the independent measures design, cells were collected from the scaffold by flushing with 400 μL phosphate-buffered saline with EDTA and 1% Bovine Serum Albumin (BSA) (PBE) before adding an additional 1.4 mL PBE and incubating at 37°C for 15 min. Cells were collected by centrifugation (400 × g, 5 min) and the pellet was resuspended in 1 mL PBE. For the repeated measures design, cells collected by removing the media were centrifuged (400 × g, 5 min) and resuspended in 1 mL PBE.

**TABLE 2:** Treatment schedule.

Experiment	Compound	Concentration (µM)	Vehicle	Vehicle concentration (%)	Treatment schedule	Sample points (days)	Study design
1	Olaparib	0, 1, 10	DMSO	0.01	14 days treatment	0, 14, 28	Independent measures
					14 days no drug		
2	Olaparib	0, 1, 10	DMSO	0.01	14 days treatment	0, 7, 14, 21, 28	Repeated measures
					14 days no drug		
3	Carboplatin	0, 2.5, 5, 10, 25, 50	Media (SFEM II)	N/A	1 day treatment	0, 1, 4, 7, 8, 11, 14	Repeated measures
					13 days no drug		

### 2.3 Flow cytometry panel selection

**FIGURE 2:**
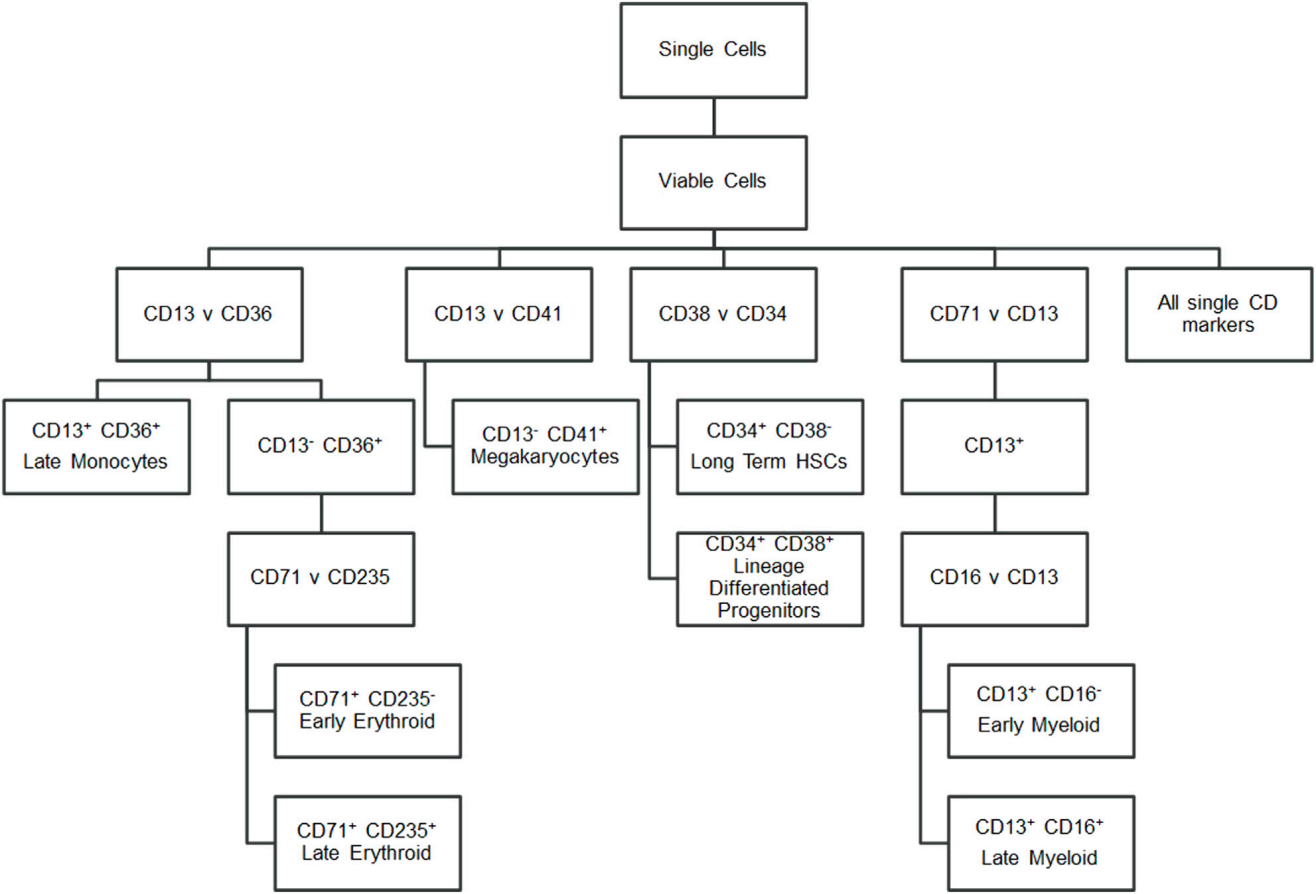
Flow cytometry gating strategy. Hierarchy showing the markers gated to identify cell populations.

To characterise the cells, a panel of CD markers was chosen to broadly indicate cell lineage and maturation level (Figure 2). CD34 and CD38 positivity determined long-term haematopoietic stem cells (LTHSCs) *versus* more differentiated haematopoietic stem cells (Lineage Differentiated Progenitors). CD41 was used to identify platelet lineage cells. CD13 was used to identify white cells, with additional CD36 positivity marking later monocytes and CD36 negativity identifying earlier myeloid cells. From the CD13 positive cells, a further comparison of these cells’ CD16 positivity gave an indication of their maturity, as CD16 is present on later cells in the neutrophil lineage (Wood, 2004). CD13 negativity and CD36 positivity was indicative of erythroid cells, with these cells being further analysed for their CD71 and CD235a positivity. Erythroid cells show brighter CD235a positivity in their more mature stages (Wood, 2004).

Fluorophores were chosen to combine all eight CD markers and a viability marker into a single panel of nine colours that could be detected by a BD LSR Fortessa flow cytometer with five lasers (BD Bioscience, San Jose, CA, United States). Due to the use of multiple Brilliant Violet dyes (BD Bioscience), Brilliant Stain Buffer (BD Bioscience) was used in the antibody cocktail to reduce staining artefacts and increase discrimination of cell populations.

### 2.4 Flow cytometry analysis method

#### 2.4.1 Cell preparation

All test cell samples were prepared by centrifuging at 300 × g for 5 min at 4°C and the pellet was resuspended in 450 µL of PBS+0.5% BSA The sample was kept at 4°C for 45 min to allow any ceramic debris to settle.

#### 2.4.2 Staining procedure

Cellular expression of surface antigens CD71, CD16, CD34, CD36, CD235a, CD13, CD41, and CD38 was determined using flow cytometry. A 400 µL aliquot of each prepared test cell sample was added to a labelled 12 × 75-mm capped polystyrene test tube. Alongside the test cell samples, compensation controls using Compbeads Plus (BD Bioscience) and fluorescence minus one (FMO) controls using a designated control cell sample were prepared. The cells were incubated for 30 min at 4°C–8°C in dark with the antibody cocktail containing PE-conjugated mouse anti-human CD71 (BioLegend, San Diego, CA, United States), APC-conjugated mouse anti-human CD16 (BioLegend), APC-Cy7-conjugated mouse anti-human CD34 (BioLegend), BV605-conjugated mouse anti-human CD36 (BD Bioscience), BV786-conjugated mouse anti-human CD235a (BD Bioscience), BV421-conjugated mouse anti-human CD13 (BioLegend), PE-Cy7-conjugated CD41 (BioLegend), and either BUV395-conjugated mouse anti-human CD38 (BD Bioscience) or PE-Dazzle594-conjugated mouse anti-human CD38 (BioLegend). A viability dye, eBioscience Fixable Viability Dye eFluor506 (Life Technologies, Bleiswijk, Netherlands) was also added to each test sample and appropriate FMO control samples. All test and FMO samples were then washed with 1 mL of PBS+0.5%BSA and centrifuged at 300 × g for 5 min at 4°C and 400 µL of PBS + 0.5%BSA added to each sample to reconstitute the pellet. All control and test cell samples were then acquired on the flow cytometer.

#### 2.4.3 Flow cytometry acquisition and gating

All control and test samples were acquired using FACSDiva software on a BD LSR Fortessa (BD Bioscience). Following compensation, all FMO and test samples were acquired at a medium flow rate for either 2 min or until 100,000 events were recorded. The data were gated using FlowJo software version 10.4.1 (Tree Star, Ashland, OR, United States) using the scheme outlined in Figure 2 and Supplementary Figure S1. Doublets and debris based on FSC-A *versus* FSC-H and non-viable cells based on eFluor506 positivity were gated out. An unstained cell sample and the FMO samples served as negatives for each fluorophore, with quadrant gates being set based on <5% positivity in the unstained and appropriate negative FMO sample.

#### 2.4.4 Cell count estimates

Total cell counts for two studies (1 and 2) were estimated using a calculation derived from the single cell count of the sample from the single cell gate, the flow rate through the cytometer (35 μL/min), the time acquired on the cytometer (120 s), and the dilution of the sample. This gave an estimate of the total cell count in the washed and stained sample.

#### 2.4.5 Wright staining and image acquisition

Cells (100 µL) from each of three replicate control sample were combined (300 µL) and transferred onto slides by spinning at 400 × g, 4 min in a Shandon Cytospin three Centrifuge (Thermo Fisher Scientific). Cells were fixed in methanol (−20°C, 15 min) and stored in the dark at −20°C until use. To visually characterise the cells, slides were stained with Wright’s eosin methylene blue solution (Wright’s stain). Slides were incubated in Wright’s stain at RT for 3 min followed by a 6 min incubation in dilute Wright’s stain [distilled water (150 mL), pH 6.8 buffer solution (20 mL), Wright’s stain (30 mL)] and two1 minute washes in pH 6.8 buffer solution and left to dry. A drop of ProLong™ Gold antifade reagent (Thermo Scientific™) was added to each slide and covered with a coverslip. Slides were imaged using an EVOS™ FL Auto two Imaging System microscope (Thermo Fisher Scientific) using the face down single slide setting with on brightfield at ×40 magnification.

### 2.5 Experimental design

Two different approaches to experimental design were used: an independent measures design (BM-1), and a repeated measures design (BM-2 and -3). In both cases, the experimental unit (Lazic et al., 2018) was the circuit, and therefore the sample size was the number of circuits.

For BM-1’s independent measures design, each circuit had two experimental factors (treatment, time) and five potential blocking factors (operator, incubator, control unit, chip, and chip side). It was preferable that both circuits on a chip were harvested at the same time point for practicality reasons–otherwise there was an increased risk of manual error.

BM-2 and -3 had repeated measures designs that used the same experimental factors as BM-1, except there was no longer a need to assign time and hence the design was simpler. Both studies were also required to avoid chips that were already in use in other studies. Thus, the doses were randomised to balance across blocking factors (Supplementary Figures S2A, B).

Furthermore, there are two strategies for managing incubator and operator effects. In BM-2, operator was intentionally confounded with incubator (specifically, one operator was assigned to each incubator) both to minimise the risk of manual error, and also because the smaller number of chips would have made it challenging to block in this way. Hence in this design, operator effects were not able to be separated from incubator effects. The timepoints and doses were randomised to be as balanced as possible across all of the blocking factors, and also to avoid spatial trends where possible (Supplementary Figure S2A).

Whilst BM-2 had operator confounded with incubator, in contrast, we trialled splitting each incubator in two for BM-1 and BM-3, assigning different operators to the different halves. This setup was still practical and allowed deconvolution of the effects of incubator and operator directly.

#### 2.5.1 Analysis approaches

Both count data and percentage data were pre-processed to stabilise their variance for use in linear mixed modelling through addition of a pseudocount and transformation. Percentages had 0.001 added and were logit-transformed, that is, log (p/1-p). Counts had a pseudocount of one added and then were log-transformed.

Three different variance stabilising transformations (VST) on the percentage data were compared, and it was found that a logit transform was best at removing the mean-variance dependency.

Mixed modelling was performed using the lmer () function from lme4 package (v1.1–21). Significance was tested through summary functions in the lmerTest package, using Satterthwaite’s correction. The Nelder-Mead optimiser was used to address convergence issues.

Dimensionality reduction was performed with a Principal Component Analysis (PCA) through the prcomp () function, with scale. = TRUE to remove the effect of variability differing between endpoints. For requirements for a specific article type please refer to the Article Types on any Frontiers journal page. Please also refer to Author Guidelines for further information on how to organize your manuscript in the required sections or their equivalents for your field^1^.

## 3 Results

### 3.1 BM MPS model recapitulates BM cell composition

**FIGURE 3:**
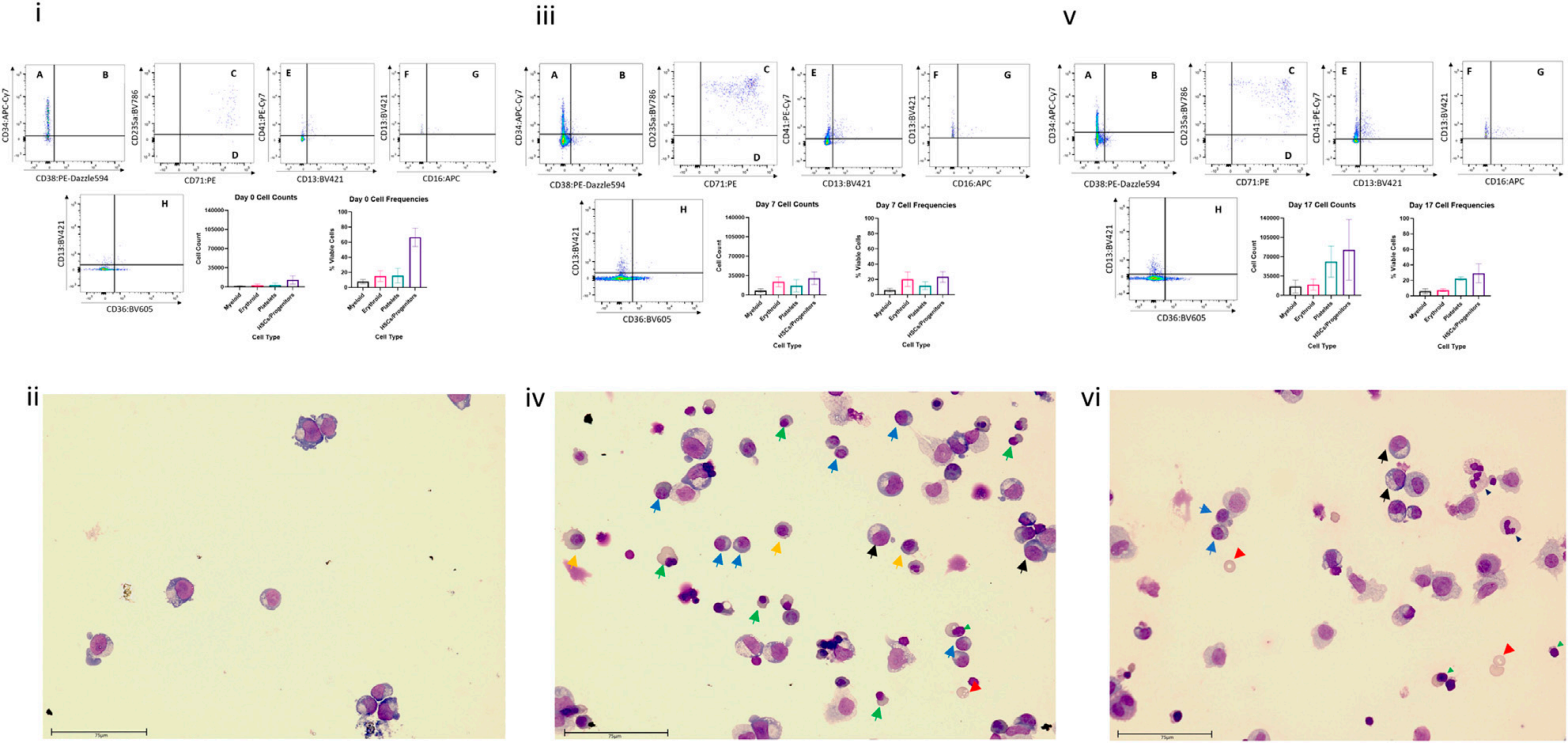
Cell differentiation in the BM MPS. Cells present in the MPS (study BM-3) following increasing culture time (day 7, i and ii; day 14 days, iii and iv; day 24, v and vi) determined by flow cytometry (i, iii,v) and Wright staining (ii, iv, vi). Flow cytometry plots show cells in multiple quadrants expressing the following CD markers: A CD38^−^ CD34^+^, long term HSCs; B CD38^+^ CD34^+^, Lineage Differentiated Progenitors; C CD13^−^ CD36^+^ CD71^+^ CD235a+, Late Erythroid; D CD13^−^ CD36^+^ CD71^+^ CD235a^−^, Early Erythroid; E CD13^−^ CD41a^+^, platelets; F CD16^−^, CD13^+^, Early Myeloid; G CD16^+^ CD13^+^, Late Granulocytes; H CD13^−^ CD36^+^, Late Monocytes. Graphs demonstrate mean values and standard deviations of each lineage in vehicle group circuits. Images show multiple cell types at varying stages of maturation (Green triangle: basophilic normoblasts; red triangle: polychromatic normoblast; orange triangle: orthochromatic normoblast; blue triangle: reticulocyte; black triangle: promyelocyte; light blue triangle: metamyelocyte; purple triangle: band cell; grey triangle: neutrophil). Scale bar 75 µm.

Flow cytometric analysis and Wright staining indicated that the BM MPS drives differentiation of HSPCs predominantly to erythroid and myeloid (neutrophil) lineages, with cells detected at all stages of maturation, whilst maintaining a stem cell pool (Figure 3). Cells from other blood lineages, including platelet lineage cells, were also identified by flow cytometry, although mature megakaryocytes could not be identified by Wright staining. At day 0 (co-culture day 7), approximately 100% of the cells are identified through our flow cytometry gating strategy, however at day 7 and day 17, only about 60% of cells are identified. Our flow cytometry gating did not include markers to identify other cells that could be present in the bone marrow, such as osteoclasts, lymphoid cells, or osteoblasts, which may account for the remaining 40% of cells within the model.

### 3.2 Cells in the scaffold and in the circulating media are comparable

**FIGURE 4:**
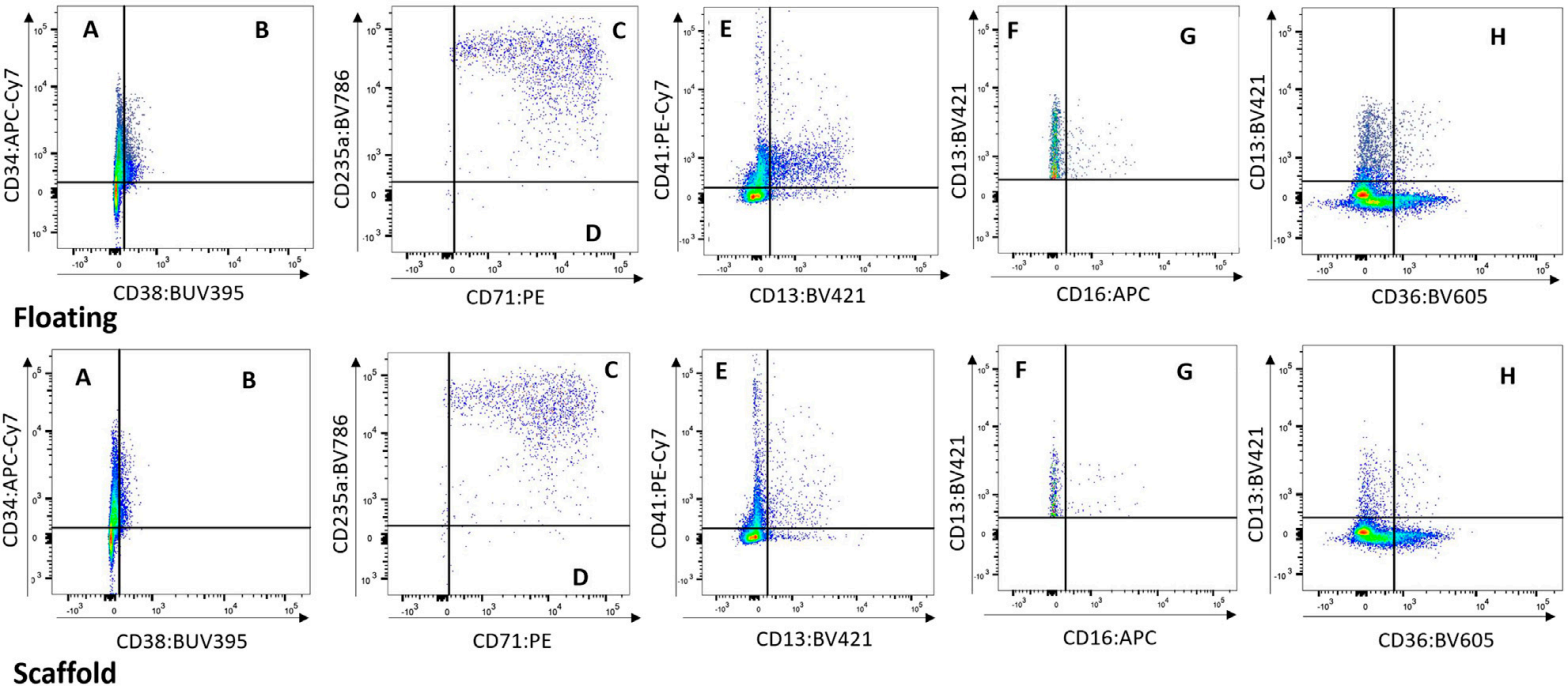
Floating vs. Scaffold Comparison. Example of Floating *versus* Scaffold gating for a control group circuit at Day 14 (study BM-1). **(A)** contains CD38^−^ CD34^+^ cells, called Long term HSCs; **(B)** contains CD38^+^ CD34^+^ cells, called Lineage Differentiated Progenitors; **(C)** contains CD13^−^ CD36^+^ CD71^+^ CD235a^+^ cells, called Late Erythroid cells; **(D)** contains CD13^−^ CD36^+^ CD71^+^ CD235a^−^ cells, called Early Erythroid cells; **(E)** contains CD13^−^ CD41a^+^ cells, called Platelets; **(F)** contains CD16^−^ CD13^+^ cells, called Early Myeloid; **(G)** contains CD16^+^ CD13^+^ cells, called Late Granulocytes; **(H)** contains CD13^+^ CD36^+^ cells, called Late Monocytes.

Cells from the scaffold and media were compared to determine whether the populations present in the scaffold are represented in the media. While a higher number of cells are collected from the scaffold well compared to the media well, the cell population proportions collected from both media and scaffold were very similar (Figure 4 and Supplementary Figure S3). Based on this finding we opted repeated measure design in subsequent studies (BM-2 and-3) where cells were sampled from re-circulating media thereby allowing repeat sampling from same circuit over time.

### 3.3 Dimensionality reduction and overall trends in the data

**FIGURE 5:**
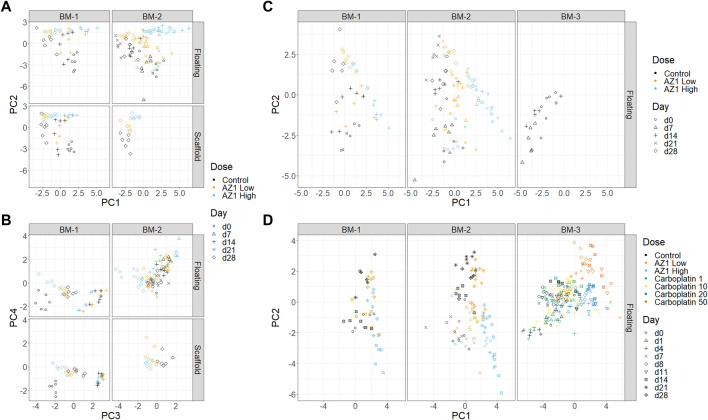
Dimensionality reduction. Principal Component Analysis (PCA) performed on subsets of the three experiments. **(A)** and **(B)** Experiments BM-1 and BM-2 (AZ1 repeats), showing PCs 1 and 2 **(A)** and PCs 3 and 4 **(B)**. **(C)** Experiments BM-1, BM-2 and BM-3 (control only). **(D)** All of the experiments.

PCA is an unsupervised dimensionality reduction method that allowed for determination of which effects dominate the variability seen in the data. The percentage endpoints were used as input for this procedure (i.e., the percentage of cells positive for each single marker, combination of markers, and the percentage of dead cells). As an example, it was noted that the trends across timepoints and concentrations appeared to be similar between floating and scaffold fractions (Figure 5A). This observation supported the claim in the previous section that the dynamics of the scaffold fraction are recapitulated in the floating fraction.

First, to assess for the presence and reproducibility of olaparib’s overall effects across BM-1 and BM-2 experiments, PCA was performed on the combined data from BM-1/BM-2 (Figures 5A, B). Comparable trends were expected in both experiments, because they used the same drug and donor, with the only substantive difference being the repeated measure design. The first two principal components separated the different timepoints and the different concentrations, together accounting for 49.7% of the total variance. Consistent with our expectation, high concordance was seen between the two experiments. Meanwhile, PC4 separated the two experiments, accounting for 8.6% of the variance. Since the inter-experiment variability was low compared to the other principal components, this indicates that overall the technical variability is small compared to these effects. Moreover, the results showed confidence in the repeated measures design. Thus, our characterisation is robust to the change from independent measure to repeated measure design.

Second, donor variability was investigated by adding the control group from BM-3 to the full BM-1/2 data (taking only the timepoints that match between the experiments) and performing a new PCA (Figure 5C). A difference between the two donors was indeed observed, with BM-3’s chips clustering near the later timepoints of BM-1/2. However, this donor variability appeared to be in a different direction to the effect of the drug, and hence the effect of the drug can be deconvoluted from the donor variability (note that the behaviour of the late erythroid lineage is comparable between studies; Figure 7). This showed that using different donors could be important when performing routine studies, which was taken into account later.

Finally, the effects of the two drugs were compared across the three experiments by performing PCA on all of the data together (BM-1,2,3) (Figure 5D). The analysis was consistent with known information about the drugs: PC1 aligns with increasing concentrations of both drugs, and also late erythroid death. In contrast, the drugs diverge in PC2, which is aligned with platelet count; it is known from clinical results that carboplatin has an effect on platelets that differs from olaparib. However, since many factors change between these experiments, it would be unwise to use this as the sole supporting evidence.

### 3.4 Data modelling considerations

Two ways to analyse the data were evaluated–through the raw counts, and through the percentages. Both approaches involved a variance-stabilising transformation and pseudocount (see Methods).

In our experience, the count data approach was usually more appropriate for detecting toxic effects at a given timepoint. However, counts are not comparable across timepoints directly, because the growth of the overall population may need to be taken into account. The percentage data may help determine cell types that deviate from the average growth rate, if these effects are not visible in the count data. However, if 1 cell population decreases in size, the percentages of all of the others go up. Hence, a small decrease in relative population size can be masked by a larger one.

### 3.5 Assessment of technical effects

The impact of technical sources of variation on the data was assessed, focussing on the variability between operator, incubator, control unit, chip and side of chip. This assessment was important, firstly, because experiments should be designed in such a way that technical effects are not confounded with the biological effects of interest; secondly, understanding and quantification these effects can be used to monitor the quality and performance of the assay, and thirdly, regressing out these components helps improve the experimental power.

**FIGURE 6:**
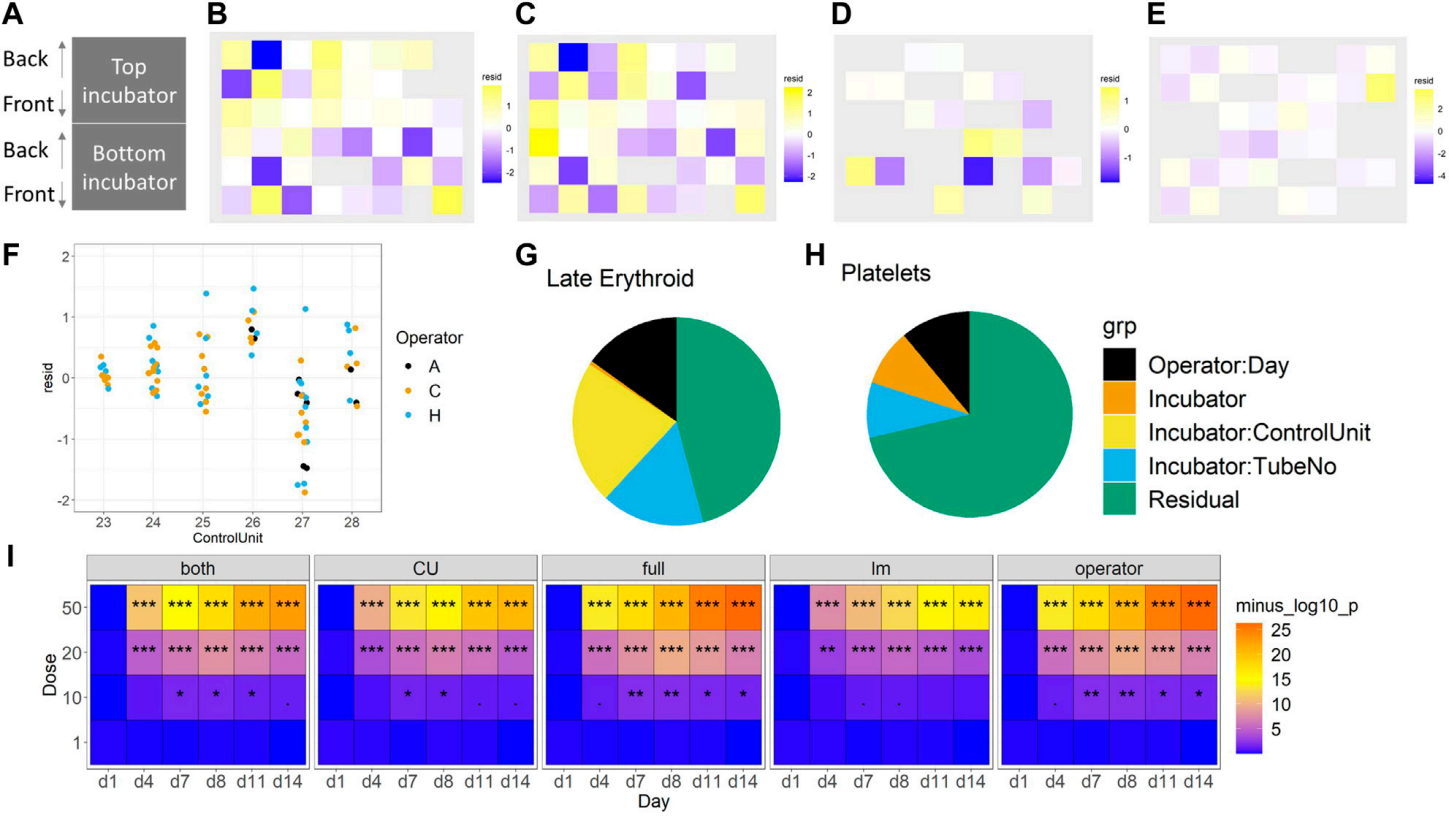
Consideration of technical effects (F vs. S). The impact of incubator position **(A)** on the late erythroid population (%) for BM-1 and BM-2 for floating **(B)** and **(D)** and scaffold **(C)** and **(E)** cells, and operator **(F)**. The effect of these variables on the erythroid lineage **(G)** and platelet lineage **(H)** (%) is shown. The sensitivity of the different models to toxicity (*p*-value significance level) is shown in **(I)**. For **(B–E)**, purple, lower than expected; yellow, higher than expected. For I, significance values are (<0.1), * (*p* < 0.05), ** (*p* < 0.01), *** (*p* < 0.001).

**FIGURE 7:**
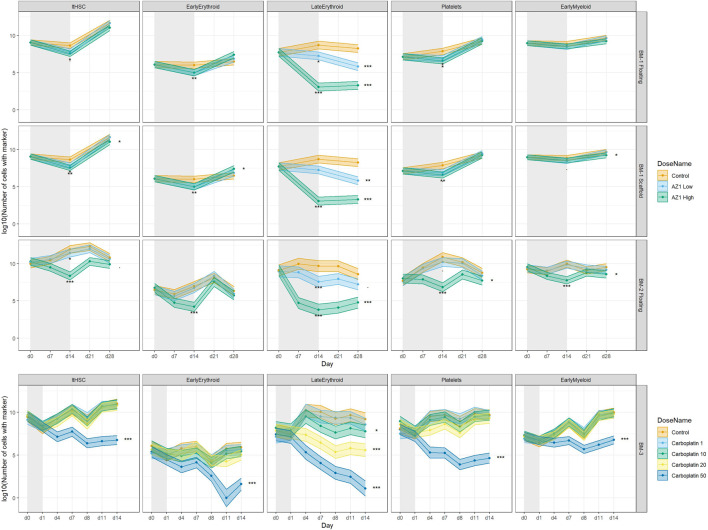
Treatment-induced lineage-specific toxicity. The effect of treatment with AZ1 or carboplatin on different haematopoietic lineages over time in BM-1, BM-2 or BM-3 studies. The shaded regions represent the dosing period in each case. Stars represent the results of the significance tests, at both the end of the dosing period and the end of the recovery period for each drug (note that carboplatin showed no significant effects at the end of the dosing period), Significance values are (<0.1), * (*p* < 0.05), ** (*p* < 0.01), *** (*p* < 0.001).

Fixed effects of technical factors. Dead cell percentage and late erythroid percentage were modelled, as these were two key variables of interest. To focus on the technical effects, removing the biological effects of time and concentration by “regressing them out” to obtain residuals was attempted (see linear modelling in Methods). These residuals were then explored in two ways. First, the residuals spatially within the incubator were plotted to check for a gradient effect; no gradient was observed (Figures 6A–E). Then, linear regression was used to check for dependencies on other technical effects. Two models were compared, both of which had additive effects of operator and side, but one had an incubator effect and the other a control unit effect (Figure 7 and Supplementary Figure S4). Since the control unit model is nested within the incubator model, an ANOVA test tells us if the control unit term is justified over and above the incubator effect. Then, in the appropriate model, the effects of operator and side can be tested, as well as the incubator or control unit as appropriate. Across all of the experiments, no evidence of a left/right side effect was found. Operator effects were not significant at the 5% level, but frequently close to it (Figure 6F). This would indicate that operator effects are present, but weak, since: 1) the operator term was significant when the control unit model was used, and 2) a *t*-test indicated that one of the operators had a significant change from the mean. However, there is strong evidence for incubator effects on the late erythroid cell percentage (Figure 6G), which is not seen for the platelets (Figure 6H), and control unit effects on the early erythroid percentage, especially in the floating fractions of BM-2 and BM-3 (Supplementary Figure S5).

Decomposition of variance using a mixed model. The technical effects were elucidated further using a mixed model approach. The BM-3 data set was used for this purpose, because it had the most samples and the best representation across technical factors. The advantages of a mixed model approach are that it naturally incorporates nesting structures, and it allows us to quantify variability and compare it across factors. A series of different model structures were explored to find the most appropriate for the data (Table 3). Models used a selection of random effects, taking account of incubator, control unit, circuit, or flusher as appropriate. First, how the variability is divided between each of these technical factors was explored. Figures 7G–H shows the variance decomposition for two representative endpoints. In each case a substantial fraction of the variance was explained by technical effects. Importantly, the decomposition varies between endpoints. For example, the Late Erythroid showed a much higher effect of control unit than the other endpoints did, such as the platelet lineage (with this effect manifesting as between-circuit variability in the operator model). Overall, this indicated that operator, incubator and control units can all affect the data, but this effect should not be assumed consistent over different endpoints and different experiments. In our experience, the partition of technical effects can vary considerably between experiments. Hence, designs should block over these variables to ensure like is compared with like. When assessing different metrics to test models for fit, the “full” model (that is, the one with the most endpoints) performed the best (Figure 7I; Table 3).

**TABLE 3:** Models used, with measures of fit and terms included.

Model	Degrees of freedom		BIC		Log-likelihood		Model formula in lmer () notation					
Linear	36		774.78		−291.31		log (value +1) ∼ factor (Dose)*Day					
Unit	38		738.92		−268.05		log (value +1) ∼ factor (Dose)*Day + (1 | Incubator) + (1 | Incubator:ControlUnit)					
Flusher	38		773.37		−285.27		log (value +1) ∼ factor (Dose)*Day + (1 | Incubator) + (1 | Operator:Day)					
Both	39		736.87		−264.35		log (value +1) ∼ factor (Dose)*Day + (1 | Incubator) + (1 | Incubator:ControlUnit) + (1 | Operator:Day)					
Full	40		721.97		−254.23		log (value +1) ∼ factor (Dose)*Day + (1 | Incubator) + (1 | Incubator:ControlUnit) + (1 | Incubator:TubeNo) + (1 | Operator:Day)					
Term		Effect Type		Linear model		Unit		Operator	Both	Full	Comment	
Dose*Day		Fixed		X		X		X	X	X	Equivalent to Dose + Day + Dose:Day	
Incubator		Random				X		X	X	X		
Incubator:ControlUnit		Random				X			X	X		
Incubator:Circuit		Random								X	Accounts for repeated measures from the same circuit	
Operator:Day		Random						X	X	X		

### 3.6 Testing for effects

A simple fixed effect model was tried and compared against the mixed model approach. The mixed model tests for significance whilst taking account of the correlation structure induced by the technical effects. We use the full operator and circuit model because it has the best resolution. It was found that significance of differences between treatment and control was stronger in the mixed effect model than the fixed effect model (Supplementary Figure S5). In future studies, where more circuits are present, a model with more elements may be merited.

Treatment with olaparib and carboplatin produce expected lineage-specific toxicities. Treatment with olaparib was expected to induce predominantly erythroid toxicity, with some myeloid (neutrophil) toxicity at higher concentrations (Olaparib prescribing information, 2022). Cell number results from both BM-1 (independent measures) and BM-2 (repeated measures) showed olaparib induces toxicity against the CD71^+^ CD235+ erythroid cells (Late Erythroid) in both the scaffold and media at the concentrations tested (Figure 6). Additionally, the repeated measures design (BM-2) identified toxicity against Early Erythroid, Early Myeloid and platelet lineages and LTHSCs at the high dose (Figure 6).

Carboplatin typically induces pancytopenia (Cheng et al., 2017). Cell number results from BM-3 (repeated measures) show a dose-dependent toxicity against Early Erythroid and the platelets, with toxicity against LTHSCs, Early Myeloid and Late Erythroid following treatment with 50 µM carboplatin only (Figure 6).

Note that removing terms from the model (that is, using a model other than the ‘full’ one) decreased sensitivity for detecting the toxic effects.

### 3.7 Power analysis

Having established the repeated-measures study design, a power analysis was conducted to determine the number of circuits (replicates) required to robustly detect toxicity using two different drugs at multiple concentrations and timepoints. The analysis suggests that four circuits are sufficient to detect toxicity induced by both concentrations of olaparib, and by 20 or 50 µM carboplatin, while for 10 µM carboplatin, which only induced low level toxicity against erythroid cells, 6-7 circuits were required to detect this (Supplementary Figure S6). The number of replicates used should therefore account for the expected level of toxicity, and also allow for attrition, such as chip failure.

### 3.8 Summary of recommendations

Based on the assessments of these three studies, we recommend using a randomised block design, with n = 6 circuits per condition based on repeated measures (although it can be dropped to n = 5 in order to explore more conditions). We of protecting against these technical effects and of using a mixed model over a fixed effect model where applicable. We propose a standard experimental design that protects against technical effects (Supplementary Figure S7).

## 4 Discussion

This paper, to the best of our knowledge, describes the first application of a design of experiments (DoE) approach to BM organ-chip studies. Given the complex nature of organ-chip studies, there are a number of potential confounding factors that need to be accounted and controlled, to ensure that results are reproducible, and conclusions drawn are robust. Recently, a statistically robust DoE setup was described for the imaging of organ-chips, accounting for chip, row, and holder effects (Peel et al., 2019). In contrast, we focus on additional factors during the experimental phase that could introduce variability: cell donor, the operator maintaining and sampling the chips, the incubator, the control unit to which the chips are connected, the side/circuit of the chip, and the cells analysed (media *versus* scaffold). Applying a DoE approach to BM organ-chip studies for a defined safety-oriented context of use ensures robust and reproducible data and ultimately drives confidence in the model. In the current study, to test the robustness of the experimental design and ability of the model to detect toxicity, chips were treated with either carboplatin or olaparib, each of which has different lineage-specific toxicities.

### 4.1 Cells in the media are a good proxy measure for the scaffold-resident cells

Cells can be sampled from the chips by flushing them from the scaffold or collecting them from the re-circulating media. The cell populations in the media are similar to the cells present in the scaffold, as confirmed by flow cytometry (Figure 4). This should not be mistaken for cell populations in the media representing a proxy for the peripheral blood (as happens *in vivo*, where the more primitive HSCs/HSPCs reside within the niches of the bone marrow, while the mature cells move into the peripheral circulation). The numbers of cells are higher in the scaffold samples than in the floating samples, but proportionally the cell numbers are the same and allow for differentiation in treatment groups. Further support was provided by the PCA of the two populations, which indicated similarity, with the proximity of erythroid cells from both scaffold and media population being comparable. Therefore, the cells in the media represent a proxy for the bone marrow, providing an opportunity to sample the ‘bone marrow’ over time, which is not possible *in vivo* and with BM-on-chip models based on hydrogel co-culture. Importantly, the response of the erythroid cells in both populations to treatment with olaparib is similar. This indicates that treatment effects can be detected by measuring cells from the media alone, making it possible to undertake a repeated measures approach. In contrast, in the independent measures design, where cells were sampled from the scaffold, requiring a different circuit for each timepoint, the expected erythroid lineage toxicity with olaparib was observed with a similar trend (both BM-1 and-2). However, toxicity for the Early Myeloid cells with olaparib was only observed in the repeated measures study design (BM-2), suggesting that inter-chip variability could reduce the sensitivity of the model, masking small drug effects. This is important when considering the application of this model for safety assessment. Another advantage of the repeated measures design is that since the number of chips does not scale with timepoints, more timepoints can be analysed. This improves the temporal resolution of the data, as demonstrated following treatment with olaparib; toxicity against the erythroid lineage was observed at day 14 in both study designs, but the inclusion of additional timepoints in the repeated measures design indicates that toxicity is also observed at day 7. This provides a more accurate indication of the time taken for toxicity to occur, and recovery of the cells post-treatment, which is critical for oncology drug safety assessment. Since fewer chips are required for a repeated measures design, this approach also affords greater flexibility in experimental design, allowing more conditions to be tested, such as additional drug concentrations, which would improve our understanding of the dose-response relationship. Moreover, by sampling cells from the media of each circuit, any individual circuit effects can be regressed out, as all circuits are first measured pre-dosing (D0).

### 4.2 Power analysis

A DoE approach can also improve the power to detect potential adverse effects, as the mixed model analysis approach showed stronger significance than the fixed effect model. This will not always be the case, however, as in other situations, variability of technical effects could be mistaken for drug effects, and so the mixed model may mitigate false positives by making the *p*-values larger.

Currently, routine studies are run with six replicates (circuits), and the data suggest this could be reduced for studies designed to detect larger-magnitude effects. The repeated-measures design improves the power compared to sampling either the scaffold or media cells in the independent measures study. The analysis suggests three replicates are sufficient to detect the effect achieved at the low and high dose of olaparib when making repeated measures, whereas five replicates were required to detect only the high dose-induced effect in the independent measures study. Reducing the number of replicates would increase the number of conditions that could be tested in a single study. If the magnitude of effect is unknown, or smaller effect sizes are expected, then more replicates may be required. This may advocate for a study design incorporating different numbers of replicates according to the drug being tested and the knowledge of its effects and highlights the need for power calculations prior to studies being conducted to ensure drug effects can be robustly detected.

### 4.3 Implications of donor variability

Since this model has been developed using primary human cells, the effect of donor variability is important to understand two key points; (i) how representative one donor is to a population, (ii) what is the optimum number of donors to test. In the current study, two donors were compared and although differences could be seen using PCA according to the direction of the clustering, importantly these differences did not prevent drug effects from being detected. In support, Peel et al. (2019) tested two donors and indicated that donor-to-donor variability is negligible compared to other sources of variability and therefore excluded donor as a descriptor in the statistical model. However, it should be noted that donor differences may be important for other drugs or treatments, such as ionizing radiation, since sensitivity can vary between patients. Thus, further data are required to give confidence that donor-to-donor variability is not a significant variable in organ-on-chip studies; Sieber et al. (2018) also tested multiple donors (four independent donors); however, there was no specific investigation of donor-to-donor variability and its potential impact on cell composition in the model or drug effects. A challenge with testing multiple donors in MPS studies is the cost and time implications associated with this, as well as cell availability. While cell lines could represent an alternative to primary cells owing to their ease of access, they are immortalised, often cancer-derived, and may have genetic or epigenetic drift from culturing over multiple generations (Nestor et al., 2015; Horvath et al., 2016; Low and Tagle, 2017). Moreover, the goal is for these models to have increased human/patient relevance, thus cell lines are less favourable. More recently, iPSCs are being used to establish organ-chip cultures, which have advantages over primary cell use such as having a renewable cell source from the same donor (Low and Tagle, 2017). Where primary cells are being used, the number of donors tested should be considered when designing future studies to ensure consistent effects.

### 4.4 Discussion of other experimental design factors

A number of other confounding factors were also investigated. The side of the chip does not influence the results; however, control unit, incubator and operator were all found to have an effect on the results observed. We saw an effect of control unit on the Late Erythroid cell number in the floating fractions of BM-2 and BM-3, but the reason for this is unclear. The control unit defines the vacuum and pressure that drives the flow rate, and while this is set to the same value for each control unit, it is possible that there are some differences in the accuracy between units. The reason for the incubator effects on the number of Late Erythroid cells is also unclear but could be due to fluctuations in temperature and/or CO_2_ percentage relative to the other incubator used. Monitoring incubator readouts and/or calibration records during studies would help identify such factors. Importantly, designing the experiment in this way allows for these differences to be detected and subsequently investigated and any problem with the equipment fixed, which would otherwise not be possible. Moreover, the impact of control unit and incubator on the data highlight the need for a design of experiments approach, as described in this manuscript, as this enables these effects to be regressed out, leaving only the true drug-induced effects.

An impact of operator was also observed across the four scientists involved in these studies. This is not surprising, since 3D/MPS cultures are technically more challenging to maintain than standard 2D culture, therefore necessitating higher training requirements. It should be noted, however, that even with training, it is expected there will still be differences in technique between individuals, increasing the chance of technical variability. Experience of the scientist also plays a role. In support, the current study shows that with training and experience, operator effects can decrease. However, the data indicate that while these factors can have an effect on the data, they are not consistent across timepoints or studies, highlighting the importance of using an experimental design that is cognisant of these effects. While automating chip handling could improve reproducibility, this could introduce other confounders, and it is important to note that drug effects were detectable despite the operator variability when an appropriate design was used.

We observed some differences in interaction between technical effects and endpoints. Most notably, there was more between-control unit variability in the Late Erythroid population than in other lineages. This effect may be because the erythroid cells are expected to circulate more than the other cell types. The observation supports the strategy of independently fitting statistical models to each endpoint.

### 4.5 Drug-induced toxicity detection in the BM MPS

Having optimised the experimental conditions, the BM toxicity induced by olaparib and carboplatin was investigated. Treatment with olaparib induced predominantly erythroid toxicity, in both the individual chip and repeated measures designs, which was in line with the expected lineage-specific response for this drug (Lynparza prescribing information, 2022). Importantly, when a repeated-measures study design was used, toxicity against Early Myeloid cells, platelet lineage cells and LTHSCs was also detected, which was expected for the high concentration used, but was missed in the individual measures chip design. These data suggest that the BM MPS recapitulates the human BM sufficiently to enable detection of lineage-specific toxicities, and that the repeated measures study design increases the sensitivity, enabling toxicities of smaller-magnitude to be detected (Supplementary Figure S6). The repeated measures design also enables additional timepoints to be included, since one circuit per replicate is used for the entire study duration rather than per timepoint. In contrast, for an independent measures study where chip capacity is fixed, conditions must be sacrificed in order to accommodate more timepoints. Hence, a repeated measures design can improve the temporal resolution of the toxicity response, which is critical for determining time for response and recovery. Moreover, toxicity was detected against different maturation stages of the lineages, such as Early Myeloid or Late Erythroid, which is advantageous over current *in vivo* bone marrow analytical capabilities, where only broad erythroid, lymphoid, myeloid, and platelet lineage toxicity is reported (Saad et al., 2000). This gives us granularity when testing compounds that may only have an effect on certain stages of maturation; If a compound is only expected to have an effect earlier in the haematopoietic maturation, changes in the progenitor populations should be demonstrated, whereas if there are only effects on the more terminally differentiated populations, only those populations should show changes. Moreover, if toxicity only affects a subset of cells, this effect could be masked if looking at the lineage population as a whole, rather than at the finer granularity of individual maturation stages. The maintenance of a stem cell population and the ability for extended viable cell culture in the MPS enables the detection of recovery. As observed following treatment with olaparib, upon cessation of dosing, the Early Myeloid, Early Erythroid and platelet lineages, and LTHSCs recover back to baseline.

Further supporting the capability of the model to detect lineage-specific toxicity, carboplatin has been shown to induce pan cytopenia (Cheng et al., 2017). Specifically, loss of erythropoiesis and decreases in megakaryocytes and immature myeloid cells was observed in the BM of rats (Ohno et al., 1991), while in humans, myelosuppression together with thrombocytopenia were dose-limiting (Go and Adjei, 1999). These toxicities were observed in the BM MPS in the current study, with carboplatin inducing dose-dependent erythroid and megakaryocyte toxicity, and pan-lineage toxicity at the high dose (50 µM), suggesting that our BM MPS sufficiently recapitulates the human BM to detect these lineage-specific toxicities. This system maintains stem cells, which are expected to be functional as reported by Sieber et al. and Chou et al., however functional assays would be important to confirm that in this system. The presence of these cells enables toxicity against them to be detected; indeed, carboplatin induced stem cell toxicity following treatment with 50 µM, which has been reported for this drug in mice (van Os et al., 1998). The maintenance of BM stem cells in differentiation media for this length of time is not currently possible in 2D culture as all cells terminally differentiate (Glettig and Kaplan, 2013). It has been reported that the maintenance of undifferentiated pluripotent stem cells in culture is challenging (Ertl et al., 2014), likely because 2D culture conditions lack the features required to mimic the *in vivo* microenvironment, and stem cell maintenance requires multiple cues from the microenvironment. The 3D microenvironment is responsible for the regulation of stem cell fate *in vivo* since it enables complex interactions between cells, extracellular matrix, and provide gradients of nutrients, oxygen, and waste (McKee and Chaudhry, 2017). The MSC-seeded scaffold in this model has been demonstrated to create a microenvironment suitable for HSPC culture, including fibronectin deposition, and expression of genes associated with the BM niche *in vivo* such as nestin and osteopontin 1. A further advantage of this BM MPS is that all lineages are present and can therefore be tested in one system, whereas in current *in vitro* tests they are looked at in isolation. Toxicity against one lineage, or maturation stage, may influence another lineage, and this can be detected in our BM MPS. The ability to determine toxicity against maturation stage is also an advantage over current *in vivo* study designs, where only broad lineages are measured.

We expect to see variable patterns of toxicity with different classes of compound, as some compounds cause only acute myelosuppression, such as with methotrexate, whereas others can induce residual, long-term BM injury, such as is seen with carboplatin (Wang et al., 2006). With our model, we plan to extend our experimental design to future studies that can explore these variable effects on the BM with different compounds as well as with combinations of compounds.

### 4.6 Positioning of the BM MPS in safety assessment

The maintenance of a viable cell population for an extended period (>5 weeks) that encompasses stem and progenitor, erythroid, myeloid and megakaryocyte cells in this model, along with the ability to undertake repeat sampling from a single circuit makes the BM MPS particularly suited for longer-term studies. This allows for toxicity and recovery to be measured within studies, which is critical for assessing the BM’s ability to rebound after any compound-related toxicity. This BM MPS could therefore be an extremely valuable tool to flag potential toxicity to inform future *in vivo* studies and clinical trials.

Due to the model’s applicability towards informing *in vivo* studies, this could have an implication for 3Rs, since measuring the dynamics of BM response necessitates large numbers of animals. As differential cell analysis of rodent bone marrow is a terminal procedure, the number of animals utilized in a study must be scaled to the timepoints of interest if a temporal response is desired. Provided we take into consideration differences between models, in terms of BM composition and CD markers used to identify cell populations, the BM MPS model described here is reflective of the human BM composition and so translatability to the clinic may be improved over rodent BM analysis. Hence, by predicting BM toxicity using the MPS ahead of *in vivo* investigations, a reduction in the number of animals required for a pre-clinical investigational toxicity study could be achieved; depending on the conditions tested and responses seen in the MPS the study design can be influenced to reduce the number of conditions required to be tested *in vivo*.

## 5 Conclusion

We have established a best practice for experimental design in a BM MPS applied to toxicity testing of oncology drugs, but many of the confounding factors addressed in the current study are applicable to other MPS. As such, this is a best practice that can be applied to the MPS field more broadly. While further improvements could be made to the analysis, this approach has taken into consideration many aspects of the experimental design to reduce variability and improve the power to detect toxic effects. This design of experiments approach has established a groundwork for a reliable and reproducible *in vitro* analysis of BM toxicity in a MPS.

## Data Availability

Original datasets are available in a publicly accessible repository: http://flowrepository.org/ under accessions FR-FCM-Z5E8, FR-FCM-Z5ET and FR-FCM-ZFS. Original code used for this study can be found in the Supplementary Material.

## Footnotes

1 For Original Research articles, please note that the Material and Methods section can be placed in any of the following ways: before Results, before Discussion or after Discussion.

## Supplementary material

Supplementary Figure S1 Detailed Gating strategy. **(A)** Debris and doublets are excluded outside the Single Cells gate; **(B)** eFluor506 positive non-viable cells are excluded; **(C)** CD41 CD13 cells are considered Platelet Lineage Cells; **(D)** CD38 CD34 cells are considered Long term HSCs and CD38 CD34 cells are Lineage Differentiated Progenitors; **(E)** CD36 CD13 cells are considered Early Myeloid cells. CD36 CD13 are considered Late Monocytes. CD36 CD13 cells are gated out as erythroid cells and then analysed for CD71 vs CD235a positivity; **(F)** CD13^+^ cells are gated and analysed for CD16 vs CD13 positivity. CD16 CD13 cells are considered Early Myeloid cells. CD16 CD13 are considered Late Granulocytes.

Supplementary Figure S2 Experimental layout. Experimental design of BM-2 **(A)** and BM-3 **(B)**. LHS, left hand side circuit; RHS, right hand side circuit. For **(A)**, Shaded circuits still in use. FMO controls for FACS analysis. Flusher A: Top incubator (circuits 1-24). Flusher B: Bottom incubator (25-48). For **(B)**, VACANT circuits in use; numbers are different treatments.

Supplementary Figure S3 Floating vs Scaffold Comparisons. Further examples of floating versus scaffold gating within individual circuits across varying timepoints and dose groups in BM-1. **(A)** control group circuit at Day 0, **(B)** control group circuit at Day 14 Recovery, **(C)** low dose group circuit at Day 14, **(D)** high dose group circuit at Day 14, **(E)** low dose group circuit at Day 14 Recovery, **(F)** high dose group circuit at Day 14 Recovery. Flow cytometry scatter plots gated for the following cell types: A - CD38- CD34^+^ cells, called Long term HSCs; B - CD38^+^ CD34^+^ cells, called Lineage Differentiated Progenitors; C - CD13^-^ CD36^+^ CD71^+^ CD235a^+^ cells, called Late Erythroid cells; D - CD13^-^ CD36^+^ CD71^+^ CD235a^-^ cells, called Early Erythroid cells; E - CD13^-^ CD41a^+^ cells, called Platelets; F - CD16^-^ CD13^+^ cells, called Early Myeloid; G - CD16_+_ CD13^+^ cells, called Late Granulocytes; H - CD13^+^ CD36^+^ cells, called Late Monocytes.

Supplementary Figure S4 Fixed effects of technical factors. Determining the significance of the effect of technical variables on **(A)** dead cell percentage or **(B)** Late Erythroid percentage.

Supplementary Figure S5 Consideration of technical effects. Control unit and flusher model for BM-3. Comparison of a fixed- and mixed-effect model. Significance values are: (*p* < 0.1), * (*p* < 0.05), ** (*p* < 0.01), *** (*p* < 0.001).

Supplementary Figure S6 Power calculation. Determination of the number of replicates required to determine a specific effect size for BM-1, BM-2 and BM-3.

Supplementary Figure S7 Recommended layout for bone marrow MPS experiments. LHS, left hand side circuit; RHS, right hand side circuit. **(A–G)** are treatment conditions.
