# Supplementary material for: Optimal experimental design for efficient toxicity testing in microphysiological systems: A bone marrow application
Source: Front Pharmacol. 2023 Mar 31;14:1142581. doi: 10.3389/fphar.2023.1142581 (PMC10103791; doi:10.3389/fphar.2023.1142581)
Supplement: Supplementary file 1 [file Presentation1.pdf]

Supplementary Figure 1

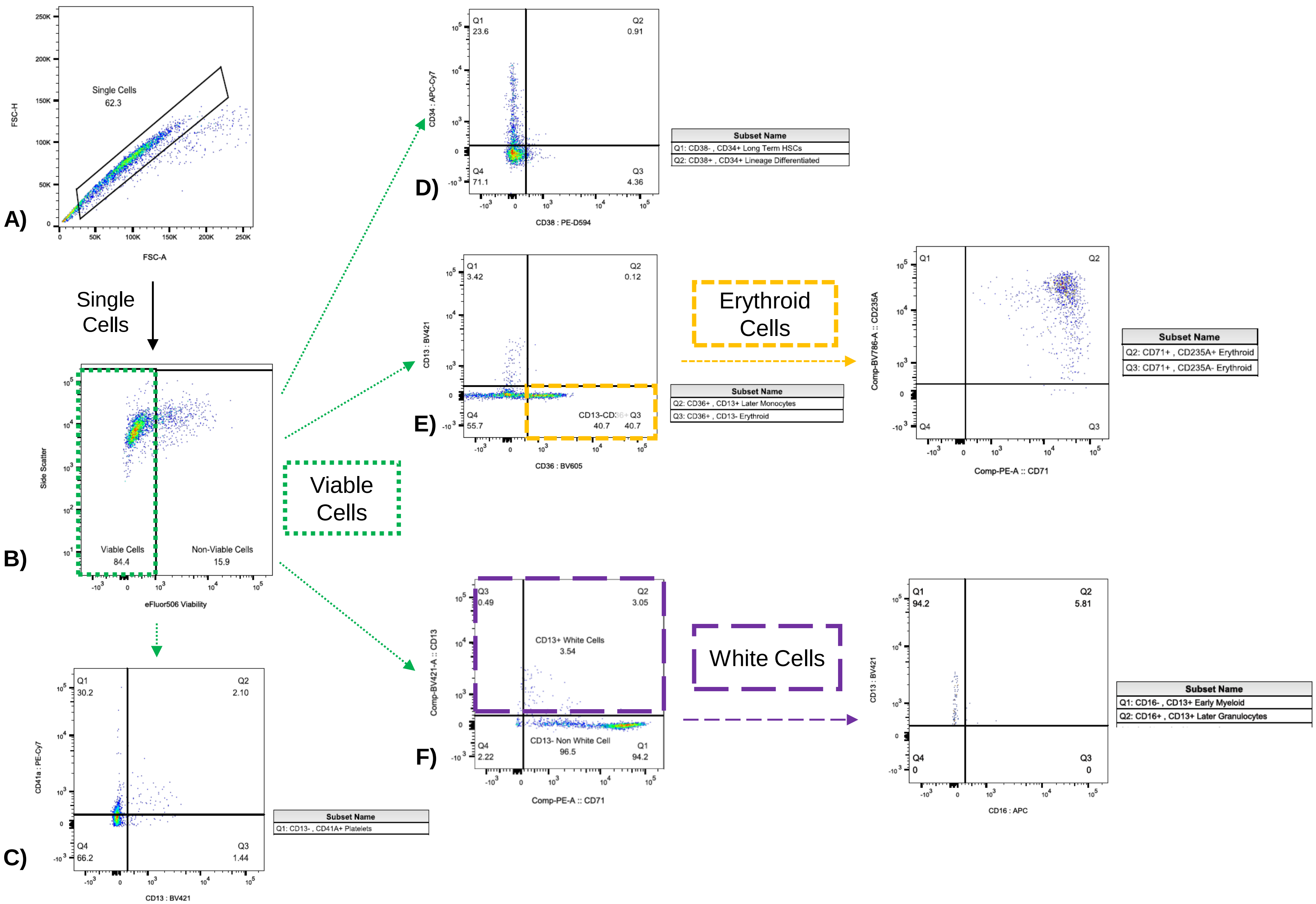

Supplementary Figure 2

A

	Circuit LHS	Circuit RHS
Treatment	-	-
Circuit #	1	2
Control Unit	23	23
Treatment	Dose 2	Dose 1
Circuit #	3	4
Control Unit	23	23
Treatment	-	-
Circuit #	5	6
Control Unit	23	23
Treatment	T1 FMO	T2 FMO
Circuit #	7	8
Control Unit	23	23
Treatment	Control	Dose 2
Circuit #	9	10
Control Unit	24	24
Treatment	-	-
Circuit #	11	12
Control Unit	24	24

	Circuit LHS	Circuit RHS
Treatment	Control	Dose 1
Circuit #	13	14
Control Unit	24	24
Treatment	-	-
Circuit #	15	16
Control Unit	24	24
Treatment	-	-
Circuit #	17	18
Control Unit	25	25
Treatment	Dose 2	Control
Circuit #	19	20
Control Unit	25	25
Treatment	-	-
Circuit #	21	22
Control Unit	25	25
Treatment	Dose 1	-
Circuit #	23	24
Control Unit	25	25

	Circuit LHS	Circuit RHS
Treatment	-	-
Circuit #	25	26
Control Unit	26	26
Treatment	-	-
Circuit #	27	28
Control Unit	26	26
Treatment	Control	Dose 1
Circuit #	29	30
Control Unit	26	26
Treatment	-	-
Circuit #	31	32
Control Unit	26	26
Treatment	Dose 2	Control
Circuit #	33	34
Control Unit	27	27
Treatment	-	-
Circuit #	35	36
Control Unit	27	27

	Circuit LHS	Circuit RHS
Treatment	Dose 2	T3 FMO
Circuit #	37	38
Control Unit	27	27
Treatment	Dose 1	Control
Circuit #	39	40
Control Unit	27	27
Treatment	-	-
Circuit #	41	42
Control Unit	28	28
Treatment	T4 FMO	Dose 1
Circuit #	43	44
Control Unit	28	28
Treatment	-	-
Circuit #	45	46
Control Unit	28	28
Treatment	Dose 2	T5 FMO
Circuit #	47	48
Control Unit	28	28

B

Top Incubator	Circuit LHS	Circuit RHS	Operator
Treatment	50	500	
Circuit #			A
Control Unit			
Treatment	10	0	
Circuit #			A
Control Unit			
Treatment	VACANT	VACANT	
Circuit #			A
Control Unit			
Treatment	100	50	
Circuit #			A
Control Unit			
Treatment	500	10	
Circuit #			A
Control Unit			
Treatment	VACANT	VACANT	
Circuit #			A
Control Unit			
Treatment	0	100	
Circuit #			B
Control Unit			
Treatment	50	500	
Circuit #			B
Control Unit			
Treatment	VACANT	VACANT	
Circuit #			B
Control Unit			
Treatment	10	0	
Circuit #			B
Control Unit			
Treatment	100	50	
Circuit #			B
Control Unit			
Treatment	VACANT	VACANT	
Circuit #			B
Control Unit			

Bottom Incubator	Circuit LHS	Circuit RHS	Operator
Treatment	VACANT	VACANT	
Circuit #			B
Control Unit			
Treatment	500	10	
Circuit #			B
Control Unit			
Treatment	0	100	
Circuit #			B
Control Unit			
Treatment	VACANT	VACANT	
Circuit #			B
Control Unit			
Treatment	50	500	
Circuit #			B
Control Unit			
Treatment	10	0	
Circuit #			B
Control Unit			
Treatment	VACANT	VACANT	
Circuit #			A
Control Unit			
Treatment	100	50	
Circuit #			A
Control Unit			
Treatment	500	10	
Circuit #			A
Control Unit			
Treatment	VACANT	VACANT	
Circuit #			A
Control Unit			
Treatment	0	100	
Circuit #			A
Control Unit			
Treatment	VACANT	VACANT	
Circuit #			A
Control Unit			

Supplementary Figure 3

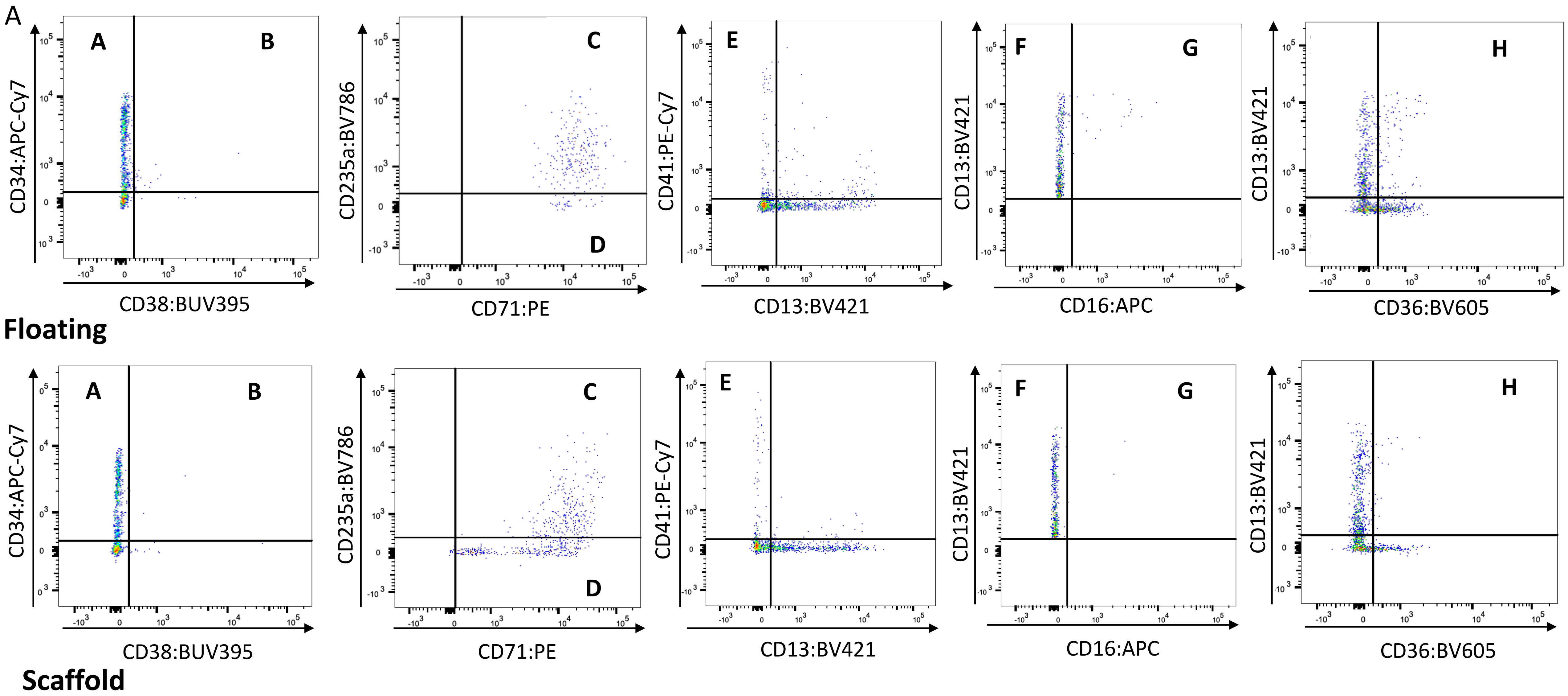

Example of Floating versus Scaffold gating for a control group circuit at Day 0.

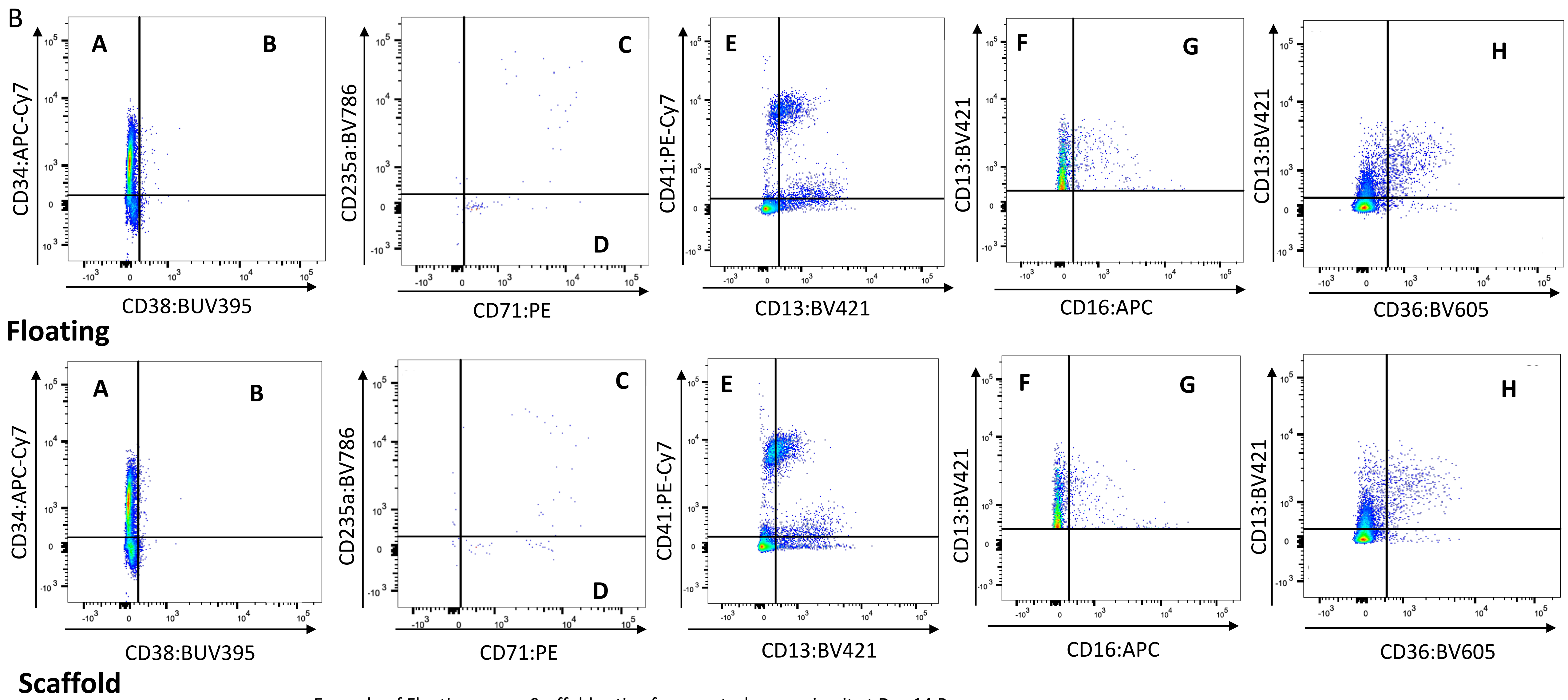

Example of Floating versus Scaffold gating for a control group circuit at Day 14 Recovery.

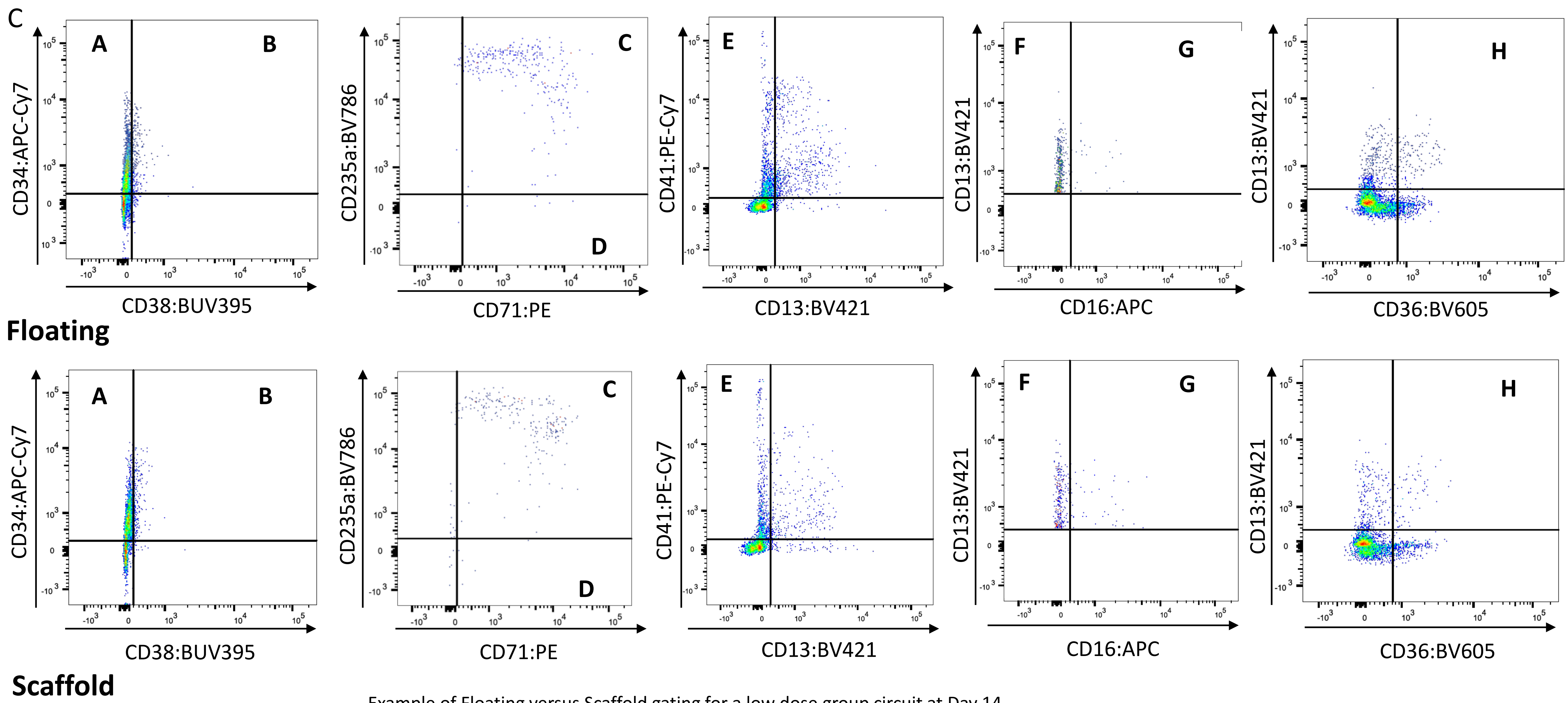

Example of Floating versus Scaffold gating for a low dose group circuit at Day 14.

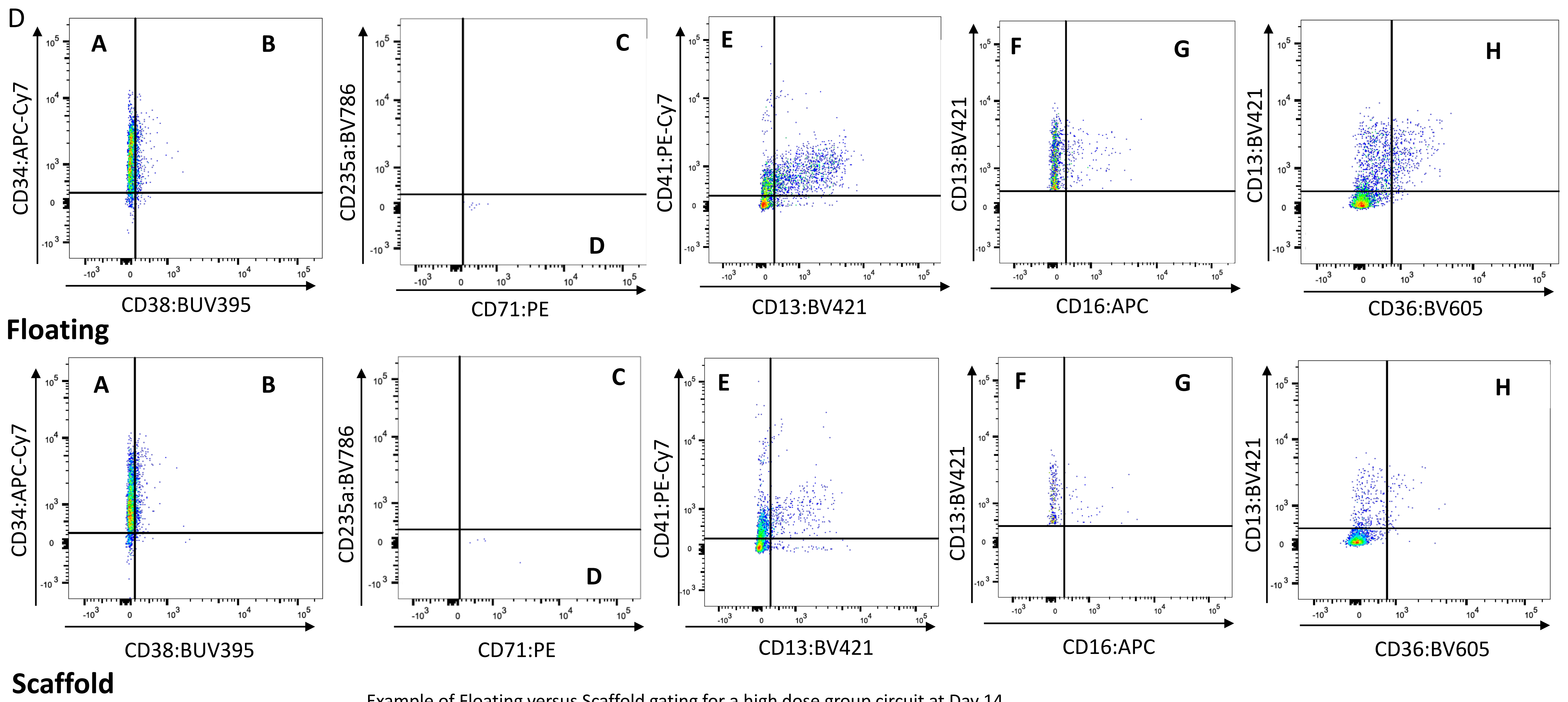

Example of Floating versus Scaffold gating for a high dose group circuit at Day 14.

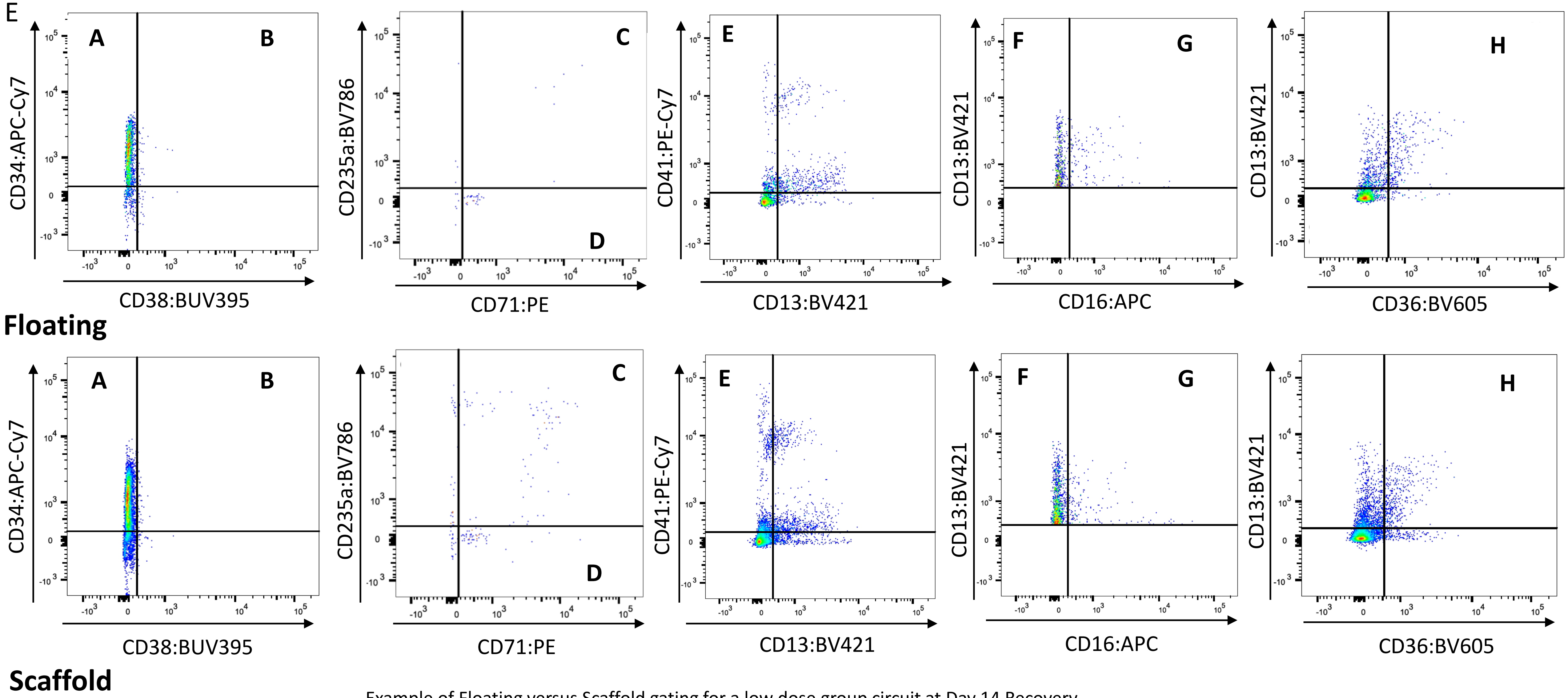

Example of Floating versus Scaffold gating for a low dose group circuit at Day 14 Recovery.

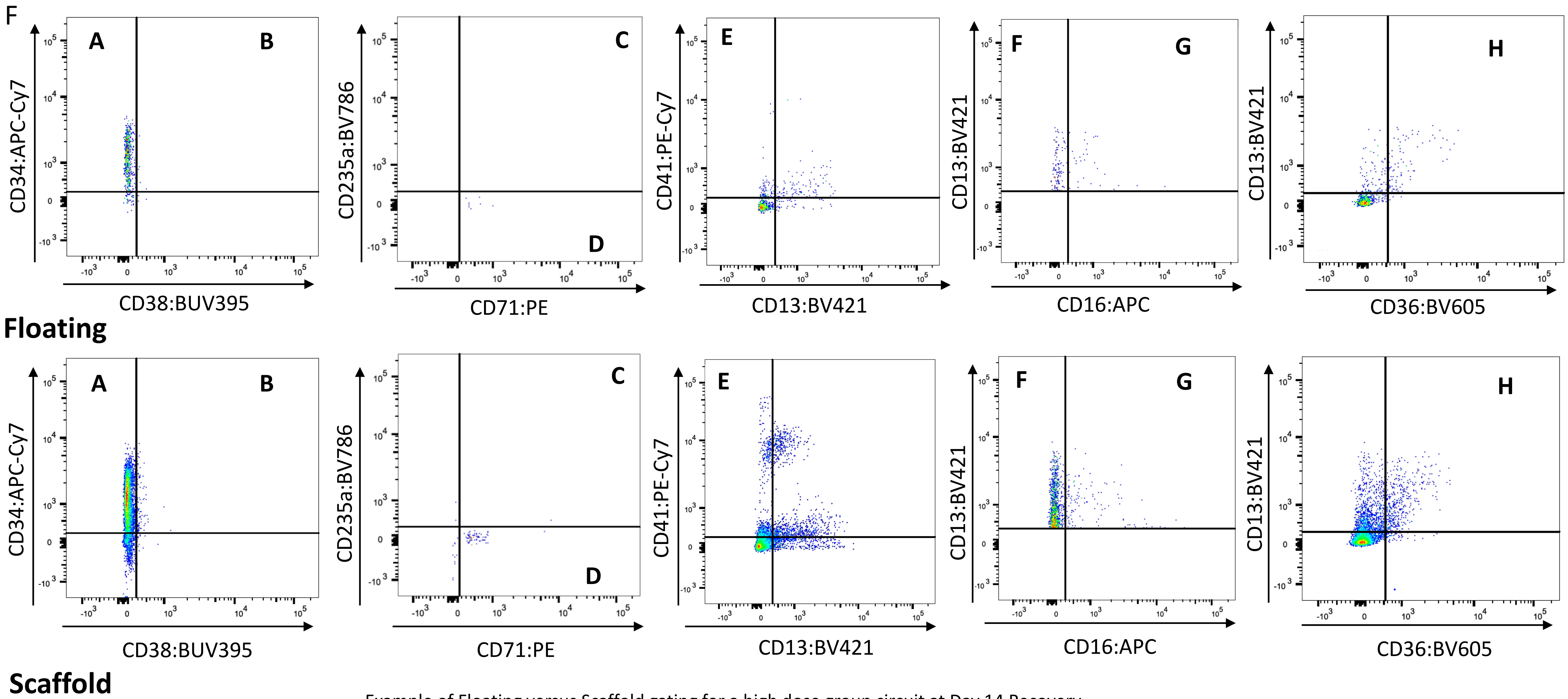

Example of Floating versus Scaffold gating for a high dose group circuit at Day 14 Recovery.

Supplementary Figure 4

(a)

Dead Cell %

	1 Floating	1 Scaffold	2 Floating	3 Floating
Operator	0.067 .	0.835	0.074 .	0.118
Side	0.216	0.863	0.770	0.089
Control Unit within incubator	0.126	0.485	0.093 .	0.877
Incubator	0.028 *	0.061 .	0.020 *	0.000613 ***

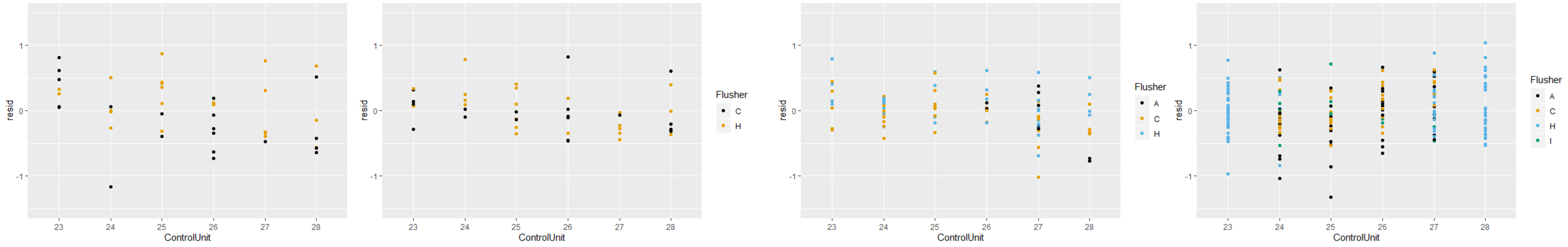

(b)

Late Erythroid %

	1 Floating	1 Scaffold	2 Floating	3 Floating
Operator	0.254	0.365	0.316	0.844
Side	0.493	0.840	0.885	0.230 *
Control Unit within incubator	0.759	0.787	2.25×10^{-10} ***	1.78×10^{-6} ***
Incubator	0.154	0.692	X	X
Control Unit	X	X	1.58×10^{-10} ***	3.53×10^{-6} ***

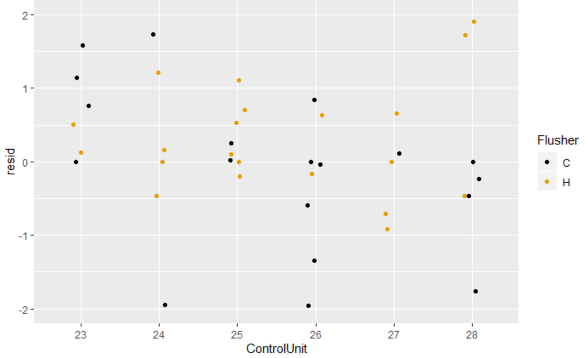

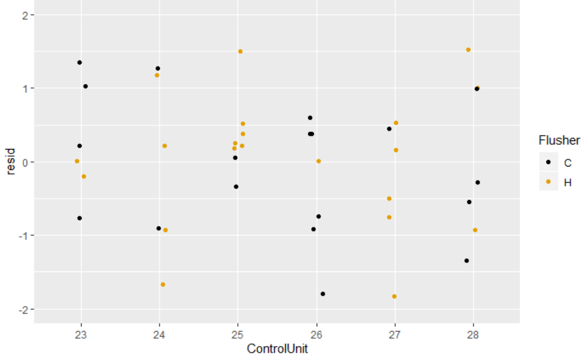

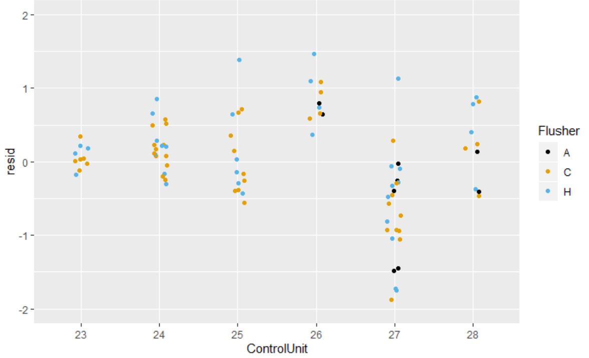

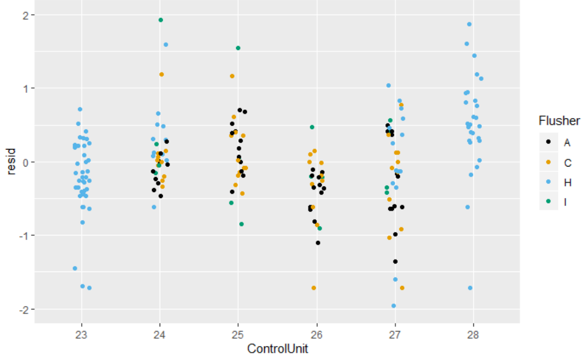

Supplementary Figure 5

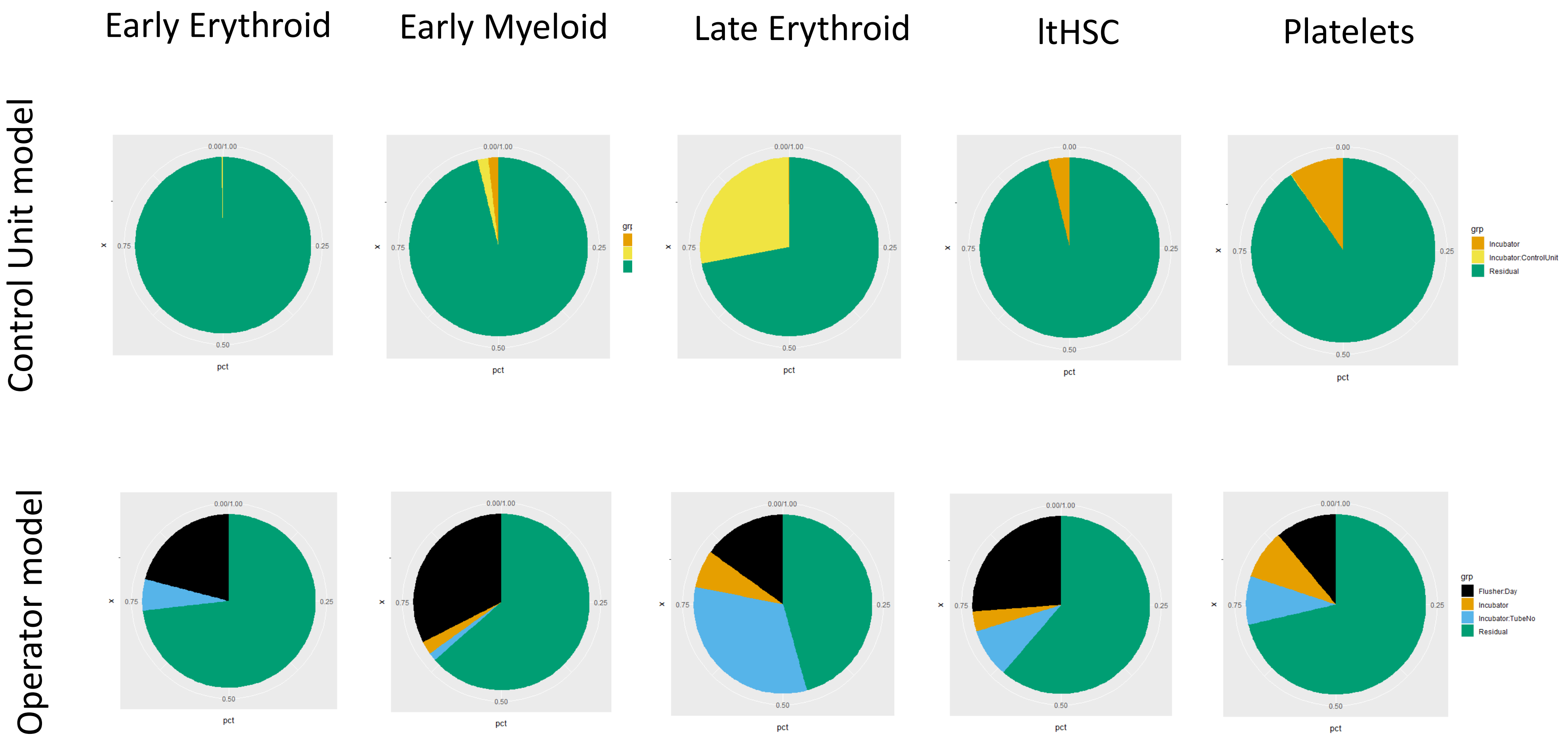

BM-3 Floating Counts

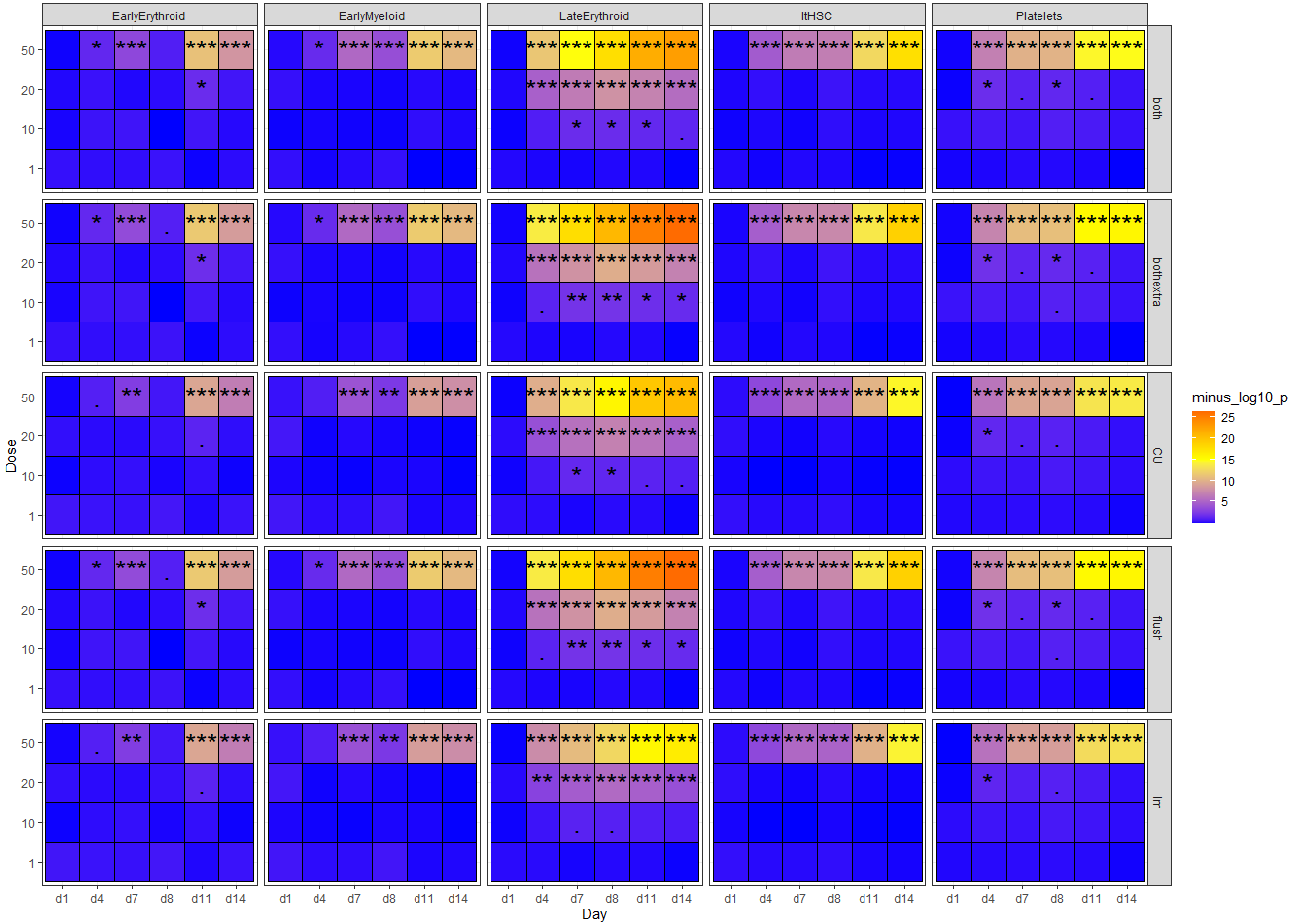
EarlyErythroid
EarlyMyeloid
LateErythroid
ltHSC
Platelets
both
bothextra
CU
flush
lm
Dose
50
20
10
1
Day
d1
d4
d7
d8
d11
d14
minus_log10_p
25
20
15
10
5

(data used in Supplementary fig 5)

EarlyErythroid

Condition	Dose	d1	d4	d7	d8	d11	d14
both	50	0.877	0.0372	0.000572	0.109	8.81e-12	5.92e-09
both	20	0.648	0.329	0.665	0.516	0.0208	0.244
both	10	0.787	0.298	0.27	0.999	0.243	0.596
both	1	0.394	0.454	0.439	0.319	0.901	0.506
bothextra	50	0.866	0.0358	0.00036	0.0944	2.94e-12	2.24e-09
bothextra	20	0.641	0.306	0.656	0.504	0.0162	0.223
bothextra	10	0.772	0.265	0.25	0.996	0.223	0.576
bothextra	1	0.38	0.442	0.424	0.304	0.902	0.495
CU	50	0.816	0.0932	0.00234	0.227	5.31e-10	2.35e-07
CU	20	0.421	0.531	0.544	0.365	0.059	0.463
CU	10	0.902	0.484	0.404	0.764	0.419	0.891
CU	1	0.24	0.322	0.355	0.218	0.687	0.326
flush	50	0.866	0.0358	0.00036	0.0944	2.94e-12	2.24e-09
flush	20	0.641	0.306	0.656	0.504	0.0162	0.223
flush	10	0.772	0.265	0.25	0.996	0.223	0.576
flush	1	0.38	0.442	0.424	0.304	0.902	0.495
lm	50	0.816	0.0944	0.00236	0.228	5.26e-10	2.36e-07
lm	20	0.422	0.532	0.545	0.365	0.0594	0.464
lm	10	0.902	0.488	0.404	0.764	0.42	0.892
lm	1	0.241	0.323	0.356	0.219	0.687	0.326

EarlyMyeloid

Condition	Dose	d1	d4	d7	d8	d11	d14
both	50	0.603	0.0253	4.9e-06	0.000213	1.92e-12	2.84e-11
both	20	0.38	0.737	0.753	0.83	0.574	0.724
both	10	0.898	0.791	0.861	0.887	0.452	0.703
both	1	0.416	0.804	0.584	0.4	0.988	0.979
bothextra	50	0.6	0.0255	4.38e-06	0.000192	2.13e-12	2.95e-11
bothextra	20	0.376	0.732	0.751	0.827	0.57	0.72
bothextra	10	0.896	0.786	0.859	0.884	0.446	0.699
bothextra	1	0.411	0.804	0.58	0.395	0.987	0.98
CU	50	0.349	0.107	0.000113	0.00399	1.33e-09	1.44e-08
CU	20	0.21	0.873	0.599	0.797	0.881	0.941
CU	10	0.719	0.883	0.927	0.748	0.75	0.962
CU	1	0.232	0.497	0.463	0.232	0.736	0.681
flush	50	0.6	0.0255	4.38e-06	0.000192	2.13e-12	2.95e-11
flush	20	0.376	0.732	0.751	0.827	0.57	0.72
flush	10	0.896	0.786	0.859	0.884	0.446	0.699
flush	1	0.411	0.804	0.58	0.395	0.987	0.98
lm	50	0.356	0.113	0.000138	0.00451	2.03e-09	2.11e-08
lm	20	0.217	0.875	0.604	0.8	0.883	0.942
lm	10	0.723	0.873	0.928	0.751	0.753	0.962
lm	1	0.238	0.503	0.469	0.239	0.74	0.685

LateErythroid

Condition	Dose	d1	d4	d7	d8	d11	d14
both	50	0.868	4.06e-12	1.2e-15	2.4e-18	2.51e-22	1.8e-23
both	20	0.676	1.65e-05	2.89e-07	1.05e-08	9.86e-08	2.36e-06
both	10	0.908	0.111	0.0207	0.0206	0.0346	0.0508
both	1	0.637	0.728	0.791	0.649	0.686	0.963
bothextra	50	0.832	1.54e-14	1.82e-18	1.76e-21	8.6e-26	5.14e-27
bothextra	20	0.651	1.15e-06	9.38e-09	1.71e-10	2.48e-09	1.13e-07
bothextra	10	0.878	0.0623	0.00859	0.00835	0.0159	0.0256
bothextra	1	0.606	0.711	0.766	0.614	0.654	0.946
CU	50	0.909	1.51e-10	2.84e-14	1.66e-16	2.83e-20	2.66e-21
CU	20	0.506	0.000178	1.46e-06	1.22e-07	8.84e-07	1.64e-05
CU	10	0.871	0.205	0.0332	0.0431	0.0661	0.0958
CU	1	0.474	0.493	0.757	0.55	0.587	0.89
flush	50	0.833	1.73e-14	1.84e-18	1.78e-21	8.73e-26	5.22e-27
flush	20	0.65	1.15e-06	9.42e-09	1.72e-10	2.49e-09	1.13e-07
flush	10	0.878	0.0634	0.0086	0.00836	0.0159	0.0257
flush	1	0.606	0.712	0.766	0.614	0.654	0.946
lm	50	0.922	2.58e-08	2.17e-11	3.28e-13	2.48e-16	3.41e-17
lm	20	0.567	0.00117	2.85e-05	4.13e-06	1.93e-05	0.000187
lm	10	0.889	0.29	0.0664	0.0812	0.113	0.151
lm	1	0.537	0.555	0.79	0.607	0.64	0.905

ltHSC

Condition	Dose	d1	d4	d7	d8	d11	d14
both	50	0.763	3.71e-05	2.49e-07	1.87e-07	2.96e-13	2.52e-18
both	20	0.709	0.415	0.711	0.298	0.571	0.58
both	10	0.883	0.704	0.864	0.465	0.714	0.676
both	1	0.64	0.855	0.655	0.682	0.955	0.935
bothextra	50	0.757	2.39e-05	5.45e-08	3.88e-08	2.7e-14	1.58e-19
bothextra	20	0.7	0.374	0.69	0.263	0.541	0.549
bothextra	10	0.866	0.662	0.852	0.43	0.691	0.649
bothextra	1	0.627	0.862	0.637	0.67	0.958	0.923
CU	50	0.499	0.000541	4.19e-06	8.77e-06	6.35e-11	3e-15
CU	20	0.458	0.739	0.853	0.568	0.806	0.861
CU	10	0.789	0.992	0.997	0.773	0.95	0.958
CU	1	0.408	0.564	0.576	0.453	0.747	0.8
flush	50	0.757	2.39e-05	5.45e-08	3.88e-08	2.7e-14	1.58e-19
flush	20	0.7	0.374	0.69	0.263	0.541	0.549
flush	10	0.866	0.662	0.852	0.43	0.691	0.649
flush	1	0.627	0.862	0.637	0.67	0.958	0.923
lm	50	0.504	0.000561	5.31e-06	1.1e-05	9.82e-11	5.41e-15
lm	20	0.464	0.742	0.855	0.572	0.808	0.862
lm	10	0.791	0.976	0.997	0.776	0.95	0.958
lm	1	0.413	0.569	0.58	0.458	0.75	0.802

Platelets

Condition	Dose	d1	d4	d7	d8	d11	d14
both	50	0.852	1.54e-07	9.36e-11	6.85e-11	3.19e-15	1.63e-15
both	20	0.915	0.0185	0.0665	0.0413	0.0924	0.308
both	10	0.354	0.189	0.249	0.106	0.163	0.365
both	1	0.818	0.709	0.548	0.619	0.712	0.976
bothextra	50	0.831	7.22e-08	1.69e-11	1.17e-11	3.87e-16	1.98e-16
bothextra	20	0.897	0.0124	0.0524	0.0305	0.0737	0.276
bothextra	10	0.32	0.153	0.222	0.0857	0.138	0.333
bothextra	1	0.821	0.711	0.531	0.61	0.708	0.984
CU	50	0.97	8.06e-07	4.8e-10	6.38e-10	4.98e-14	2.09e-14
CU	20	0.909	0.0403	0.0915	0.0749	0.153	0.409
CU	10	0.497	0.274	0.304	0.171	0.25	0.475
CU	1	0.66	0.552	0.504	0.499	0.578	0.848
flush	50	0.83	7.22e-08	1.69e-11	1.17e-11	3.87e-16	1.98e-16
flush	20	0.897	0.0124	0.0524	0.0305	0.0737	0.276
flush	10	0.32	0.153	0.222	0.0857	0.138	0.333
flush	1	0.821	0.711	0.531	0.61	0.708	0.984
lm	50	0.971	1.25e-06	1.31e-09	1.72e-09	1.92e-13	8.28e-14
lm	20	0.912	0.0467	0.102	0.0839	0.165	0.423
lm	10	0.51	0.274	0.319	0.184	0.265	0.488
lm	1	0.669	0.564	0.517	0.512	0.589	0.853

Day

minus_log10_p: 25, 20, 15, 10, 5

Supplementary Figure 6

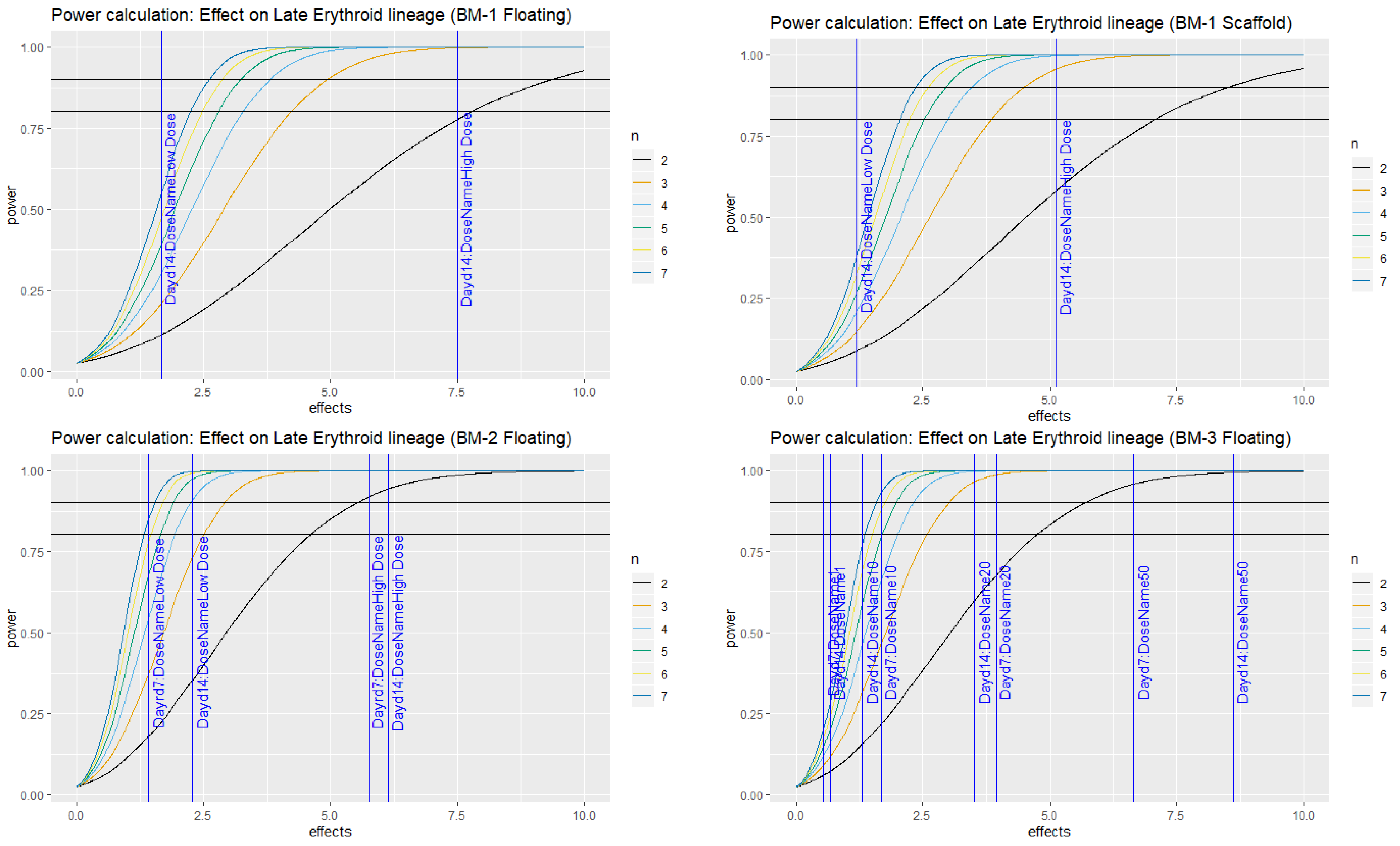

Power calculation: Effect on Late Erythroid lineage (BM-1 Floating)
Dayd14:DoseNameLow Dose
Dayd14:DoseNameHigh Dose
power
effects
1.00
0.75
0.50
0.25
0.00
0.0
2.5
5.0
7.5
10.0
n
2
3
4
5
6
7
Power calculation: Effect on Late Erythroid lineage (BM-1 Scaffold)
Dayd14:DoseNameLow Dose
Dayd14:DoseNameHigh Dose
power
effects
Power calculation: Effect on Late Erythroid lineage (BM-2 Floating)
Dayrd7:DoseNameLow Dose
Dayd14:DoseNameLow Dose
Dayrd7:DoseNameHigh Dose
Dayd14:DoseNameHigh Dose
power
effects
Power calculation: Effect on Late Erythroid lineage (BM-3 Floating)
Dayd14:DoseName10
Dayd7:DoseName10
Dayd14:DoseName20
Dayd7:DoseName20
Dayd7:DoseName50
Dayd14:DoseName50
power
effects

Supplementary Figure 7

Top Incubator	Circuit		Operator
	LHS	RHS	
Treatment	A	G	1
Circuit #	1	2	
Control Unit	23	23	
Treatment	C	D	1
Circuit #	3	4	
Control Unit	23	23	
Treatment	F	E	1
Circuit #	5	6	
Control Unit	23	23	
Treatment	B	H	1
Circuit #	7	8	
Control Unit	23	23	
Treatment	D	B	1
Circuit #	9	10	
Control Unit	24	24	
Treatment	H	A	1
Circuit #	11	12	
Control Unit	24	24	
Treatment	G	C	1
Circuit #	13	14	
Control Unit	24	24	
Treatment	E	F	1
Circuit #	15	16	
Control Unit	24	24	
Treatment	C	F	2
Circuit #	17	18	
Control Unit	25	25	
Treatment	E	B	2
Circuit #	19	20	
Control Unit	25	25	
Treatment	H	D	2
Circuit #	21	22	
Control Unit	25	25	
Treatment	A	G	2
Circuit #	23	24	
Control Unit	25	25	

Bottom Incubator	Circuit		Operator
	LHS	RHS	
Treatment	G	A	1
Circuit #	25	26	
Control Unit	26	26	
Treatment	D	H	1
Circuit #	27	28	
Control Unit	26	26	
Treatment	B	E	1
Circuit #	29	30	
Control Unit	26	26	
Treatment	F	C	1
Circuit #	31	32	
Control Unit	26	26	
Treatment	F	E	1
Circuit #	33	34	
Control Unit	27	27	
Treatment	C	G	1
Circuit #	35	36	
Control Unit	27	27	
Treatment	A	H	1
Circuit #	37	38	
Control Unit	27	27	
Treatment	B	D	1
Circuit #	39	40	
Control Unit	27	27	
Treatment	H	B	2
Circuit #	41	42	
Control Unit	28	28	
Treatment	E	F	2
Circuit #	43	44	
Control Unit	28	28	
Treatment	D	C	2
Circuit #	45	46	
Control Unit	28	28	
Treatment	G	A	2
Circuit #	47	48	
Control Unit	28	28	
