# Supplementary material for: Optimal experimental design for efficient toxicity testing in microphysiological systems: A bone marrow application
Source: Front Pharmacol. 2023 Mar 31;14:1142581. doi: 10.3389/fphar.2023.1142581 (PMC10103791; doi:10.3389/fphar.2023.1142581)
Supplement: Supplementary file 2 [file DataSheet1.zip › Data package/Reports/compare-PAPER.html]

Bone Marrow MPS - compare BM-1 and -2


Code 

- Show All Code
- Hide All Code

# Bone Marrow MPS - compare BM-1 and -2

#### Statistician: Jonathan Cairns

#### 5 April 2019

Read in data:

```
source("createFull.R")

## create full_gathered
interesting_cols <- c(
  "EarlyErythroid", "LateErythroid", "EarlyMyeloid", "ltHSC",
  "Platelets", "LineageDiffd", "LateGranulocytes"
)
metadata_cols <- c(
  colnames(full)[1:10],
  "Study", "Group", "Day", "StudyFraction"
)

## specify the columns of interest
mySel <- c(
  colnames(full) %>% grep(pattern = "CD"),
  colnames(full) %>% grep(pattern = "DeadCells_pct"),
  which(colnames(full) %in% c(
    interesting_cols,
    paste0(interesting_cols, "_pct")
  ))
)

full_gathered <- full %>% gather_(
  key_col      = "Parameter",
  value_col    = "value",
  gather_cols  = colnames(full)[mySel]
)
```

```
## Warning: `gather_()` was deprecated in tidyr 1.2.0.
## ℹ Please use `gather()` instead.
```

```
temp_cols <- c(
  metadata_cols,
  colnames(full) %>% grep(pattern = "CD", value = TRUE),
  paste0(c(interesting_cols, "DeadCells"), "_pct")
)
for_PCA <- as.data.frame(full)[, temp_cols]
```

# PCA

PCA is given for PCs 1 through 4 for all subsets of the data that
were explored. Loadings are presented on the plot.

## PCA 1) BM-1, BM-2 and BM-3

```
## keep only %ages, provided there's no missing data


sel <- grepl("_pct", colnames(for_PCA)) &
  !apply(for_PCA, MARGIN = 2, . %>% is.na() %>% any(.))

setDT(for_PCA)
temp <- for_PCA[, sel, with = FALSE]

# Perform PCA
pca <- prcomp(temp, scale. = TRUE)
plot(pca)
```

```
ggbiplot(pca, groups = for_PCA$Study) +
  scale_colour_manual(values = cbbPalette)
```

```
ggbiplot(pca, groups = for_PCA$Study, choices = 3:4) +
  scale_colour_manual(values = cbbPalette)
```

```
ggbiplot(pca, groups = for_PCA$Day, alpha = 0) +
  scale_colour_manual(values = rainbow(16)) +
  scale_shape_manual(values = c(3, 16, 17)) +
  geom_point(aes(shape = for_PCA$Study, colour = for_PCA$Day))
```

```
ggbiplot(pca, groups = for_PCA$DoseName, alpha = 0) +
  scale_colour_manual(values = cbbPalette) +
  scale_shape_manual(values = 1:16) +
  geom_point(aes(shape = for_PCA$Day, colour = for_PCA$DoseName))
```

```
ggbiplot(pca, groups = for_PCA$DoseName, alpha = 0) +
  scale_colour_manual(values = cbbPalette) +
  scale_shape_manual(values = 1:16) +
  geom_point(aes(shape = for_PCA$Day, colour = for_PCA$DoseName))
```

```
temp_D <- cbind(for_PCA, as.data.frame(pca$x))
temp_D <- temp_D[temp_D$Fraction == "Floating", ]

panel_D <- ggplot(temp_D,
  aes(
    x = PC1, y = PC2, shape = temp_D$Day,
    colour = temp_D$DoseName
  ),
  groups = temp_D$DoseName
) +
  scale_colour_manual(values = cbbPalette) +
  scale_shape_manual(values = 1:16) + ## was c(1, 2, 3, 16, 17)
  theme_bw() +
  guides(
    shape = guide_legend(title = "Day"),
    colour = guide_legend(title = "Dose")
  ) +
  geom_point(size = 2.5) +
  facet_grid(Fraction ~ Study)
print(panel_D)
```

```
ggplot(temp_D, aes(
  x = PC2, y = PC3, shape = temp_D$Day,
  colour = temp_D$DoseName
), groups = temp_D$DoseName) +
  scale_colour_manual(values = cbbPalette) +
  scale_shape_manual(values = 1:16) +
  theme_bw() +
  guides(
    shape = guide_legend(title = "Day"),
    colour = guide_legend(title = "Dose")
  ) +
  geom_point() +
  facet_grid(Fraction ~ Study)
```

## PCA 2) BM-1, BM-2 and control data from BM-3

Here’s an attempt with only the control of BM-3 on the grounds that
we wouldn’t expect the dose to be similar.

```
## keep only %ages, provided there's no missing data
setDT(for_PCA)

## subset down to only control data in 2018-08, and only on the same days
toDrop <- for_PCA[, Time %in% c(1, 4, 8, 11)]
toDrop2 <- for_PCA[, Study == "BM-3" & !(Dose == 0)]
for_PCA_simple <- for_PCA[!(toDrop) & !(toDrop2), ]

sel <- grepl("_pct", colnames(for_PCA)) &
  !apply(for_PCA, MARGIN = 2, . %>% is.na() %>% any(.))
temp_simple <- for_PCA_simple[, sel, with = FALSE]

# Perform PCA
pca <- prcomp(temp_simple, scale. = TRUE)
plot(pca)
```

```
ggbiplot(pca, groups = for_PCA_simple$Study) +
  scale_colour_manual(values = cbbPalette)
```

```
ggbiplot(pca, groups = for_PCA_simple$Study, choices = 3:4) +
  scale_colour_manual(values = cbbPalette)
```

```
ggbiplot(pca, groups = for_PCA_simple$Day, alpha = 0) +
  scale_colour_manual(values = cbbPalette) +
  scale_shape_manual(values = c(3, 16, 17)) +
  geom_point(aes(shape = for_PCA_simple$Study, colour = for_PCA_simple$Day))
```

```
ggbiplot(pca, groups = for_PCA_simple$DoseName, alpha = 0) +
  scale_colour_manual(values = cbbPalette) +
  scale_shape_manual(values = 1:16) +
  geom_point(aes(shape = for_PCA_simple$Day, colour = for_PCA_simple$DoseName))
```

```
ggbiplot(pca, groups = for_PCA_simple$DoseName, alpha = 0) +
  scale_colour_manual(values = cbbPalette) +
  scale_shape_manual(values = 1:16) +
  geom_point(aes(shape = for_PCA_simple$Day, colour = for_PCA_simple$DoseName))
```

```
temp_C <- cbind(for_PCA_simple, as.data.frame(pca$x))
temp_C <- temp_C[temp_C$Fraction == "Floating", ]

panel_C <- ggplot(temp_C,
  aes(
    x = PC1, y = PC2, shape = temp_C$Day,
    colour = temp_C$DoseName
  ),
  groups = temp_C$DoseName
) +
  scale_colour_manual(values = cbbPalette) +
  scale_shape_manual(values = 1:16) +
  geom_point(size = 2.5) +
  theme_bw() +
  guides(
    shape = guide_legend(title = "Day"),
    colour = guide_legend(title = "Dose")
  ) +
  facet_grid(Fraction ~ Study)

print(panel_C)
```

```
ggplot(temp_C,
  aes(
    x = PC2, y = PC3, shape = temp_C$Day,
    colour = temp_C$DoseName
  ),
  groups = temp_C$DoseName
) +
  scale_colour_manual(values = cbbPalette) +
  scale_shape_manual(values = 1:16) +
  geom_point() +
  theme_bw() +
  guides(
    shape = guide_legend(title = "Day"),
    colour = guide_legend(title = "Dose")
  ) +
  facet_grid(Fraction ~ Study)
```

## PCA 3) BM-1 and BM-2

PCA only on BM-1 and BM-2.

```
## keep only %ages, provided there's no missing data
setDT(for_PCA)

## subset down to only control data in 2018-08, and only on the same days
for_PCA_simple <- for_PCA[Study != "BM-3", ]

sel <- grepl("_pct", colnames(for_PCA)) &
  !apply(for_PCA, MARGIN = 2, . %>% is.na() %>% any(.))
temp_simple <- for_PCA_simple[, sel, with = FALSE]

# Perform PCA
pca <- prcomp(temp_simple, scale. = TRUE)
plot(pca)
```

```
ggbiplot(pca, groups = for_PCA_simple$Study) +
  scale_colour_manual(values = cbbPalette)
```

```
ggbiplot(pca, groups = for_PCA_simple$Study, choices = 3:4) +
  scale_colour_manual(values = cbbPalette)
```

```
ggbiplot(pca, groups = for_PCA_simple$Day, alpha = 0) +
  scale_colour_manual(values = cbbPalette) +
  scale_shape_manual(values = c(3, 16, 17)) +
  geom_point(aes(shape = for_PCA_simple$Study, colour = for_PCA_simple$Day))
```

```
ggbiplot(pca, groups = for_PCA_simple$DoseName, alpha = 0) +
  scale_colour_manual(values = cbbPalette) +
  scale_shape_manual(values = 1:16) +
  geom_point(aes(shape = for_PCA_simple$Day, colour = for_PCA_simple$DoseName))
```

```
ggbiplot(pca, groups = for_PCA_simple$DoseName, alpha = 0) +
  scale_colour_manual(values = cbbPalette) +
  scale_shape_manual(values = 1:16) +
  geom_point(aes(shape = for_PCA_simple$Day, colour = for_PCA_simple$DoseName))
```

```
temp <- cbind(for_PCA_simple, as.data.frame(pca$x))
temp <- temp[temp$Fraction == "Floating", ]

ggplot(temp,
  aes(x = PC1, y = PC2, shape = temp$Day, colour = temp$DoseName),
  groups = temp$DoseName
) +
  scale_colour_manual(values = cbbPalette) +
  scale_shape_manual(values = 1:16) +
  geom_point() +
  facet_grid(Fraction ~ Study)
```

```
ggplot(temp,
  aes(x = PC2, y = PC3, shape = temp$Day, colour = temp$DoseName),
  groups = temp$DoseName
) +
  scale_colour_manual(values = cbbPalette) +
  scale_shape_manual(values = 1:16) +
  geom_point() +
  facet_grid(Fraction ~ Study)
```

```
ggplot(temp,
  aes(x = PC3, y = PC4, shape = temp$Day, colour = temp$DoseName),
  groups = temp$DoseName
) +
  scale_colour_manual(values = cbbPalette) +
  scale_shape_manual(values = 1:16) +
  geom_point() +
  facet_grid(Fraction ~ Study)
```

Same but with floating and scaffold

```
temp2 <- cbind(for_PCA_simple, as.data.frame(pca$x))

panel_A <- ggplot(temp2,
  aes(x = PC1, y = PC2, shape = temp2$Day, colour = temp2$DoseName),
  groups = temp2$DoseName
) +
  scale_colour_manual(values = cbbPalette) +
  scale_shape_manual(values = 1:16) +
  geom_point(size = 2.5) +
  theme_bw() +
  guides(
    shape = guide_legend(title = "Day"),
    colour = guide_legend(title = "Dose")
  ) +
  facet_grid(Fraction ~ Study)

print(panel_A)
```

```
panel_B <- ggplot(temp2,
  aes(x = PC3, y = PC4, shape = temp2$Day, colour = temp2$DoseName),
  groups = temp2$DoseName
) +
  scale_colour_manual(values = cbbPalette) +
  scale_shape_manual(values = 1:16) +
  geom_point(size = 2.5) +
  theme_bw() +
  guides(
    shape = guide_legend(title = "Day"),
    colour = guide_legend(title = "Dose")
  ) +
  facet_grid(Fraction ~ Study)

print(panel_B)
```

## PCA 4) Summary figure for paper

```
meta_1 <- panel_A / panel_B +
  plot_layout(byrow = FALSE, widths = c(1, 2), guides = "collect")
meta_2 <- panel_C / panel_D +
  plot_layout(byrow = FALSE, widths = c(1, 2))
(meta_1 | meta_2) + plot_layout(byrow = FALSE, widths = c(1, 2)) +
  plot_annotation(tag_levels = "A") & theme(text = element_text(size = 25))
```

# Endpoints

Plot endpoints of interest (percent)

```
temp_pars <- c(
  "ltHSC_pct", "EarlyErythroid_pct", "LateErythroid_pct",
  "Platelets_pct", "EarlyMyeloid_pct"
)

forPlotting <- full_gathered %>% filter(Parameter %in% temp_pars)
forPlotting$Parameter <- factor(forPlotting$Parameter, levels = temp_pars)

forPlotting$val.vst <- sqrt(forPlotting$value / 100)

## visualise transformed values
p6 <- ggplot(
  forPlotting[forPlotting$StudyFraction == "BM-2 Floating", ],
  aes_string(
    x = "Time",
    y = "val.vst",
    colour = "DoseName",
    group = "TubeNo"
  )
) +
  geom_point() +
  geom_line() +
  scale_colour_manual(values = cbbPalette) +
  ylab("Percent of cells with marker")
```

```
## Warning: `aes_string()` was deprecated in ggplot2 3.0.0.
## ℹ Please use tidy evaluation ideoms with `aes()`
```

```
p6 <- p6 + facet_wrap("Parameter ~ .")
print(p6)
```

```
p6 <- ggplot(
  forPlotting[forPlotting$StudyFraction == "BM-3 Floating", ],
  aes_string(
    x = "Time",
    y = "val.vst",
    colour = "DoseName",
    group = "TubeNo"
  )
) +
  geom_point() +
  geom_line() +
  scale_colour_manual(values = cbbPalette) +
  ylab("Percent of cells with marker")
p6 <- p6 + facet_wrap("Parameter ~ .")
print(p6)
```

```
temp <- lapply(
  unique(forPlotting$Parameter),
  function(endpoint) {
    out.fixed <- lm(val.vst ~ Time + Time:DoseName,
      data = forPlotting,
      subset = forPlotting$Parameter == endpoint
    )
    summary(out.fixed)

    ## visualise predicted values from models
    myPredict <- as.data.frame(
      predict(out.fixed, interval = "predict", se.fit = TRUE)
    )
    myPredict$upperSE <- myPredict$fit.fit + 2 * myPredict$se.fit
    myPredict$lowerSE <- myPredict$fit.fit - 2 * myPredict$se.fit
    myPredict
  }
)
```

```
## Warning in predict.lm(out.fixed, interval = "predict", se.fit = TRUE): predictions on current data refer to _future_ responses
```

```
## Warning in predict.lm(out.fixed, interval = "predict", se.fit = TRUE): predictions on current data refer to _future_ responses

## Warning in predict.lm(out.fixed, interval = "predict", se.fit = TRUE): predictions on current data refer to _future_ responses

## Warning in predict.lm(out.fixed, interval = "predict", se.fit = TRUE): predictions on current data refer to _future_ responses

## Warning in predict.lm(out.fixed, interval = "predict", se.fit = TRUE): predictions on current data refer to _future_ responses
```

```
forPlotting <- cbind(forPlotting, do.call(rbind, temp))

toPlot <- unique(forPlotting[, c(
  "Fraction", "Day", "Study", "fit.fit",
  "DoseName", "upperSE", "lowerSE",
  "Parameter", "StudyFraction"
)])

sel <- duplicated(toPlot[, c(
  "Fraction", "Day", "Study", "fit.fit",
  "DoseName", "Parameter"
)])
toPlot <- toPlot[!sel, ]
## Some duplicates remain due to a weird machine precision effect!!


## copy first day to different doses
toAdd <- rbind(
  toPlot[toPlot$Day == "d0" & toPlot$Study == "BM-1", ] %>%
    mutate(DoseName = "AZ1 Low"),
  toPlot[toPlot$Day == "d0" & toPlot$Study == "BM-1", ] %>%
    mutate(DoseName = "AZ1 High")
)
toPlot <- rbind(toPlot, toAdd)

p7 <- ggplot(
  toPlot[toPlot$Study != "BM-3" & toPlot$StudyFraction != "BM-2 Scaffold", ],
  aes_string(
    x = "Day",
    y = "fit.fit",
    colour = "DoseName",
    fill = "DoseName",
    group = "DoseName",
    ymax = "upperSE",
    ymin = "lowerSE"
  )
) +
  geom_point() +
  geom_line() +
  geom_ribbon(alpha = 0.3) +
  scale_colour_manual(values = cbbPalette[-1]) +
  scale_fill_manual(values = cbbPalette[-1]) +
  ylab("sqrt(Percent of cells with marker)") +
  facet_grid(StudyFraction ~ Parameter) +
  theme_bw()
p7 <- p7
print(p7)
```

```
p8 <- ggplot(
  toPlot[toPlot$Study == "BM-3" & toPlot$Fraction == "Floating", ],
  aes_string(
    x = "Day",
    y = "fit.fit",
    colour = "DoseName",
    fill = "DoseName",
    group = "DoseName",
    ymax = "upperSE",
    ymin = "lowerSE"
  )
) +
  geom_point() +
  geom_line() +
  geom_ribbon(alpha = 0.3) +
  scale_colour_manual(values = cbbPalette[-1]) +
  scale_fill_manual(values = cbbPalette[-1]) +
  ylab("sqrt(Percent of cells with marker)") +
  facet_grid(Study ~ Parameter) +
  theme_bw()
p8 <- p8
print(p8)
```

Plot endpoints of interest (count)

```
temp_pars <- c(
  "ltHSC", "EarlyErythroid", "LateErythroid", "Platelets",
  "EarlyMyeloid"
)

forPlotting <- full_gathered %>%
  filter(Parameter %in% temp_pars)
forPlotting$Parameter <- factor(forPlotting$Parameter, levels = temp_pars)

# Cap away 0s and round off
forPlotting$value[!is.finite(forPlotting$value)] <- min(
  forPlotting$value[is.finite(forPlotting$value)]
)
forPlotting$value[forPlotting$value < 0.01] <- 1
forPlotting$value <- round(forPlotting$value)

## visualise transformed values
p6 <- ggplot(
  forPlotting[forPlotting$StudyFraction == "BM-1 Scaffold", ],
  aes_string(
    x = "Time",
    y = "value",
    colour = "DoseName",
    group = "TubeNo"
  )
) +
  geom_point() +
  geom_line() +
  theme_bw() +
  scale_colour_manual(values = cbbPalette) +
  scale_y_continuous(trans = "log10") +
  ylab("Number of cells")
p6 <- p6 + facet_grid("StudyFraction ~ Parameter")
print(p6)
```

```
## `geom_line()`: Each group consists of only one observation.
## ℹ Do you need to adjust the group aesthetic?
## `geom_line()`: Each group consists of only one observation.
## ℹ Do you need to adjust the group aesthetic?
## `geom_line()`: Each group consists of only one observation.
## ℹ Do you need to adjust the group aesthetic?
## `geom_line()`: Each group consists of only one observation.
## ℹ Do you need to adjust the group aesthetic?
## `geom_line()`: Each group consists of only one observation.
## ℹ Do you need to adjust the group aesthetic?
```

```
## visualise transformed values
p6 <- ggplot(
  forPlotting[forPlotting$StudyFraction == "BM-1 Floating", ],
  aes_string(
    x = "Time",
    y = "value",
    colour = "DoseName",
    group = "TubeNo"
  )
) +
  geom_point() +
  geom_line() +
  theme_bw() +
  scale_colour_manual(values = cbbPalette) +
  scale_y_continuous(trans = "log10") +
  ylab("Number of cells")
p6 <- p6 + facet_grid("StudyFraction ~ Parameter")
print(p6)
```

```
## `geom_line()`: Each group consists of only one observation.
## ℹ Do you need to adjust the group aesthetic?
## `geom_line()`: Each group consists of only one observation.
## ℹ Do you need to adjust the group aesthetic?
## `geom_line()`: Each group consists of only one observation.
## ℹ Do you need to adjust the group aesthetic?
## `geom_line()`: Each group consists of only one observation.
## ℹ Do you need to adjust the group aesthetic?
## `geom_line()`: Each group consists of only one observation.
## ℹ Do you need to adjust the group aesthetic?
```

```
## visualise transformed values
p6 <- ggplot(
  forPlotting[forPlotting$StudyFraction == "BM-2 Floating", ],
  aes_string(
    x = "Time",
    y = "value",
    colour = "DoseName",
    group = "TubeNo"
  )
) +
  geom_point() +
  geom_line() +
  theme_bw() +
  scale_colour_manual(values = cbbPalette) +
  scale_y_continuous(trans = "log10") +
  ylab("Number of cells")
p6 <- p6 + facet_grid("StudyFraction ~ Parameter")
print(p6)
```

```
p6 <- ggplot(
  forPlotting[forPlotting$StudyFraction == "BM-3 Floating", ],
  aes_string(
    x = "Time",
    y = "value",
    colour = "DoseName",
    group = "TubeNo"
  )
) +
  geom_point() +
  geom_line() +
  theme_bw() +
  scale_colour_manual(values = cbbPalette) +
  scale_y_continuous(trans = "log10") +
  ylab("Number of cells")
p6 <- p6 + facet_grid("StudyFraction ~ Parameter")
print(p6)
```

```
temp <- lapply(
  unique(forPlotting$Parameter),
  function(endpoint) {
    out.fixed <- glm.nb(value ~ Time + Time:DoseName,
                        data = forPlotting,
                        subset = forPlotting$Parameter == endpoint)
    summary(out.fixed)

    ## visualise predicted values from models
    myPredict <- as.data.frame(predict(out.fixed, interval = "predict", se.fit = TRUE))
    myPredict$upperSE <- myPredict$fit + 2 * myPredict$se.fit
    myPredict$lowerSE <- myPredict$fit - 2 * myPredict$se.fit
    myPredict
  }
)
forPlotting <- cbind(forPlotting, do.call(rbind, temp))

temp_cols <- c("Fraction", "Day", "Study", "fit", "DoseName",
               "upperSE", "lowerSE", "Parameter", "StudyFraction")
toPlot <- unique(forPlotting[, temp_cols])

## This is not enough to remove all the duplicates because of a weird machine precision effect!!
sel <- duplicated(toPlot[, c("Fraction", "Day", "Study", "fit", "DoseName", "Parameter")])
toPlot <- toPlot[!sel, ]

## copy first day to different doses
toAdd <- rbind(
  toPlot[toPlot$Day == "d0" & toPlot$Study == "BM-1", ] %>% mutate(DoseName = "AZ1 Low"),
  toPlot[toPlot$Day == "d0" & toPlot$Study == "BM-1", ] %>% mutate(DoseName = "AZ1 High")
)
toPlot <- rbind(toPlot, toAdd)

p7 <- ggplot(toPlot[toPlot$Study != "BM-3" & toPlot$StudyFraction != "BM-2 Scaffold", ], aes_string(
  x = "Day",
  y = "fit",
  colour = "DoseName",
  fill = "DoseName",
  group = "DoseName",
  ymax = "upperSE",
  ymin = "lowerSE"
)) +
  geom_rect(aes(xmin = "d0", xmax = "d14", ymin = -Inf, ymax = Inf),
            fill = "grey92", colour = NA) +
  geom_point() +
  geom_line() +
  geom_ribbon(alpha = 0.3) +
  scale_colour_manual(values = cbbPalette[-1]) +
  scale_fill_manual(values = cbbPalette[-1]) +
  coord_cartesian(ylim = c(-1, 12.5)) +
  # scale_y_continuous(trans="log10") +
  ylab("log10(Number of cells with marker)") +
  facet_grid(StudyFraction ~ Parameter) +
  theme_bw()
p7 <- p7 #+ facet_grid("Parameter ~ DoseName")
print(p7)
```

```
## cheating to avoid the disappearing ribbon
# sel <- toPlot$lowerSE < 0
# toPlot$lowerSE[sel] <-

toPlot_p8 <- toPlot[toPlot$Study == "BM-3" & toPlot$Fraction == "Floating", ]

p8 <- ggplot(toPlot_p8, aes_string(
  x = "Day",
  y = "fit",
  colour = "DoseName",
  fill = "DoseName",
  group = "DoseName",
  ymax = "upperSE",
  ymin = "lowerSE",
  label = "Fraction"
)) +
  geom_rect(aes(xmin = "d0", xmax = "d1", ymin = -Inf, ymax = Inf),
    fill = "grey92", colour = NA
  ) +
  geom_point() +
  geom_line() +
  geom_ribbon(alpha = 0.3) +
  scale_colour_manual(values = cbbPalette[-1]) +
  scale_fill_manual(values = cbbPalette[-1]) +
  coord_cartesian(ylim = c(-1, 12.5)) +
  # scale_y_continuous(trans="log10") +
  ylab("log10(Number of cells with marker)") +
  facet_grid(Study ~ Parameter) +
  theme_bw()
p8 <- p8
print(p8)
```

Add significance stars

```
temp1 <- fread(here("Reports", "output_BM-1_lm.csv"))
temp1_scaff <- fread(here("Reports", "output_BM-1_lmscaff.csv"))
temp2 <- fread(here("Reports", "output_BM-2_full.csv"))

temp1$StudyFraction <- "BM-1 Floating"
temp1_scaff$StudyFraction <- "BM-1 Scaffold"
temp2$StudyFraction <- "BM-2 Floating"

temp2 <- temp2[effect == "fixed", ]
temp2$df <- NULL

signif_p7 <- rbind(
  temp1,
  temp1_scaff,
  temp2
)

## LAZY FIXME
signif_p7$term <- gsub("rd7", "d21", signif_p7$term)
signif_p7$term <- gsub("rd14", "d28", signif_p7$term)

## annoying fiddle to correct issue - dose and day swapped
## (this fix works unless there are anagrams present)
## sort letters in each word to define equivalence classes
## then assign each equivalence class the first name in that class
sort_Word <- Vectorize(function(x) {
  do.call(paste0, as.list(naturalsort(strsplit(x, "")[[1]])))
})
signif_p7$eq_class <- sort_Word(signif_p7$term)
temp <- rev(sort_Word(signif_p7$term)) ## rev ensures "last" element is taken
temp <- temp[!duplicated(temp)]
dict <- names(temp)
names(dict) <- temp
signif_p7$term <- dict[signif_p7$eq_class]
signif_p7$eq_class <- NULL

## subset to doses of interest
target_effects <- c(
  "factor(Dose)1:Dayd14", "factor(Dose)10:Dayd14",
  "factor(Dose)1:Dayd28", "factor(Dose)10:Dayd28"
)
signif_p7 <- signif_p7[signif_p7$term %in% target_effects, ]

## match plot
signif_p7$DoseName <- factor(signif_p7$term)
levels(signif_p7$DoseName) <- c("AZ1 Low", "AZ1 Low", "AZ1 High", "AZ1 High")
signif_p7$Day <- ifelse(
  substr(
    signif_p7$term,
    nchar(signif_p7$term) - 3, nchar(signif_p7$term)
  ) == "yd14",
  "d14",
  "d28"
)
signif_p7$Colour <- "Black"
colnames(signif_p7)[colnames(signif_p7) == "param"] <- "Parameter"
signif_p7 <- merge(signif_p7, toPlot,
  by = c("Day", "DoseName", "Parameter", "StudyFraction")
)
signif_p7$x <- ifelse(signif_p7$Day == "d14", 3, 5.5)
signif_p7 <- signif_p7[StudyFraction != "BM-2 Scaffold", ]
signif_p7$fit <- ifelse(signif_p7$Day == "d14",
  signif_p7$fit - 0.8,
  signif_p7$fit
) - 0.1
signif_p7$Parameter <- factor(signif_p7$Parameter,
  levels = levels(toPlot$Parameter)
)

p7 <- ggplot(
  toPlot[toPlot$Study != "BM-3" & toPlot$StudyFraction != "BM-2 Scaffold", ],
  aes_string(
    x = "Day",
    y = "fit",
    colour = "DoseName",
    fill = "DoseName",
    group = "DoseName",
    ymax = "upperSE",
    ymin = "lowerSE"
  )
) +
  geom_rect(aes(xmin = "d0", xmax = "d14", ymin = -Inf, ymax = Inf),
            fill = "grey92", colour = NA) +
  geom_point() +
  geom_line() +
  geom_ribbon(alpha = 0.3) +
  scale_colour_manual(values = cbbPalette[-1]) +
  scale_fill_manual(values = cbbPalette[-1]) +
  coord_cartesian(ylim = c(-1, 12.5)) +
  ylab("log10(Number of cells with marker)") +
  facet_grid(StudyFraction ~ Parameter) +
  theme_bw()
print(p7)
```

```
p7_final <- p7 +
  geom_text(aes(x = x, y = fit, label = stars),
            data = signif_p7, hjust = 0.5, vjust = 0.5, colour = "Black") +
  expand_limits(x = 6.5) +
  scale_colour_manual(values = c(cbbPalette[2:6], "Black"))
```

```
## Scale for colour is already present.
## Adding another scale for colour, which will replace the existing scale.
```

```
p7_final
```

```
signif_p8 <- fread(here("Reports", "output_BM-3_full.csv"))

## subset to doses of interest
signif_p8 <- signif_p8[
  signif_p8$term %in% c("factor(Dose)1:Dayd14", "factor(Dose)10:Dayd14",
                        "factor(Dose)20:Dayd14", "factor(Dose)50:Dayd14"), ]

## match plot
signif_p8$DoseName <- factor(signif_p8$term)
levels(signif_p8$DoseName) <- c("Carboplatin 1", "Carboplatin 10",
                                "Carboplatin 20", "Carboplatin 50")
signif_p8$Day <- "d14"
signif_p8$Colour <- "Black"
colnames(signif_p8)[colnames(signif_p8) == "param"] <- "Parameter"
signif_p8 <- merge(signif_p8,
                   toPlot_p8,
                   by = c("Day", "DoseName", "Parameter"))
signif_p8$x <- 7.75
signif_p8$Parameter <- factor(signif_p8$Parameter,
                              levels = levels(toPlot$Parameter))

p8_final <- p8 +
  geom_text(aes(x = x, y = fit, label = stars),
            data = signif_p8, hjust = 0.5, colour = "Black") +
  expand_limits(x = 8.5) +
  scale_colour_manual(values = c(cbbPalette[2:6], "Black"))
```

```
## Scale for colour is already present.
## Adding another scale for colour, which will replace the existing scale.
```

```
p8_final
```

```
p7_final / p8_final + plot_layout(heights = c(8, 3))
```

# Power analyses

```
## a useful function
powerCurve <- function(effects, sd, n) {
  data.frame(
    power = sapply(
      effects,
      function(effect) {
        power.t.test(
          n = n,
          delta = effect,
          sd = sd,
          type = "two.sample",
          alternative = "two.sided"
        )$power
      }
    ),
    effects = effects,
    sd = sd,
    n = n
  )
}


for (j in c("BM-1 Floating", "BM-1 Scaffold",
            "BM-2 Floating", "BM-3 Floating")) {
  ## transform
  testSetA <- full_gathered %>%
    filter(Parameter == "LateErythroid") %>%
    filter(StudyFraction == j)
  testSetA$logVal <- log(testSetA$value + 1)

  if (j == "BM-2 Floating") {
    ## MIXED effects model - Olap
    out.mixed <- lmer(logVal ~ Day + Day:DoseName + (1 | TubeNo),
                      data = testSetA)
    summary(out.mixed, ddf = "Kenward-Roger")

    ## Fetch some effect sizes of interest:
    out_Data <- coef(summary(out.mixed))

    selCoefs <- grepl("d21", row.names(out_Data)) |
      grepl("Dayd14", row.names(out_Data))
    selCoefs <- selCoefs & grepl("DoseName", row.names(out_Data))
    sds <- attr(VarCorr(out.mixed), "sc")
  } else {
    if (j == "BM-3 Floating") {
      ## MIXED effects model - carboplatin
      out.mixed <- lmer(logVal ~ Day + Day:DoseName + (1 | TubeNo),
                        data = testSetA)
      summary(out.mixed, ddf = "Kenward-Roger")

      ## Fetch some effect sizes of interest:
      out_Data <- coef(summary(out.mixed))

      selCoefs <- grepl("d21", row.names(out_Data)) |
        grepl("Dayd14", row.names(out_Data)) |
        grepl("Dayd7", row.names(out_Data))
      selCoefs <- selCoefs & grepl("DoseName", row.names(out_Data))

      sds <- attr(VarCorr(out.mixed), "sc")
    } else {
      ## FIXED effects model
      out.fix <- lm(logVal ~ Day + Day:DoseName, data = testSetA)
      summary(out.fix)

      ## Fetch some effect sizes of interest:
      out_Data <- coef(summary(out.fix))

      selCoefs <- grepl("d21", row.names(out_Data)) |
        grepl("Dayd14", row.names(out_Data))

      selCoefs <- selCoefs & grepl("DoseName", row.names(out_Data))

      sds <- summary(out.fix)$sigma
    }
  }

  out_Data <- as.data.frame(out_Data)[selCoefs, ]
  out_Data$my_Label <- rownames(out_Data)
  out_Data$absEstimate <- abs(out_Data$Estimate)

  ## Apply to power analysis:


  effects <- seq(from = 0.0, to = 10, by = 0.01)

  myData <- rbind(
    powerCurve(effects = effects, sd = sds, n = 7),
    powerCurve(effects = effects, sd = sds, n = 6),
    powerCurve(effects = effects, sd = sds, n = 5),
    powerCurve(effects = effects, sd = sds, n = 4),
    powerCurve(effects = effects, sd = sds, n = 3),
    powerCurve(effects = effects, sd = sds, n = 2)
  )

  myData$n <- factor(myData$n)

  out_Data$my_Y <- 0.5
  p <- ggplot(
    myData,
    aes_string(x = "effects", y = "power", colour = "n", group = "n")
  ) +
    geom_line() +
    geom_hline(yintercept = 0.8) +
    geom_hline(yintercept = 0.9) +
    ggtitle(
      paste0("Power calculation: Effect on Late Erythroid lineage (", j, ")")
    ) +
    scale_color_manual(values = cbbPalette)

  for (i in seq_len(nrow(out_Data))) {
    p <- p +
      geom_vline(xintercept = abs(out_Data[i, 1]), col = "blue")
  }

  p <- p + geom_text(
    aes(x = absEstimate, label = my_Label, y = my_Y, group = my_Label),
    colour = "blue", angle = 90, vjust = 1.2, text = element_text(size = 11),
    data = out_Data
  )

  print(p)
}
```

```
## Warning in geom_text(aes(x = absEstimate, label = my_Label, y = my_Y, group = my_Label), : Ignoring unknown parameters: `text`
## Ignoring unknown parameters: `text`
```

```
## Warning in geom_text(aes(x = absEstimate, label = my_Label, y = my_Y, group =
## my_Label), : Ignoring unknown parameters: `text`
```

```
## Warning in geom_text(aes(x = absEstimate, label = my_Label, y = my_Y, group =
## my_Label), : Ignoring unknown parameters: `text`
```

```
pander::pander(sessionInfo())
```

**R version 4.2.1 (2022-06-23 ucrt)**

**Platform:** x86\_64-w64-mingw32/x64 (64-bit)

**locale:** *LC\_COLLATE=English\_United
Kingdom.utf8*, *LC\_CTYPE=English\_United Kingdom.utf8*,
*LC\_MONETARY=English\_United Kingdom.utf8*, *LC\_NUMERIC=C*
and *LC\_TIME=English\_United Kingdom.utf8*

**attached base packages:** *grid*,
*stats*, *graphics*, *grDevices*, *utils*,
*datasets*, *methods* and *base*

**other attached packages:** *here(v.1.0.1)*,
*naturalsort(v.0.1.3)*, *patchwork(v.1.1.2)*,
*MASS(v.7.3-57)*, *lmerTest(v.3.1-3)*,
*lme4(v.1.1-30)*, *Matrix(v.1.5-1)*,
*tidyr(v.1.2.1)*, *dplyr(v.1.0.10)*,
*data.table(v.1.14.2)*, *magrittr(v.2.0.3)*,
*ggsignif(v.0.6.4)*, *ggbiplot(v.0.55)*,
*scales(v.1.2.1)*, *plyr(v.1.8.7)* and
*ggplot2(v.3.4.0)*

**loaded via a namespace (and not attached):**
*Rcpp(v.1.0.9)*, *lattice(v.0.20-45)*,
*assertthat(v.0.2.1)*, *rprojroot(v.2.0.3)*,
*digest(v.0.6.29)*, *utf8(v.1.2.2)*, *R6(v.2.5.1)*,
*backports(v.1.4.1)*, *evaluate(v.0.17)*,
*highr(v.0.9)*, *pillar(v.1.8.1)*,
*rlang(v.1.0.6)*, *rstudioapi(v.0.14)*,
*minqa(v.1.2.4)*, *jquerylib(v.0.1.4)*,
*nloptr(v.2.0.3)*, *rmarkdown(v.2.17)*,
*labeling(v.0.4.2)*, *splines(v.4.2.1)*,
*pander(v.0.6.5)*, *stringr(v.1.4.1)*,
*munsell(v.0.5.0)*, *broom(v.1.0.1)*,
*compiler(v.4.2.1)*, *numDeriv(v.2016.8-1.1)*,
*xfun(v.0.31)*, *pkgconfig(v.2.0.3)*,
*htmltools(v.0.5.3)*, *tidyselect(v.1.2.0)*,
*tibble(v.3.1.8)*, *fansi(v.1.0.3)*,
*withr(v.2.5.0)*, *nlme(v.3.1-157)*,
*jsonlite(v.1.8.2)*, *gtable(v.0.3.1)*,
*lifecycle(v.1.0.3)*, *DBI(v.1.1.3)*,
*cli(v.3.4.1)*, *stringi(v.1.7.8)*,
*cachem(v.1.0.6)*, *farver(v.2.1.1)*,
*bslib(v.0.4.0)*, *ellipsis(v.0.3.2)*,
*generics(v.0.1.3)*, *vctrs(v.0.5.1)*,
*boot(v.1.3-28)*, *tools(v.4.2.1)*,
*glue(v.1.6.2)*, *purrr(v.0.3.5)*,
*parallel(v.4.2.1)*, *pbkrtest(v.0.5.1)*,
*fastmap(v.1.1.0)*, *yaml(v.2.3.5)*,
*colorspace(v.2.0-3)*, *knitr(v.1.40)* and
*sass(v.0.4.2)*
