# Supplementary material for: Optimal experimental design for efficient toxicity testing in microphysiological systems: A bone marrow application
Source: Front Pharmacol. 2023 Mar 31;14:1142581. doi: 10.3389/fphar.2023.1142581 (PMC10103791; doi:10.3389/fphar.2023.1142581)
Supplement: Supplementary file 2 [file DataSheet1.zip › Data package/Reports/compare-PAPER-fig3_counts.html]

Bone Marrow MPS - compare 2018-04/05


Code 

- Show All Code
- Hide All Code

# Bone Marrow MPS - compare 2018-04/05

#### Statistician: Jonathan Cairns

#### 5 April 2019

Read in data:

```
source("createFull.R")
```

```
## 
## Attaching package: 'MASS'
```

```
## The following object is masked from 'package:dplyr':
## 
##     select
```

```
## Warning: package 'naturalsort' was built under R version 4.2.2
```

```
## create full_gathered
interesting_cols <- c("EarlyErythroid", "LateErythroid", "EarlyMyeloid", "ltHSC", "Platelets", "LineageDiffd", "LateGranulocytes")
metadata_cols <- c(colnames(full)[1:10], "Study", "Group", "Day", "StudyFraction")

## specify the columns of interest
my_sel <- c(
  colnames(full) %>% grep(pattern = "CD"),
  colnames(full) %>% grep(pattern = "DeadCells_pct"),
  which(colnames(full) %in% c(interesting_cols, paste0(interesting_cols, "_pct")))
)

full_gathered <- full %>% gather_(
  key_col      = "Parameter",
  value_col    = "value",
  gather_cols  = colnames(full)[my_sel]
)
```

```
## Warning: `gather_()` was deprecated in tidyr 1.2.0.
## ℹ Please use `gather()` instead.
```

## Heatmap of residuals

###Dead cells

```
full.toModel <- full_gathered[grepl(pattern = "_pct", full_gathered$Parameter), ]

for (j in c("BM-1 Floating", "BM-1 Scaffold",
            "BM-2 Floating", "BM-3 Floating")) {
  testSet <- full.toModel[full.toModel$StudyFraction == j, ] ## grab data
  testSet$value <- testSet$value / 100.0 ## reduce to proportion
  testSet$Condition <- with(testSet, paste0(DoseName, "-", Day))
  ## replace zeroes with minimal alternative value
  testSet.noZeroes <- testSet
  sel <- testSet.noZeroes$value == 0
  testSet.noZeroes[sel, "value"] <- min(testSet.noZeroes[!sel, "value"])

  model3 <- lm(logit(value) ~ Condition * Parameter, data = testSet.noZeroes)

  temp <- testSet.noZeroes
  temp$resid <- resid(model3)
  temp <- temp[temp$Parameter == "DeadCells_pct", ]

  ## deduce location in incubator
  temp$col <- ((as.integer(temp$TubeNo) - 1) %% 8) + 1
  temp$row <- 1 + (as.integer(temp$TubeNo) - temp$col) / 8
  temp$row <- 7 - temp$row ## invert

  p <- ggplot(temp, aes(col, row)) +
    geom_tile(aes(fill = resid),
      colour = "white"
    ) +
    scale_fill_gradient2(
      low = "blue",
      high = "yellow"
    )
  print(p)
}
```

###Late Erythroid

```
for (j in c("BM-1 Floating", "BM-1 Scaffold",
            "BM-2 Floating", "BM-3 Floating")) {
  testSet <- full.toModel[full.toModel$StudyFraction == j, ] ## grab data
  testSet$value <- testSet$value / 100.0 ## reduce to proportion
  testSet$Condition <- with(testSet, paste0(DoseName, "-", Day))
  ## replace zeroes with minimal alternative value
  testSet.noZeroes <- testSet
  sel <- testSet.noZeroes$value == 0
  testSet.noZeroes[sel, "value"] <- min(testSet.noZeroes[!sel, "value"])

  model3 <- lm(logit(value) ~ Condition * Parameter, data = testSet.noZeroes)

  temp <- testSet.noZeroes
  temp$resid <- resid(model3)
  temp <- temp[temp$Parameter == "LateErythroid_pct", ]

  ## deduce location in incubator
  temp$col <- ((as.integer(temp$TubeNo) - 1) %% 8) + 1
  temp$row <- 1 + (as.integer(temp$TubeNo) - temp$col) / 8
  temp$row <- 7 - temp$row ## invert

  p <- ggplot(temp, aes(col, row)) +
    geom_tile(aes(fill = resid),
      colour = "white"
    ) +
    scale_fill_gradient2(
      low = "blue",
      high = "yellow"
    )
  print(p)
}
```

Fig 6 panels B-E:

```
for (j in c("BM-1 Floating", "BM-1 Scaffold",
            "BM-2 Floating", "BM-3 Floating")) {
  testSet <- full.toModel[full.toModel$StudyFraction == j, ] ## grab data
  testSet$value <- testSet$value / 100.0 ## reduce to proportion
  testSet$Condition <- with(testSet, paste0(DoseName, "-", Day))
  ## replace zeroes with minimal alternative value
  testSet.noZeroes <- testSet
  sel <- testSet.noZeroes$value == 0
  testSet.noZeroes[sel, "value"] <- min(testSet.noZeroes[!sel, "value"])

  model3 <- lm(logit(value) ~ Condition * Parameter, data = testSet.noZeroes)

  temp <- testSet.noZeroes
  temp$resid <- resid(model3)
  temp <- temp[temp$Parameter == "LateErythroid_pct", ]

  ## deduce location in incubator
  temp$col <- ((as.integer(temp$TubeNo) - 1) %% 8) + 1
  temp$row <- 1 + (as.integer(temp$TubeNo) - temp$col) / 8
  temp$row <- 7 - temp$row ## invert

  p <- ggplot(temp, aes(col, row)) +
    geom_tile(aes(fill = resid),
      colour = "white"
    ) +
    scale_fill_gradient2(
      low = "blue",
      high = "yellow"
    ) +
    theme(
      plot.title = element_text(size=20),
      legend.title = element_text(size=20),
      legend.text = element_text(size=20),
      legend.key.width = unit(1.5,"cm"),
      legend.key.height = unit(1.5,"cm"),
      axis.text.x=element_blank(),
      axis.ticks.x=element_blank(),
      axis.title.x=element_blank(),
      axis.text.y=element_blank(),
      axis.ticks.y=element_blank(),
      axis.title.y=element_blank(),
      panel.grid.major = element_blank(),
      panel.grid.minor = element_blank()
    )
  print(p)
}
```

## flusher, incubator

```
for (j in c("BM-1 Floating", "BM-1 Scaffold",
            "BM-2 Floating", "BM-3 Floating")) {
  testSet <- full.toModel[full.toModel$StudyFraction == j, ] ## grab data
  testSet$value <- testSet$value / 100.0 ## reduce to proportion
  testSet$Condition <- with(testSet, paste0(DoseName, "-", Day))
  ## replace zeroes with minimal alternative value
  testSet.noZeroes <- testSet
  sel <- testSet.noZeroes$value == 0
  testSet.noZeroes[sel, "value"] <- min(testSet.noZeroes[!sel, "value"])

  ## regress out the biology
  model3 <- lm(logit(value) ~ Condition * Parameter, data = testSet.noZeroes)

  temp <- testSet.noZeroes
  temp$resid <- resid(model3)
  temp <- temp[temp$Parameter == "DeadCells_pct", ]

  ## regress technical effects on
  model4 <- lm(resid ~ Flusher * Incubator, data = temp)
  print(summary(model4))
  print(anova(model4))
}
```

```
## 
## Call:
## lm(formula = resid ~ Flusher * Incubator, data = temp)
## 
## Residuals:
##      Min       1Q   Median       3Q      Max 
## -1.21712 -0.30146  0.00343  0.23664  0.82774 
## 
## Coefficients:
##                       Estimate Std. Error t value Pr(>|t|)  
## (Intercept)            -0.3131     0.1331  -2.353   0.0239 *
## FlusherH                0.3357     0.1929   1.740   0.0899 .
## IncubatorTop            0.3680     0.1984   1.855   0.0714 .
## FlusherH:IncubatorTop  -0.1635     0.2740  -0.597   0.5542  
## ---
## Signif. codes:  0 '***' 0.001 '**' 0.01 '*' 0.05 '.' 0.1 ' ' 1
## 
## Residual standard error: 0.4414 on 38 degrees of freedom
## Multiple R-squared:  0.1893, Adjusted R-squared:  0.1253 
## F-statistic: 2.958 on 3 and 38 DF,  p-value: 0.04444
## 
## Analysis of Variance Table
## 
## Response: resid
##                   Df Sum Sq Mean Sq F value  Pr(>F)  
## Flusher            1 0.8308 0.83082  4.2636 0.04581 *
## Incubator          1 0.8290 0.82904  4.2545 0.04603 *
## Flusher:Incubator  1 0.0694 0.06940  0.3561 0.55420  
## Residuals         38 7.4048 0.19486                  
## ---
## Signif. codes:  0 '***' 0.001 '**' 0.01 '*' 0.05 '.' 0.1 ' ' 1
## 
## Call:
## lm(formula = resid ~ Flusher * Incubator, data = temp)
## 
## Residuals:
##      Min       1Q   Median       3Q      Max 
## -0.50441 -0.19709 -0.04486  0.12414  0.87551 
## 
## Coefficients:
##                       Estimate Std. Error t value Pr(>|t|)
## (Intercept)           -0.04952    0.09366  -0.529    0.600
## FlusherH              -0.09413    0.13573  -0.693    0.492
## IncubatorTop           0.06527    0.13963   0.467    0.643
## FlusherH:IncubatorTop  0.23168    0.19284   1.201    0.237
## 
## Residual standard error: 0.3106 on 38 degrees of freedom
## Multiple R-squared:  0.1237, Adjusted R-squared:  0.0545 
## F-statistic: 1.788 on 3 and 38 DF,  p-value: 0.1659
## 
## Analysis of Variance Table
## 
## Response: resid
##                   Df Sum Sq Mean Sq F value  Pr(>F)  
## Flusher            1 0.0155 0.01551  0.1607 0.69076  
## Incubator          1 0.3628 0.36277  3.7592 0.05997 .
## Flusher:Incubator  1 0.1393 0.13929  1.4434 0.23702  
## Residuals         38 3.6671 0.09650                  
## ---
## Signif. codes:  0 '***' 0.001 '**' 0.01 '*' 0.05 '.' 0.1 ' ' 1
## 
## Call:
## lm(formula = resid ~ Flusher * Incubator, data = temp)
## 
## Residuals:
##      Min       1Q   Median       3Q      Max 
## -0.86859 -0.17147  0.00051  0.19215  0.64163 
## 
## Coefficients: (1 not defined because of singularities)
##                       Estimate Std. Error t value Pr(>|t|)
## (Intercept)           -0.12546    0.09940  -1.262    0.210
## FlusherC              -0.02524    0.12174  -0.207    0.836
## FlusherH               0.15290    0.12174   1.256    0.213
## IncubatorTop           0.12326    0.09940   1.240    0.218
## FlusherC:IncubatorTop  0.05097    0.13459   0.379    0.706
## FlusherH:IncubatorTop       NA         NA      NA       NA
## 
## Residual standard error: 0.2982 on 85 degrees of freedom
## Multiple R-squared:  0.1156, Adjusted R-squared:  0.07395 
## F-statistic: 2.777 on 4 and 85 DF,  p-value: 0.03199
## 
## Analysis of Variance Table
## 
## Response: resid
##                   Df Sum Sq Mean Sq F value  Pr(>F)  
## Flusher            2 0.5231 0.26157  2.9414 0.05819 .
## Incubator          1 0.4518 0.45179  5.0805 0.02677 *
## Flusher:Incubator  1 0.0128 0.01275  0.1434 0.70588  
## Residuals         85 7.5588 0.08893                  
## ---
## Signif. codes:  0 '***' 0.001 '**' 0.01 '*' 0.05 '.' 0.1 ' ' 1
## 
## Call:
## lm(formula = resid ~ Flusher * Incubator, data = temp)
## 
## Residuals:
##      Min       1Q   Median       3Q      Max 
## -1.04887 -0.21723 -0.01386  0.24427  0.95792 
## 
## Coefficients:
##                        Estimate Std. Error t value Pr(>|t|)    
## (Intercept)            0.093627   0.074114   1.263 0.207958    
## FlusherC               0.111887   0.104813   1.067 0.287036    
## FlusherH              -0.006034   0.092906  -0.065 0.948282    
## FlusherI              -0.297901   0.148227  -2.010 0.045800 *  
## IncubatorTop          -0.365457   0.104813  -3.487 0.000601 ***
## FlusherC:IncubatorTop  0.113816   0.148227   0.768 0.443483    
## FlusherH:IncubatorTop  0.236159   0.128708   1.835 0.068014 .  
## FlusherI:IncubatorTop  0.652115   0.209625   3.111 0.002138 ** 
## ---
## Signif. codes:  0 '***' 0.001 '**' 0.01 '*' 0.05 '.' 0.1 ' ' 1
## 
## Residual standard error: 0.3631 on 200 degrees of freedom
## Multiple R-squared:  0.1275, Adjusted R-squared:  0.09696 
## F-statistic: 4.175 on 7 and 200 DF,  p-value: 0.0002583
## 
## Analysis of Variance Table
## 
## Response: resid
##                    Df  Sum Sq Mean Sq F value    Pr(>F)    
## Flusher             3  0.7666 0.25552  1.9383 0.1246331    
## Incubator           1  1.6801 1.68012 12.7448 0.0004471 ***
## Flusher:Incubator   3  1.4061 0.46871  3.5554 0.0153314 *  
## Residuals         200 26.3656 0.13183                      
## ---
## Signif. codes:  0 '***' 0.001 '**' 0.01 '*' 0.05 '.' 0.1 ' ' 1
```

```
for (j in c("BM-1 Floating", "BM-1 Scaffold",
            "BM-2 Floating", "BM-3 Floating")) {
  testSet <- full.toModel[full.toModel$StudyFraction == j, ] ## grab data
  testSet$value <- testSet$value / 100.0 ## reduce to proportion
  testSet$Condition <- with(testSet, paste0(DoseName, "-", Day))
  ## replace zeroes with minimal alternative value
  testSet.noZeroes <- testSet
  sel <- testSet.noZeroes$value == 0
  testSet.noZeroes[sel, "value"] <- min(testSet.noZeroes[!sel, "value"])

  ## regress out the biology
  model3 <- lm(logit(value) ~ Condition * Parameter, data = testSet.noZeroes)

  temp <- testSet.noZeroes
  temp$resid <- resid(model3)
  temp <- temp[temp$Parameter == "DeadCells_pct", ]

  ## regress technical effects on
  model4 <- lm(resid ~ ControlUnit + Side, data = temp)
  print(summary(model4))
  print(anova(model4))
}
```

```
## 
## Call:
## lm(formula = resid ~ ControlUnit + Side, data = temp)
## 
## Residuals:
##      Min       1Q   Median       3Q      Max 
## -0.91591 -0.29015 -0.00041  0.27571  0.94774 
## 
## Coefficients:
##               Estimate Std. Error t value Pr(>|t|)  
## (Intercept)     0.2872     0.1762   1.631   0.1120  
## ControlUnit24  -0.5336     0.2449  -2.179   0.0361 *
## ControlUnit25  -0.2083     0.2278  -0.914   0.3668  
## ControlUnit26  -0.5937     0.2278  -2.606   0.0134 *
## ControlUnit27  -0.4643     0.2449  -1.896   0.0662 .
## ControlUnit28  -0.5678     0.2359  -2.407   0.0215 *
## SideR           0.2054     0.1362   1.509   0.1404  
## ---
## Signif. codes:  0 '***' 0.001 '**' 0.01 '*' 0.05 '.' 0.1 ' ' 1
## 
## Residual standard error: 0.4398 on 35 degrees of freedom
## Multiple R-squared:  0.259,  Adjusted R-squared:  0.1319 
## F-statistic: 2.039 on 6 and 35 DF,  p-value: 0.08642
## 
## Analysis of Variance Table
## 
## Response: resid
##             Df Sum Sq Mean Sq F value Pr(>F)
## ControlUnit  5 1.9253 0.38507  1.9912 0.1042
## Side         1 0.4401 0.44014  2.2760 0.1404
## Residuals   35 6.7686 0.19339               
## 
## Call:
## lm(formula = resid ~ ControlUnit + Side, data = temp)
## 
## Residuals:
##     Min      1Q  Median      3Q     Max 
## -0.4097 -0.2138 -0.0406  0.1529  0.8875 
## 
## Coefficients:
##               Estimate Std. Error t value Pr(>|t|)  
## (Intercept)    0.10427    0.12675   0.823   0.4163  
## ControlUnit24  0.09102    0.17618   0.517   0.6087  
## ControlUnit25 -0.11497    0.16391  -0.701   0.4877  
## ControlUnit26 -0.16577    0.16391  -1.011   0.3188  
## ControlUnit27 -0.34154    0.17618  -1.939   0.0607 .
## ControlUnit28 -0.13963    0.16971  -0.823   0.4162  
## SideR          0.01652    0.09798   0.169   0.8671  
## ---
## Signif. codes:  0 '***' 0.001 '**' 0.01 '*' 0.05 '.' 0.1 ' ' 1
## 
## Residual standard error: 0.3164 on 35 degrees of freedom
## Multiple R-squared:  0.1626, Adjusted R-squared:  0.01902 
## F-statistic: 1.132 on 6 and 35 DF,  p-value: 0.3642
## 
## Analysis of Variance Table
## 
## Response: resid
##             Df Sum Sq  Mean Sq F value Pr(>F)
## ControlUnit  5 0.6775 0.135494  1.3533 0.2655
## Side         1 0.0028 0.002847  0.0284 0.8671
## Residuals   35 3.5043 0.100123               
## 
## Call:
## lm(formula = resid ~ ControlUnit + Side, data = temp)
## 
## Residuals:
##      Min       1Q   Median       3Q      Max 
## -0.89759 -0.15002 -0.01127  0.16461  0.71299 
## 
## Coefficients:
##               Estimate Std. Error t value Pr(>|t|)   
## (Intercept)    0.17517    0.09929   1.764  0.08135 . 
## ControlUnit24 -0.16886    0.11518  -1.466  0.14642   
## ControlUnit25 -0.09736    0.12188  -0.799  0.42665   
## ControlUnit26 -0.06156    0.13300  -0.463  0.64469   
## ControlUnit27 -0.29687    0.11146  -2.663  0.00929 **
## ControlUnit28 -0.34958    0.13300  -2.628  0.01022 * 
## SideR          0.01512    0.06365   0.238  0.81275   
## ---
## Signif. codes:  0 '***' 0.001 '**' 0.01 '*' 0.05 '.' 0.1 ' ' 1
## 
## Residual standard error: 0.2974 on 83 degrees of freedom
## Multiple R-squared:  0.141,  Adjusted R-squared:  0.07893 
## F-statistic: 2.271 on 6 and 83 DF,  p-value: 0.0444
## 
## Analysis of Variance Table
## 
## Response: resid
##             Df Sum Sq  Mean Sq F value  Pr(>F)  
## ControlUnit  5 1.2003 0.240058  2.7141 0.02541 *
## Side         1 0.0050 0.004995  0.0565 0.81275  
## Residuals   83 7.3412 0.088448                  
## ---
## Signif. codes:  0 '***' 0.001 '**' 0.01 '*' 0.05 '.' 0.1 ' ' 1
## 
## Call:
## lm(formula = resid ~ ControlUnit + Side, data = temp)
## 
## Residuals:
##     Min      1Q  Median      3Q     Max 
## -1.1662 -0.2184  0.0024  0.2430  1.0063 
## 
## Coefficients:
##               Estimate Std. Error t value Pr(>|t|)  
## (Intercept)   -0.01645    0.06441  -0.255   0.7987  
## ControlUnit24 -0.02846    0.08242  -0.345   0.7302  
## ControlUnit25 -0.05097    0.09193  -0.554   0.5799  
## ControlUnit26  0.10060    0.09193   1.094   0.2751  
## ControlUnit27  0.19737    0.08242   2.395   0.0176 *
## ControlUnit28  0.14283    0.09193   1.554   0.1218  
## SideR         -0.08713    0.05174  -1.684   0.0937 .
## ---
## Signif. codes:  0 '***' 0.001 '**' 0.01 '*' 0.05 '.' 0.1 ' ' 1
## 
## Residual standard error: 0.3731 on 201 degrees of freedom
## Multiple R-squared:  0.07423,    Adjusted R-squared:  0.04659 
## F-statistic: 2.686 on 6 and 201 DF,  p-value: 0.01573
## 
## Analysis of Variance Table
## 
## Response: resid
##              Df  Sum Sq Mean Sq F value  Pr(>F)  
## ControlUnit   5  1.8483 0.36965  2.6559 0.02381 *
## Side          1  0.3948 0.39479  2.8365 0.09369 .
## Residuals   201 27.9754 0.13918                  
## ---
## Signif. codes:  0 '***' 0.001 '**' 0.01 '*' 0.05 '.' 0.1 ' ' 1
```

Test for effect of flusher, conditional on control unit.

```
for (j in c("BM-1 Floating", "BM-1 Scaffold",
            "BM-2 Floating", "BM-3 Floating")) {
  print("")
  print("")
  print(j)
  testSet <- full.toModel[full.toModel$StudyFraction == j, ] ## grab data
  testSet$value <- testSet$value / 100.0 ## reduce to proportion
  testSet$Condition <- with(testSet, paste0(DoseName, "-", Day))
  ## replace zeroes with minimal alternative value
  testSet.noZeroes <- testSet
  sel <- testSet.noZeroes$value == 0
  testSet.noZeroes[sel, "value"] <- min(testSet.noZeroes[!sel, "value"])

  ## regress out the biology
  model3 <- lm(logit(value) ~ Condition * Parameter, data = testSet.noZeroes)

  temp <- testSet.noZeroes
  temp$resid <- resid(model3)
  temp <- temp[temp$Parameter == "DeadCells_pct", ]

  p <- ggplot(temp, aes(x = ControlUnit, y = resid, col = Flusher)) +
    # facet_grid(. ~ Incubator) +
    geom_point() +
    scale_color_manual(values = cbbPalette) +
    ylim(-1.5, 1.5)

  print(p)

  p <- ggplot(temp, aes(x = ControlUnit, y = resid, fill = Flusher)) +
    # facet_grid(. ~ Incubator) +
    geom_boxplot() +
    scale_color_manual(values = cbbPalette) +
    ylim(-1.5, 1.5)
  print(p)

  model_incubator <- lm(resid ~ Incubator + Flusher + Side, data = temp,
                        contrasts = list(Incubator = contr.sum, Flusher = contr.sum))
  model_cu <- lm(resid ~ ControlUnit + Flusher + Side, data = temp,
                 contrasts = list(ControlUnit = contr.sum, Flusher = contr.sum))

  print(anova(model_incubator, model_cu))
  print(anova(model_incubator))
  print(summary(model_incubator))
  print(anova(model_cu))
  print(summary(model_cu))
}
```

```
## [1] ""
## [1] ""
## [1] "BM-1 Floating"
```

```
## Analysis of Variance Table
## 
## Model 1: resid ~ Incubator + Flusher + Side
## Model 2: resid ~ ControlUnit + Flusher + Side
##   Res.Df    RSS Df Sum of Sq      F Pr(>F)
## 1     38 7.1756                           
## 2     34 5.8412  4    1.3344 1.9418 0.1259
## Analysis of Variance Table
## 
## Response: resid
##           Df Sum Sq Mean Sq F value  Pr(>F)  
## Incubator  1 0.9866 0.98659  5.2247 0.02793 *
## Flusher    1 0.6733 0.67326  3.5654 0.06665 .
## Side       1 0.2986 0.29858  1.5812 0.21626  
## Residuals 38 7.1756 0.18883                  
## ---
## Signif. codes:  0 '***' 0.001 '**' 0.01 '*' 0.05 '.' 0.1 ' ' 1
## 
## Call:
## lm(formula = resid ~ Incubator + Flusher + Side, data = temp, 
##     contrasts = list(Incubator = contr.sum, Flusher = contr.sum))
## 
## Residuals:
##      Min       1Q   Median       3Q      Max 
## -1.09896 -0.21547 -0.02587  0.20360  0.88842 
## 
## Coefficients:
##             Estimate Std. Error t value Pr(>|t|)  
## (Intercept) -0.09050    0.09495  -0.953   0.3465  
## Incubator1  -0.14599    0.06747  -2.164   0.0368 *
## Flusher1    -0.11877    0.06778  -1.752   0.0878 .
## SideR        0.16968    0.13494   1.257   0.2163  
## ---
## Signif. codes:  0 '***' 0.001 '**' 0.01 '*' 0.05 '.' 0.1 ' ' 1
## 
## Residual standard error: 0.4345 on 38 degrees of freedom
## Multiple R-squared:  0.2144, Adjusted R-squared:  0.1524 
## F-statistic: 3.457 on 3 and 38 DF,  p-value: 0.02571
## 
## Analysis of Variance Table
## 
## Response: resid
##             Df Sum Sq Mean Sq F value  Pr(>F)  
## ControlUnit  5 1.9253 0.38507  2.2414 0.07255 .
## Flusher      1 1.0494 1.04937  6.1081 0.01862 *
## Side         1 0.3182 0.31817  1.8520 0.18251  
## Residuals   34 5.8412 0.17180                  
## ---
## Signif. codes:  0 '***' 0.001 '**' 0.01 '*' 0.05 '.' 0.1 ' ' 1
## 
## Call:
## lm(formula = resid ~ ControlUnit + Flusher + Side, data = temp, 
##     contrasts = list(ControlUnit = contr.sum, Flusher = contr.sum))
## 
## Residuals:
##      Min       1Q   Median       3Q      Max 
## -0.70703 -0.31868 -0.01564  0.24314  0.87683 
## 
## Coefficients:
##              Estimate Std. Error t value Pr(>|t|)   
## (Intercept)  -0.10442    0.09093  -1.148  0.25879   
## ControlUnit1  0.47642    0.14776   3.224  0.00279 **
## ControlUnit2 -0.18290    0.15360  -1.191  0.24200   
## ControlUnit3  0.11436    0.13937   0.821  0.41759   
## ControlUnit4 -0.10319    0.14202  -0.727  0.47243   
## ControlUnit5 -0.16958    0.15839  -1.071  0.29185   
## Flusher1     -0.16787    0.07225  -2.323  0.02627 * 
## SideR         0.17554    0.12899   1.361  0.18251   
## ---
## Signif. codes:  0 '***' 0.001 '**' 0.01 '*' 0.05 '.' 0.1 ' ' 1
## 
## Residual standard error: 0.4145 on 34 degrees of freedom
## Multiple R-squared:  0.3605, Adjusted R-squared:  0.2288 
## F-statistic: 2.738 on 7 and 34 DF,  p-value: 0.0229
## 
## [1] ""
## [1] ""
## [1] "BM-1 Scaffold"
```

```
## Analysis of Variance Table
## 
## Model 1: resid ~ Incubator + Flusher + Side
## Model 2: resid ~ ControlUnit + Flusher + Side
##   Res.Df    RSS Df Sum of Sq      F Pr(>F)
## 1     38 3.8033                           
## 2     34 3.4457  4   0.35769 0.8824 0.4848
## Analysis of Variance Table
## 
## Response: resid
##           Df Sum Sq Mean Sq F value  Pr(>F)  
## Incubator  1 0.3739 0.37385  3.7352 0.06075 .
## Flusher    1 0.0044 0.00443  0.0442 0.83455  
## Side       1 0.0030 0.00300  0.0300 0.86347  
## Residuals 38 3.8033 0.10009                  
## ---
## Signif. codes:  0 '***' 0.001 '**' 0.01 '*' 0.05 '.' 0.1 ' ' 1
## 
## Call:
## lm(formula = resid ~ Incubator + Flusher + Side, data = temp, 
##     contrasts = list(Incubator = contr.sum, Flusher = contr.sum))
## 
## Residuals:
##      Min       1Q   Median       3Q      Max 
## -0.44548 -0.22204 -0.01546  0.10419  0.93826 
## 
## Coefficients:
##              Estimate Std. Error t value Pr(>|t|)  
## (Intercept) -0.008955   0.069125  -0.130   0.8976  
## Incubator1  -0.093850   0.049120  -1.911   0.0636 .
## Flusher1    -0.009467   0.049345  -0.192   0.8489  
## SideR        0.017008   0.098241   0.173   0.8635  
## ---
## Signif. codes:  0 '***' 0.001 '**' 0.01 '*' 0.05 '.' 0.1 ' ' 1
## 
## Residual standard error: 0.3164 on 38 degrees of freedom
## Multiple R-squared:  0.09111,    Adjusted R-squared:  0.01936 
## F-statistic:  1.27 on 3 and 38 DF,  p-value: 0.2985
## 
## Analysis of Variance Table
## 
## Response: resid
##             Df Sum Sq  Mean Sq F value Pr(>F)
## ControlUnit  5 0.6775 0.135494  1.3370 0.2724
## Flusher      1 0.0607 0.060667  0.5986 0.4444
## Side         1 0.0008 0.000837  0.0083 0.9281
## Residuals   34 3.4457 0.101343               
## 
## Call:
## lm(formula = resid ~ ControlUnit + Flusher + Side, data = temp, 
##     contrasts = list(ControlUnit = contr.sum, Flusher = contr.sum))
## 
## Residuals:
##      Min       1Q   Median       3Q      Max 
## -0.38505 -0.20098 -0.03347  0.15198  0.90484 
## 
## Coefficients:
##               Estimate Std. Error t value Pr(>|t|)  
## (Intercept)  -0.006798   0.069834  -0.097   0.9230  
## ControlUnit1  0.132387   0.113485   1.167   0.2515  
## ControlUnit2  0.191775   0.117975   1.626   0.1133  
## ControlUnit3 -0.021252   0.107039  -0.199   0.8438  
## ControlUnit4 -0.029827   0.109075  -0.273   0.7862  
## ControlUnit5 -0.254853   0.121647  -2.095   0.0437 *
## Flusher1     -0.042219   0.055495  -0.761   0.4520  
## SideR         0.009003   0.099073   0.091   0.9281  
## ---
## Signif. codes:  0 '***' 0.001 '**' 0.01 '*' 0.05 '.' 0.1 ' ' 1
## 
## Residual standard error: 0.3183 on 34 degrees of freedom
## Multiple R-squared:  0.1766, Adjusted R-squared:  0.007067 
## F-statistic: 1.042 on 7 and 34 DF,  p-value: 0.4212
## 
## [1] ""
## [1] ""
## [1] "BM-2 Floating"
```

```
## Analysis of Variance Table
## 
## Model 1: resid ~ Incubator + Flusher + Side
## Model 2: resid ~ ControlUnit + Flusher + Side
##   Res.Df    RSS Df Sum of Sq      F  Pr(>F)  
## 1     85 7.5639                              
## 2     81 6.8643  4   0.69954 2.0637 0.09319 .
## ---
## Signif. codes:  0 '***' 0.001 '**' 0.01 '*' 0.05 '.' 0.1 ' ' 1
## Analysis of Variance Table
## 
## Response: resid
##           Df Sum Sq Mean Sq F value  Pr(>F)  
## Incubator  1 0.4981 0.49810  5.5974 0.02026 *
## Flusher    2 0.4768 0.23842  2.6793 0.07441 .
## Side       1 0.0076 0.00765  0.0859 0.77014  
## Residuals 85 7.5639 0.08899                  
## ---
## Signif. codes:  0 '***' 0.001 '**' 0.01 '*' 0.05 '.' 0.1 ' ' 1
## 
## Call:
## lm(formula = resid ~ Incubator + Flusher + Side, data = temp, 
##     contrasts = list(Incubator = contr.sum, Flusher = contr.sum))
## 
## Residuals:
##      Min       1Q   Median       3Q      Max 
## -0.87424 -0.17821 -0.00794  0.18777  0.63597 
## 
## Coefficients:
##             Estimate Std. Error t value Pr(>|t|)  
## (Intercept) -0.01562    0.04964  -0.315   0.7538  
## Incubator1  -0.07553    0.03352  -2.253   0.0268 *
## Flusher1    -0.04255    0.07411  -0.574   0.5674  
## Flusher2    -0.05389    0.04997  -1.079   0.2838  
## SideR        0.01855    0.06328   0.293   0.7701  
## ---
## Signif. codes:  0 '***' 0.001 '**' 0.01 '*' 0.05 '.' 0.1 ' ' 1
## 
## Residual standard error: 0.2983 on 85 degrees of freedom
## Multiple R-squared:  0.115,  Adjusted R-squared:  0.07332 
## F-statistic:  2.76 on 4 and 85 DF,  p-value: 0.03278
## 
## Analysis of Variance Table
## 
## Response: resid
##             Df Sum Sq  Mean Sq F value  Pr(>F)  
## ControlUnit  5 1.2003 0.240058  2.8327 0.02081 *
## Flusher      2 0.4768 0.238420  2.8134 0.06588 .
## Side         1 0.0050 0.004995  0.0589 0.80880  
## Residuals   81 6.8643 0.084745                  
## ---
## Signif. codes:  0 '***' 0.001 '**' 0.01 '*' 0.05 '.' 0.1 ' ' 1
## 
## Call:
## lm(formula = resid ~ ControlUnit + Flusher + Side, data = temp, 
##     contrasts = list(ControlUnit = contr.sum, Flusher = contr.sum))
## 
## Residuals:
##      Min       1Q   Median       3Q      Max 
## -0.83519 -0.12884  0.01956  0.15312  0.62506 
## 
## Coefficients:
##               Estimate Std. Error t value Pr(>|t|)  
## (Intercept)   0.005426   0.050319   0.108   0.9144  
## ControlUnit1  0.163506   0.082829   1.974   0.0518 .
## ControlUnit2 -0.005354   0.063529  -0.084   0.9330  
## ControlUnit3  0.066143   0.070907   0.933   0.3537  
## ControlUnit4  0.099680   0.082829   1.203   0.2323  
## ControlUnit5 -0.135628   0.058951  -2.301   0.0240 *
## Flusher1     -0.042552   0.072327  -0.588   0.5579  
## Flusher2     -0.053892   0.048763  -1.105   0.2723  
## SideR         0.015125   0.062301   0.243   0.8088  
## ---
## Signif. codes:  0 '***' 0.001 '**' 0.01 '*' 0.05 '.' 0.1 ' ' 1
## 
## Residual standard error: 0.2911 on 81 degrees of freedom
## Multiple R-squared:  0.1968, Adjusted R-squared:  0.1175 
## F-statistic: 2.481 on 8 and 81 DF,  p-value: 0.01847
## 
## [1] ""
## [1] ""
## [1] "BM-3 Floating"
```

```
## Analysis of Variance Table
## 
## Model 1: resid ~ Incubator + Flusher + Side
## Model 2: resid ~ ControlUnit + Flusher + Side
##   Res.Df    RSS Df Sum of Sq      F Pr(>F)
## 1    202 27.377                           
## 2    198 27.212  4   0.16516 0.3004 0.8774
## Analysis of Variance Table
## 
## Response: resid
##            Df  Sum Sq Mean Sq F value    Pr(>F)    
## Incubator   1  1.6417 1.64168 12.1131 0.0006134 ***
## Flusher     3  0.8050 0.26833  1.9799 0.1181883    
## Side        1  0.3948 0.39479  2.9130 0.0894068 .  
## Residuals 202 27.3769 0.13553                      
## ---
## Signif. codes:  0 '***' 0.001 '**' 0.01 '*' 0.05 '.' 0.1 ' ' 1
## 
## Call:
## lm(formula = resid ~ Incubator + Flusher + Side, data = temp, 
##     contrasts = list(Incubator = contr.sum, Flusher = contr.sum))
## 
## Residuals:
##      Min       1Q   Median       3Q      Max 
## -1.09783 -0.23782  0.00523  0.24239  0.97274 
## 
## Coefficients:
##             Estimate Std. Error t value Pr(>|t|)    
## (Intercept)  0.03251    0.04028   0.807 0.420577    
## Incubator1   0.09020    0.02562   3.521 0.000532 ***
## Flusher1    -0.07805    0.04882  -1.599 0.111425    
## Flusher2     0.09075    0.04882   1.859 0.064487 .  
## Flusher3     0.03719    0.04102   0.907 0.365599    
## SideR       -0.08713    0.05105  -1.707 0.089407 .  
## ---
## Signif. codes:  0 '***' 0.001 '**' 0.01 '*' 0.05 '.' 0.1 ' ' 1
## 
## Residual standard error: 0.3681 on 202 degrees of freedom
## Multiple R-squared:  0.09403,    Adjusted R-squared:  0.07161 
## F-statistic: 4.193 on 5 and 202 DF,  p-value: 0.0012
## 
## Analysis of Variance Table
## 
## Response: resid
##              Df  Sum Sq Mean Sq F value  Pr(>F)  
## ControlUnit   5  1.8483 0.36965  2.6897 0.02238 *
## Flusher       3  0.7636 0.25453  1.8520 0.13904  
## Side          1  0.3948 0.39479  2.8726 0.09167 .
## Residuals   198 27.2118 0.13743                  
## ---
## Signif. codes:  0 '***' 0.001 '**' 0.01 '*' 0.05 '.' 0.1 ' ' 1
## 
## Call:
## lm(formula = resid ~ ControlUnit + Flusher + Side, data = temp, 
##     contrasts = list(ControlUnit = contr.sum, Flusher = contr.sum))
## 
## Residuals:
##      Min       1Q   Median       3Q      Max 
## -1.08979 -0.24583  0.00171  0.25260  1.00626 
## 
## Coefficients:
##              Estimate Std. Error t value Pr(>|t|)   
## (Intercept)   0.02716    0.04278   0.635   0.5262   
## ControlUnit1 -0.08476    0.07245  -1.170   0.2435   
## ControlUnit2 -0.08378    0.05439  -1.540   0.1251   
## ControlUnit3 -0.09157    0.07357  -1.245   0.2147   
## ControlUnit4  0.05999    0.07357   0.815   0.4158   
## ControlUnit5  0.14205    0.05439   2.612   0.0097 **
## Flusher1     -0.07937    0.05206  -1.525   0.1289   
## Flusher2      0.08943    0.05206   1.718   0.0874 . 
## Flusher3      0.04115    0.06592   0.624   0.5332   
## SideR        -0.08713    0.05141  -1.695   0.0917 . 
## ---
## Signif. codes:  0 '***' 0.001 '**' 0.01 '*' 0.05 '.' 0.1 ' ' 1
## 
## Residual standard error: 0.3707 on 198 degrees of freedom
## Multiple R-squared:  0.0995, Adjusted R-squared:  0.05856 
## F-statistic: 2.431 on 9 and 198 DF,  p-value: 0.01216
```

Test for effect of flusher, conditional on control unit, for Late
erythroid.

```
for (j in c("BM-1 Floating", "BM-1 Scaffold",
            "BM-2 Floating", "BM-3 Floating")) {
  print("")
  print("")
  print(j)
  testSet <- full.toModel[full.toModel$StudyFraction == j, ] ## grab data
  testSet$value <- testSet$value / 100.0 ## reduce to proportion
  testSet$Condition <- with(testSet, paste0(DoseName, "-", Day))
  ## replace zeroes with minimal alternative value
  testSet.noZeroes <- testSet
  sel <- testSet.noZeroes$value == 0
  testSet.noZeroes[sel, "value"] <- min(testSet.noZeroes[!sel, "value"])

  ## regress out the biology
  model3 <- lm(logit(value) ~ Condition * Parameter, data = testSet.noZeroes)

  temp <- testSet.noZeroes
  temp$resid <- resid(model3)
  temp <- temp[temp$Parameter == "LateErythroid_pct", ]

  p <- ggplot(temp, aes(x = ControlUnit, y = resid, col = Flusher)) +
    # facet_grid(. ~ Incubator) +
    geom_point(position = position_jitter(w = 0.1, h = 0)) +
    scale_color_manual(values = cbbPalette) +
    ylim(-2, 2)

  print(p)

  p <- ggplot(temp, aes(x = ControlUnit, y = resid, fill = Flusher)) +
    # facet_grid(. ~ Incubator) +
    geom_boxplot() +
    scale_color_manual(values = cbbPalette) +
    ylim(-2, 2)
  print(p)

  model_incubator <- lm(resid ~ Incubator + Flusher + Side, data = temp,
                        contrasts = list(Incubator = contr.sum, Flusher = contr.sum))
  model_cu <- lm(resid ~ ControlUnit + Flusher + Side, data = temp,
                 contrasts = list(ControlUnit = contr.sum, Flusher = contr.sum))

  print(anova(model_incubator, model_cu))
  print(anova(model_incubator))
  print(summary(model_incubator))
  print(anova(model_cu))
  print(summary(model_cu))
}
```

```
## [1] ""
## [1] ""
## [1] "BM-1 Floating"
```

```
## Warning: Removed 2 rows containing missing values (`geom_point()`).
```

```
## Warning: Removed 2 rows containing non-finite values (`stat_boxplot()`).
```

```
## Analysis of Variance Table
## 
## Model 1: resid ~ Incubator + Flusher + Side
## Model 2: resid ~ ControlUnit + Flusher + Side
##   Res.Df    RSS Df Sum of Sq      F Pr(>F)
## 1     38 40.905                           
## 2     34 38.770  4    2.1343 0.4679 0.7588
## Analysis of Variance Table
## 
## Response: resid
##           Df Sum Sq Mean Sq F value Pr(>F)
## Incubator  1  2.281 2.28052  2.1186 0.1537
## Flusher    1  1.445 1.44456  1.3420 0.2539
## Side       1  0.517 0.51703  0.4803 0.4925
## Residuals 38 40.905 1.07644               
## 
## Call:
## lm(formula = resid ~ Incubator + Flusher + Side, data = temp, 
##     contrasts = list(Incubator = contr.sum, Flusher = contr.sum))
## 
## Residuals:
##      Min       1Q   Median       3Q      Max 
## -2.61501 -0.45002  0.05413  0.68994  1.85615 
## 
## Coefficients:
##             Estimate Std. Error t value Pr(>|t|)
## (Intercept)  -0.1200     0.2267  -0.529    0.600
## Incubator1   -0.2216     0.1611  -1.376    0.177
## Flusher1     -0.1753     0.1618  -1.083    0.286
## SideR         0.2233     0.3222   0.693    0.492
## 
## Residual standard error: 1.038 on 38 degrees of freedom
## Multiple R-squared:  0.09396,    Adjusted R-squared:  0.02243 
## F-statistic: 1.314 on 3 and 38 DF,  p-value: 0.2841
## 
## Analysis of Variance Table
## 
## Response: resid
##             Df Sum Sq Mean Sq F value Pr(>F)
## ControlUnit  5  3.699 0.73983  0.6488 0.6643
## Flusher      1  2.268 2.26774  1.9887 0.1676
## Side         1  0.409 0.40949  0.3591 0.5530
## Residuals   34 38.770 1.14031               
## 
## Call:
## lm(formula = resid ~ ControlUnit + Flusher + Side, data = temp, 
##     contrasts = list(ControlUnit = contr.sum, Flusher = contr.sum))
## 
## Residuals:
##      Min       1Q   Median       3Q      Max 
## -2.67380 -0.42580 -0.00818  0.73362  1.85328 
## 
## Coefficients:
##              Estimate Std. Error t value Pr(>|t|)
## (Intercept)  -0.12635    0.23425  -0.539    0.593
## ControlUnit1  0.38577    0.38068   1.013    0.318
## ControlUnit2  0.05876    0.39573   0.148    0.883
## ControlUnit3  0.21903    0.35905   0.610    0.546
## ControlUnit4 -0.17461    0.36588  -0.477    0.636
## ControlUnit5 -0.63893    0.40805  -1.566    0.127
## Flusher1     -0.25008    0.18615  -1.343    0.188
## SideR         0.19915    0.33233   0.599    0.553
## 
## Residual standard error: 1.068 on 34 degrees of freedom
## Multiple R-squared:  0.1412, Adjusted R-squared:  -0.03557 
## F-statistic: 0.7988 on 7 and 34 DF,  p-value: 0.5938
## 
## [1] ""
## [1] ""
## [1] "BM-1 Scaffold"
```

```
## Warning: Removed 2 rows containing missing values (`geom_point()`).
```

```
## Warning: Removed 2 rows containing non-finite values (`stat_boxplot()`).
```

```
## Analysis of Variance Table
## 
## Model 1: resid ~ Incubator + Flusher + Side
## Model 2: resid ~ ControlUnit + Flusher + Side
##   Res.Df    RSS Df Sum of Sq      F Pr(>F)
## 1     38 40.461                           
## 2     34 38.521  4      1.94 0.4281 0.7873
## Analysis of Variance Table
## 
## Response: resid
##           Df Sum Sq Mean Sq F value Pr(>F)
## Incubator  1  0.170 0.17024  0.1599 0.6915
## Flusher    1  0.896 0.89582  0.8413 0.3648
## Side       1  0.044 0.04398  0.0413 0.8400
## Residuals 38 40.461 1.06475               
## 
## Call:
## lm(formula = resid ~ Incubator + Flusher + Side, data = temp, 
##     contrasts = list(Incubator = contr.sum, Flusher = contr.sum))
## 
## Residuals:
##      Min       1Q   Median       3Q      Max 
## -2.21391 -0.63116  0.03091  0.66584  2.20248 
## 
## Coefficients:
##             Estimate Std. Error t value Pr(>|t|)
## (Intercept) -0.03940    0.22546  -0.175    0.862
## Incubator1  -0.05154    0.16021  -0.322    0.749
## Flusher1    -0.14359    0.16095  -0.892    0.378
## SideR        0.06512    0.32042   0.203    0.840
## 
## Residual standard error: 1.032 on 38 degrees of freedom
## Multiple R-squared:  0.0267, Adjusted R-squared:  -0.05014 
## F-statistic: 0.3475 on 3 and 38 DF,  p-value: 0.7911
## 
## Analysis of Variance Table
## 
## Response: resid
##             Df Sum Sq Mean Sq F value Pr(>F)
## ControlUnit  5  1.791 0.35818  0.3161 0.8998
## Flusher      1  1.236 1.23618  1.0911 0.3036
## Side         1  0.023 0.02289  0.0202 0.8878
## Residuals   34 38.521 1.13296               
## 
## Call:
## lm(formula = resid ~ ControlUnit + Flusher + Side, data = temp, 
##     contrasts = list(ControlUnit = contr.sum, Flusher = contr.sum))
## 
## Residuals:
##     Min      1Q  Median      3Q     Max 
## -2.1099 -0.5152 -0.1574  0.6906  1.9722 
## 
## Coefficients:
##              Estimate Std. Error t value Pr(>|t|)
## (Intercept)  -0.05687    0.23350  -0.244    0.809
## ControlUnit1  0.02966    0.37945   0.078    0.938
## ControlUnit2 -0.16747    0.39446  -0.425    0.674
## ControlUnit3  0.28587    0.35789   0.799    0.430
## ControlUnit4  0.14957    0.36470   0.410    0.684
## ControlUnit5 -0.41641    0.40673  -1.024    0.313
## Flusher1     -0.19022    0.18555  -1.025    0.313
## SideR         0.04709    0.33126   0.142    0.888
## 
## Residual standard error: 1.064 on 34 degrees of freedom
## Multiple R-squared:  0.07337,    Adjusted R-squared:  -0.1174 
## F-statistic: 0.3846 on 7 and 34 DF,  p-value: 0.9049
## 
## [1] ""
## [1] ""
## [1] "BM-2 Floating"
```

```
## Analysis of Variance Table
## 
## Model 1: resid ~ Incubator + Flusher + Side
## Model 2: resid ~ ControlUnit + Flusher + Side
##   Res.Df    RSS Df Sum of Sq      F    Pr(>F)    
## 1     85 38.385                                  
## 2     81 20.604  4     17.78 17.475 2.253e-10 ***
## ---
## Signif. codes:  0 '***' 0.001 '**' 0.01 '*' 0.05 '.' 0.1 ' ' 1
## Analysis of Variance Table
## 
## Response: resid
##           Df Sum Sq Mean Sq F value  Pr(>F)  
## Incubator  1  1.533 1.53284  3.3943 0.06891 .
## Flusher    2  0.594 0.29711  0.6579 0.52054  
## Side       1  0.053 0.05264  0.1166 0.73363  
## Residuals 85 38.385 0.45159                  
## ---
## Signif. codes:  0 '***' 0.001 '**' 0.01 '*' 0.05 '.' 0.1 ' ' 1
## 
## Call:
## lm(formula = resid ~ Incubator + Flusher + Side, data = temp, 
##     contrasts = list(Incubator = contr.sum, Flusher = contr.sum))
## 
## Residuals:
##      Min       1Q   Median       3Q      Max 
## -1.70069 -0.38171 -0.05719  0.44130  1.46895 
## 
## Coefficients:
##             Estimate Std. Error t value Pr(>|t|)
## (Intercept) -0.05517    0.11182  -0.493    0.623
## Incubator1  -0.12008    0.07551  -1.590    0.116
## Flusher1    -0.11557    0.16696  -0.692    0.491
## Flusher2    -0.01128    0.11256  -0.100    0.920
## SideR        0.04867    0.14255   0.341    0.734
## 
## Residual standard error: 0.672 on 85 degrees of freedom
## Multiple R-squared:  0.05373,    Adjusted R-squared:  0.009204 
## F-statistic: 1.207 on 4 and 85 DF,  p-value: 0.314
## 
## Analysis of Variance Table
## 
## Response: resid
##             Df  Sum Sq Mean Sq F value    Pr(>F)    
## ControlUnit  5 19.3606  3.8721  15.222 1.582e-10 ***
## Flusher      2  0.5942  0.2971   1.168    0.3162    
## Side         1  0.0053  0.0053   0.021    0.8852    
## Residuals   81 20.6044  0.2544                      
## ---
## Signif. codes:  0 '***' 0.001 '**' 0.01 '*' 0.05 '.' 0.1 ' ' 1
## 
## Call:
## lm(formula = resid ~ ControlUnit + Flusher + Side, data = temp, 
##     contrasts = list(ControlUnit = contr.sum, Flusher = contr.sum))
## 
## Residuals:
##      Min       1Q   Median       3Q      Max 
## -1.19923 -0.29161  0.00795  0.31888  1.68474 
## 
## Coefficients:
##              Estimate Std. Error t value Pr(>|t|)    
## (Intercept)   0.09863    0.08718   1.131    0.261    
## ControlUnit1 -0.07175    0.14350  -0.500    0.618    
## ControlUnit2  0.05928    0.11007   0.539    0.592    
## ControlUnit3 -0.04666    0.12285  -0.380    0.705    
## ControlUnit4  0.72680    0.14350   5.065 2.52e-06 ***
## ControlUnit5 -0.77534    0.10213  -7.591 4.77e-11 ***
## Flusher1     -0.11557    0.12531  -0.922    0.359    
## Flusher2     -0.01128    0.08448  -0.134    0.894    
## SideR        -0.01563    0.10794  -0.145    0.885    
## ---
## Signif. codes:  0 '***' 0.001 '**' 0.01 '*' 0.05 '.' 0.1 ' ' 1
## 
## Residual standard error: 0.5044 on 81 degrees of freedom
## Multiple R-squared:  0.4921, Adjusted R-squared:  0.4419 
## F-statistic: 9.808 on 8 and 81 DF,  p-value: 2e-09
## 
## [1] ""
## [1] ""
## [1] "BM-3 Floating"
```

```
## Warning: Removed 11 rows containing missing values (`geom_point()`).
```

```
## Warning: Removed 11 rows containing non-finite values (`stat_boxplot()`).
```

```
## Analysis of Variance Table
## 
## Model 1: resid ~ Incubator + Flusher + Side
## Model 2: resid ~ ControlUnit + Flusher + Side
##   Res.Df    RSS Df Sum of Sq      F    Pr(>F)    
## 1    202 175.80                                  
## 2    198 149.56  4    26.235 8.6829 1.775e-06 ***
## ---
## Signif. codes:  0 '***' 0.001 '**' 0.01 '*' 0.05 '.' 0.1 ' ' 1
## Analysis of Variance Table
## 
## Response: resid
##            Df  Sum Sq Mean Sq F value  Pr(>F)  
## Incubator   1   0.466  0.4657  0.5350 0.46534  
## Flusher     3   0.951  0.3170  0.3643 0.77887  
## Side        1   3.967  3.9675  4.5587 0.03396 *
## Residuals 202 175.800  0.8703                  
## ---
## Signif. codes:  0 '***' 0.001 '**' 0.01 '*' 0.05 '.' 0.1 ' ' 1
## 
## Call:
## lm(formula = resid ~ Incubator + Flusher + Side, data = temp, 
##     contrasts = list(Incubator = contr.sum, Flusher = contr.sum))
## 
## Residuals:
##     Min      1Q  Median      3Q     Max 
## -4.4966 -0.3719  0.0364  0.3742  3.6376 
## 
## Coefficients:
##             Estimate Std. Error t value Pr(>|t|)  
## (Intercept) -0.13812    0.10208  -1.353    0.178  
## Incubator1  -0.04419    0.06492  -0.681    0.497  
## Flusher1    -0.08488    0.12370  -0.686    0.493  
## Flusher2    -0.06365    0.12370  -0.515    0.607  
## Flusher3     0.05281    0.10393   0.508    0.612  
## SideR        0.27622    0.12937   2.135    0.034 *
## ---
## Signif. codes:  0 '***' 0.001 '**' 0.01 '*' 0.05 '.' 0.1 ' ' 1
## 
## Residual standard error: 0.9329 on 202 degrees of freedom
## Multiple R-squared:  0.02972,    Adjusted R-squared:  0.0057 
## F-statistic: 1.237 on 5 and 202 DF,  p-value: 0.2929
## 
## Analysis of Variance Table
## 
## Response: resid
##              Df  Sum Sq Mean Sq F value    Pr(>F)    
## ControlUnit   5  27.031  5.4061  7.1569 3.531e-06 ***
## Flusher       3   0.621  0.2071  0.2742   0.84396    
## Side          1   3.967  3.9675  5.2523   0.02297 *  
## Residuals   198 149.565  0.7554                      
## ---
## Signif. codes:  0 '***' 0.001 '**' 0.01 '*' 0.05 '.' 0.1 ' ' 1
## 
## Call:
## lm(formula = resid ~ ControlUnit + Flusher + Side, data = temp, 
##     contrasts = list(ControlUnit = contr.sum, Flusher = contr.sum))
## 
## Residuals:
##     Min      1Q  Median      3Q     Max 
## -3.9912 -0.3670  0.0155  0.3898  3.3194 
## 
## Coefficients:
##              Estimate Std. Error t value Pr(>|t|)    
## (Intercept)  -0.08741    0.10029  -0.872 0.384499    
## ControlUnit1 -0.22375    0.16986  -1.317 0.189259    
## ControlUnit2  0.25916    0.12752   2.032 0.043458 *  
## ControlUnit3  0.10398    0.17248   0.603 0.547311    
## ControlUnit4 -0.65283    0.17248  -3.785 0.000204 ***
## ControlUnit5 -0.12311    0.12752  -0.965 0.335499    
## Flusher1     -0.03239    0.12204  -0.265 0.790973    
## Flusher2     -0.01116    0.12204  -0.091 0.927230    
## Flusher3     -0.10465    0.15455  -0.677 0.499120    
## SideR         0.27622    0.12053   2.292 0.022970 *  
## ---
## Signif. codes:  0 '***' 0.001 '**' 0.01 '*' 0.05 '.' 0.1 ' ' 1
## 
## Residual standard error: 0.8691 on 198 degrees of freedom
## Multiple R-squared:  0.1745, Adjusted R-squared:  0.137 
## F-statistic: 4.651 on 9 and 198 DF,  p-value: 1.343e-05
```

Figure 6F:

```
set.seed(94)

testSet <- full.toModel[full.toModel$StudyFraction == "BM-2 Floating", ] ## grab data
names(testSet)[names(testSet) == "Flusher"] <- "Operator"
testSet$value <- testSet$value / 100.0 ## reduce to proportion
testSet$Condition <- with(testSet, paste0(DoseName, "-", Day))
## replace zeroes with minimal alternative value
testSet.noZeroes <- testSet
sel <- testSet.noZeroes$value == 0
testSet.noZeroes[sel, "value"] <- min(testSet.noZeroes[!sel, "value"])

## regress out the biology
model3 <- lm(logit(value) ~ Condition * Parameter, data = testSet.noZeroes)

temp <- testSet.noZeroes
temp$resid <- resid(model3)
temp <- temp[temp$Parameter == "LateErythroid_pct", ]

p <- ggplot(temp, aes(x = ControlUnit, y = resid, col = Operator)) +
  # facet_grid(. ~ Incubator) +
  geom_point(position = position_jitter(w = 0.1, h = 0), size = 3) +
  scale_color_manual(values = cbbPalette) +
  ylim(-2, 2) +
  theme_bw() +
  theme(
      plot.title = element_text(size=20),
      legend.title = element_text(size=20),
      legend.text = element_text(size=20),
      legend.key.width = unit(1,"cm"),
      legend.key.height = unit(1,"cm"),
      axis.text=element_text(size=16),
      axis.title=element_text(size=16)
  )

print(p)
```

```
pander(sessionInfo())
```

**R version 4.2.1 (2022-06-23 ucrt)**

**Platform:** x86\_64-w64-mingw32/x64 (64-bit)

**locale:** *LC\_COLLATE=English\_United
Kingdom.utf8*, *LC\_CTYPE=English\_United Kingdom.utf8*,
*LC\_MONETARY=English\_United Kingdom.utf8*, *LC\_NUMERIC=C*
and *LC\_TIME=English\_United Kingdom.utf8*

**attached base packages:** *grid*,
*stats*, *graphics*, *grDevices*, *utils*,
*datasets*, *methods* and *base*

**other attached packages:**
*naturalsort(v.0.1.3)*, *MASS(v.7.3-57)*,
*here(v.1.0.1)*, *pander(v.0.6.5)*,
*lmerTest(v.3.1-3)*, *lme4(v.1.1-30)*,
*Matrix(v.1.5-1)*, *tidyr(v.1.2.1)*,
*dplyr(v.1.0.10)*, *data.table(v.1.14.2)*,
*magrittr(v.2.0.3)*, *ggbiplot(v.0.55)*,
*scales(v.1.2.1)*, *plyr(v.1.8.7)* and
*ggplot2(v.3.4.0)*

**loaded via a namespace (and not attached):**
*tidyselect(v.1.2.0)*, *xfun(v.0.31)*,
*bslib(v.0.4.0)*, *purrr(v.0.3.5)*,
*splines(v.4.2.1)*, *lattice(v.0.20-45)*,
*colorspace(v.2.0-3)*, *vctrs(v.0.5.1)*,
*generics(v.0.1.3)*, *htmltools(v.0.5.3)*,
*yaml(v.2.3.5)*, *utf8(v.1.2.2)*, *rlang(v.1.0.6)*,
*nloptr(v.2.0.3)*, *jquerylib(v.0.1.4)*,
*pillar(v.1.8.1)*, *glue(v.1.6.2)*,
*withr(v.2.5.0)*, *DBI(v.1.1.3)*,
*lifecycle(v.1.0.3)*, *stringr(v.1.4.1)*,
*munsell(v.0.5.0)*, *gtable(v.0.3.1)*,
*evaluate(v.0.17)*, *labeling(v.0.4.2)*,
*knitr(v.1.40)*, *fastmap(v.1.1.0)*,
*fansi(v.1.0.3)*, *highr(v.0.9)*, *Rcpp(v.1.0.9)*,
*cachem(v.1.0.6)*, *jsonlite(v.1.8.2)*,
*farver(v.2.1.1)*, *digest(v.0.6.29)*,
*stringi(v.1.7.8)*, *rprojroot(v.2.0.3)*,
*numDeriv(v.2016.8-1.1)*, *cli(v.3.4.1)*,
*tools(v.4.2.1)*, *sass(v.0.4.2)*,
*tibble(v.3.1.8)*, *pkgconfig(v.2.0.3)*,
*ellipsis(v.0.3.2)*, *minqa(v.1.2.4)*,
*assertthat(v.0.2.1)*, *rmarkdown(v.2.17)*,
*rstudioapi(v.0.14)*, *R6(v.2.5.1)*,
*boot(v.1.3-28)*, *nlme(v.3.1-157)* and
*compiler(v.4.2.1)*
