# Supplementary material for: Optimal experimental design for efficient toxicity testing in microphysiological systems: A bone marrow application
Source: Front Pharmacol. 2023 Mar 31;14:1142581. doi: 10.3389/fphar.2023.1142581 (PMC10103791; doi:10.3389/fphar.2023.1142581)
Supplement: Supplementary file 2 [file DataSheet1.zip › Data package/Reports/mixedModel_PAPER_BM1.html]

Bone Marrow MPS - compare 2018-04/05


Code 

- Show All Code
- Hide All Code

# Bone Marrow MPS - compare 2018-04/05

#### Statistician: Jonathan Cairns

#### 5 April 2019

Read in data:

```
source("createFull.R")
```

```
## Warning: package 'naturalsort' was built under R version 4.1.2
```

```
##create full.gathered
interestingCols <- c("EarlyErythroid", "LateErythroid", "EarlyMyeloid", "ltHSC", "Platelets", "LineageDiffd", "LateGranulocytes")
metaDataCols <- c(colnames(full)[1:10], "Study", "Group", "Day", "StudyFraction")

##specify the columns of interest


mySel <- c(colnames(full) %>% grep(pattern = "CD"), which(colnames(full) %in% c(interestingCols, paste0(interestingCols, "_pct"))))

full.gathered <- full %>% gather_(
  key_col      = 'Parameter',
  value_col    = 'value',
  gather_cols  = colnames(full)[mySel])

forPCA <- as.data.frame(full)[,c(metaDataCols, colnames(full) %>% grep(pattern = "CD", value=TRUE), paste0(c(interestingCols, "DeadCells"), "_pct"))]
```

## Mixed model

Explore mixed models - unfortunately models are rank-deficient.

```
lc1 <- lmerControl(optimizer = "Nelder_Mead")

testC <- data.table(full.gathered)[Parameter == "LateErythroid" & Study == "BM-1",]

modelC_Unit_Flush <- lmer(log(value + 1) ~ factor(Dose)*Day + (1 | Incubator) + (1 | Incubator:ControlUnit) + (1 | Flusher) + (1 | Fraction), data = testC, control = lc1)
```

```
## fixed-effect model matrix is rank deficient so dropping 2 columns / coefficients
```

```
## boundary (singular) fit: see ?isSingular
```

```
## fixed-effect model matrix is rank deficient so dropping 2 columns / coefficients
```

```
modelC_Unit <- lmer(log(value + 1) ~ factor(Dose)*Day + (1 | Incubator) + (1 | Incubator:ControlUnit) + (1 | Fraction), data = testC, control = lc1)
```

```
## fixed-effect model matrix is rank deficient so dropping 2 columns / coefficients
## fixed-effect model matrix is rank deficient so dropping 2 columns / coefficients
```

```
modelC_Flush <- lmer(log(value + 1) ~ factor(Dose)*Day + (1 | Incubator) + (1 | Flusher) + (1 | Fraction), data = testC, control = lc1)
```

```
## fixed-effect model matrix is rank deficient so dropping 2 columns / coefficients
## fixed-effect model matrix is rank deficient so dropping 2 columns / coefficients
```

```
modelC_Fraction <- lmer(log(value + 1) ~ factor(Dose)*Day + (1 | Incubator) + (1 | Fraction), data = testC, control = lc1)
```

```
## fixed-effect model matrix is rank deficient so dropping 2 columns / coefficients
## fixed-effect model matrix is rank deficient so dropping 2 columns / coefficients
```

```
summary(modelC_Unit_Flush)
```

```
## Linear mixed model fit by REML. t-tests use Satterthwaite's method [
## lmerModLmerTest]
## Formula: log(value + 1) ~ factor(Dose) * Day + (1 | Incubator) + (1 |  
##     Incubator:ControlUnit) + (1 | Flusher) + (1 | Fraction)
##    Data: testC
## Control: lc1
## 
## REML criterion at convergence: 281.7
## 
## Scaled residuals: 
##      Min       1Q   Median       3Q      Max 
## -2.14804 -0.71923 -0.06547  0.79003  2.12482 
## 
## Random effects:
##  Groups                Name        Variance Std.Dev.
##  Incubator:ControlUnit (Intercept) 0.00000  0.0000  
##  Fraction              (Intercept) 0.83567  0.9142  
##  Flusher               (Intercept) 0.07921  0.2814  
##  Incubator             (Intercept) 0.06525  0.2554  
##  Residual                          1.69567  1.3022  
## Number of obs: 84, groups:  
## Incubator:ControlUnit, 6; Fraction, 2; Flusher, 2; Incubator, 2
## 
## Fixed effects:
##                       Estimate Std. Error      df t value Pr(>|t|)    
## (Intercept)             7.1450     0.7946  2.0037   8.992   0.0121 *  
## factor(Dose)1          -2.6616     0.5316 73.8428  -5.007 3.65e-06 ***
## factor(Dose)10         -5.5348     0.5330 74.2447 -10.384 4.11e-16 ***
## Dayd14                  0.7927     0.5329 74.3014   1.487   0.1412    
## Dayd28                  0.6487     0.5316 73.8428   1.220   0.2263    
## factor(Dose)1:Dayd14    1.1379     0.7556 74.6452   1.506   0.1363    
## factor(Dose)10:Dayd14  -0.8247     0.7539 74.3483  -1.094   0.2775    
## ---
## Signif. codes:  0 '***' 0.001 '**' 0.01 '*' 0.05 '.' 0.1 ' ' 1
## 
## Correlation of Fixed Effects:
##             (Intr) fc(D)1 fc(D)10 Dayd14 Dayd28 f(D)1:
## factor(Ds)1  0.000                                    
## factr(Ds)10  0.000  0.499                             
## Dayd14      -0.334  0.000  0.000                      
## Dayd28      -0.335 -0.500 -0.499   0.499              
## fct(D)1:D14  0.000 -0.704 -0.351  -0.358  0.352       
## fc(D)10:D14  0.000 -0.353 -0.707  -0.355  0.353  0.501
## fit warnings:
## fixed-effect model matrix is rank deficient so dropping 2 columns / coefficients
## optimizer (Nelder_Mead) convergence code: 0 (OK)
## boundary (singular) fit: see ?isSingular
```

```
summary(modelC_Unit)
```

```
## Linear mixed model fit by REML. t-tests use Satterthwaite's method [
## lmerModLmerTest]
## Formula: log(value + 1) ~ factor(Dose) * Day + (1 | Incubator) + (1 |  
##     Incubator:ControlUnit) + (1 | Fraction)
##    Data: testC
## Control: lc1
## 
## REML criterion at convergence: 282.5
## 
## Scaled residuals: 
##     Min      1Q  Median      3Q     Max 
## -2.0605 -0.7545 -0.1548  0.7479  2.1826 
## 
## Random effects:
##  Groups                Name        Variance Std.Dev.
##  Incubator:ControlUnit (Intercept) 0.01625  0.1275  
##  Fraction              (Intercept) 0.83480  0.9137  
##  Incubator             (Intercept) 0.04514  0.2125  
##  Residual                          1.73129  1.3158  
## Number of obs: 84, groups:  Incubator:ControlUnit, 6; Fraction, 2; Incubator, 2
## 
## Fixed effects:
##                       Estimate Std. Error      df t value Pr(>|t|)    
## (Intercept)             7.1272     0.7672  1.7839   9.290   0.0162 *  
## factor(Dose)1          -2.6792     0.5402 72.0573  -4.960 4.55e-06 ***
## factor(Dose)10         -5.5928     0.5387 74.9683 -10.382 3.70e-16 ***
## Dayd14                  0.8117     0.5434 59.9870   1.494   0.1405    
## Dayd28                  0.6620     0.5387 74.9218   1.229   0.2229    
## factor(Dose)1:Dayd14    1.1794     0.7709 56.1690   1.530   0.1317    
## factor(Dose)10:Dayd14  -0.7680     0.7641 73.9022  -1.005   0.3181    
## ---
## Signif. codes:  0 '***' 0.001 '**' 0.01 '*' 0.05 '.' 0.1 ' ' 1
## 
## Correlation of Fixed Effects:
##             (Intr) fc(D)1 fc(D)10 Dayd14 Dayd28 f(D)1:
## factor(Ds)1  0.001                                    
## factr(Ds)10 -0.002  0.501                             
## Dayd14      -0.353  0.002  0.007                      
## Dayd28      -0.352 -0.501 -0.497   0.498              
## fct(D)1:D14  0.001 -0.707 -0.357  -0.359  0.351       
## fc(D)10:D14  0.004 -0.356 -0.708  -0.362  0.350  0.506
## fit warnings:
## fixed-effect model matrix is rank deficient so dropping 2 columns / coefficients
```

```
summary(modelC_Flush)
```

```
## Linear mixed model fit by REML. t-tests use Satterthwaite's method [
## lmerModLmerTest]
## Formula: log(value + 1) ~ factor(Dose) * Day + (1 | Incubator) + (1 |  
##     Flusher) + (1 | Fraction)
##    Data: testC
## Control: lc1
## 
## REML criterion at convergence: 281.7
## 
## Scaled residuals: 
##      Min       1Q   Median       3Q      Max 
## -2.14804 -0.71923 -0.06547  0.79003  2.12482 
## 
## Random effects:
##  Groups    Name        Variance Std.Dev.
##  Incubator (Intercept) 0.06525  0.2554  
##  Flusher   (Intercept) 0.07921  0.2814  
##  Fraction  (Intercept) 0.83566  0.9141  
##  Residual              1.69567  1.3022  
## Number of obs: 84, groups:  Incubator, 2; Flusher, 2; Fraction, 2
## 
## Fixed effects:
##                       Estimate Std. Error      df t value Pr(>|t|)    
## (Intercept)             7.1450     0.7946  2.0038   8.992   0.0121 *  
## factor(Dose)1          -2.6616     0.5316 73.8428  -5.007 3.65e-06 ***
## factor(Dose)10         -5.5348     0.5330 74.2447 -10.384 4.11e-16 ***
## Dayd14                  0.7927     0.5329 74.3014   1.487   0.1412    
## Dayd28                  0.6487     0.5316 73.8428   1.220   0.2263    
## factor(Dose)1:Dayd14    1.1379     0.7556 74.6452   1.506   0.1363    
## factor(Dose)10:Dayd14  -0.8247     0.7539 74.3483  -1.094   0.2775    
## ---
## Signif. codes:  0 '***' 0.001 '**' 0.01 '*' 0.05 '.' 0.1 ' ' 1
## 
## Correlation of Fixed Effects:
##             (Intr) fc(D)1 fc(D)10 Dayd14 Dayd28 f(D)1:
## factor(Ds)1  0.000                                    
## factr(Ds)10  0.000  0.499                             
## Dayd14      -0.334  0.000  0.000                      
## Dayd28      -0.335 -0.500 -0.499   0.499              
## fct(D)1:D14  0.000 -0.704 -0.351  -0.358  0.352       
## fc(D)10:D14  0.000 -0.353 -0.707  -0.355  0.353  0.501
## fit warnings:
## fixed-effect model matrix is rank deficient so dropping 2 columns / coefficients
```

```
summary(modelC_Fraction)
```

```
## Linear mixed model fit by REML. t-tests use Satterthwaite's method [
## lmerModLmerTest]
## Formula: log(value + 1) ~ factor(Dose) * Day + (1 | Incubator) + (1 |  
##     Fraction)
##    Data: testC
## Control: lc1
## 
## REML criterion at convergence: 282.5
## 
## Scaled residuals: 
##     Min      1Q  Median      3Q     Max 
## -2.0608 -0.7442 -0.1454  0.7406  2.1987 
## 
## Random effects:
##  Groups    Name        Variance Std.Dev.
##  Incubator (Intercept) 0.04969  0.2229  
##  Fraction  (Intercept) 0.83455  0.9135  
##  Residual              1.74212  1.3199  
## Number of obs: 84, groups:  Incubator, 2; Fraction, 2
## 
## Fixed effects:
##                       Estimate Std. Error      df t value Pr(>|t|)    
## (Intercept)             7.1450     0.7664  1.7767   9.323   0.0163 *  
## factor(Dose)1          -2.6616     0.5388 75.0000  -4.939 4.63e-06 ***
## factor(Dose)10         -5.5886     0.5388 75.0000 -10.371 3.85e-16 ***
## Dayd14                  0.7842     0.5400 75.4885   1.452   0.1506    
## Dayd28                  0.6487     0.5388 75.0000   1.204   0.2324    
## factor(Dose)1:Dayd14    1.1548     0.7654 75.8147   1.509   0.1355    
## factor(Dose)10:Dayd14  -0.7625     0.7629 75.2645  -0.999   0.3208    
## ---
## Signif. codes:  0 '***' 0.001 '**' 0.01 '*' 0.05 '.' 0.1 ' ' 1
## 
## Correlation of Fixed Effects:
##             (Intr) fc(D)1 fc(D)10 Dayd14 Dayd28 f(D)1:
## factor(Ds)1  0.000                                    
## factr(Ds)10  0.000  0.500                             
## Dayd14      -0.351  0.000  0.000                      
## Dayd28      -0.352 -0.500 -0.500   0.499              
## fct(D)1:D14  0.000 -0.704 -0.352  -0.357  0.352       
## fc(D)10:D14  0.000 -0.353 -0.706  -0.355  0.353  0.502
## fit warnings:
## fixed-effect model matrix is rank deficient so dropping 2 columns / coefficients
```

```
BIC(modelC_Unit_Flush)
```

```
## [1] 334.8335
```

```
BIC(modelC_Unit)
```

```
## [1] 331.1927
```

```
BIC(modelC_Flush)
```

```
## [1] 330.4027
```

```
BIC(modelC_Fraction)
```

```
## [1] 326.7824
```

```
anova(modelC_Unit_Flush, modelC_Unit, modelC_Flush, modelC_Fraction)
```

```
## refitting model(s) with ML (instead of REML)
```

```
## Data: testC
## Models:
## modelC_Fraction: log(value + 1) ~ factor(Dose) * Day + (1 | Incubator) + (1 | Fraction)
## modelC_Unit: log(value + 1) ~ factor(Dose) * Day + (1 | Incubator) + (1 | Incubator:ControlUnit) + (1 | Fraction)
## modelC_Flush: log(value + 1) ~ factor(Dose) * Day + (1 | Incubator) + (1 | Flusher) + (1 | Fraction)
## modelC_Unit_Flush: log(value + 1) ~ factor(Dose) * Day + (1 | Incubator) + (1 | Incubator:ControlUnit) + (1 | Flusher) + (1 | Fraction)
##                   npar    AIC    BIC  logLik deviance  Chisq Df Pr(>Chisq)
## modelC_Fraction     10 304.37 328.68 -142.19   284.37                     
## modelC_Unit         11 306.37 333.11 -142.18   284.37 0.0082  1      0.928
## modelC_Flush        11 305.56 332.30 -141.78   283.56 0.8012  0           
## modelC_Unit_Flush   12 307.56 336.73 -141.78   283.56 0.0000  1      1.000
```

Decomposition of variance across endpoints

```
myEndpoints <- c("EarlyErythroid", "EarlyMyeloid", "LateErythroid", "ltHSC", "Platelets")

plotVariance <- function(x)
{
  mySDs <- as.data.frame(VarCorr(x))
  mySDs$pct <- mySDs$vcov/sum(mySDs$vcov)
  p <- ggplot(mySDs, aes(x="", y=pct, fill=grp)) + geom_bar(width=1, stat="identity") + coord_polar("y", start=0)
}

for(k in myEndpoints)
{  
  print(k)
  
  testC <- data.table(full.gathered)[Parameter == k & StudyFraction == "BM-1 Floating",]
  summary(lm(log(value + 1) ~ Day + factor(Dose):Day, data = testC))
    
  testC <- data.table(full.gathered)[Parameter == k & StudyFraction == "BM-1 Scaffold",]
  summary(lm(log(value + 1) ~ Day + factor(Dose):Day, data = testC))
}
```

```
## [1] "EarlyErythroid"
## [1] "EarlyMyeloid"
## [1] "LateErythroid"
## [1] "ltHSC"
## [1] "Platelets"
```

From all of this, we claim that modelC.bothextra is the best as it has the lowest BIC, and its variance decomposition is consistent with the other models. Output results from BM-1 to file:

```
output_lm <- NULL
output_lm_scaff <- NULL


chew <- function(x, myK, ...)
{
  ##collect information
  temp <- tidy(x)

  temp$param <- myK

  temp$estimate_orig_scale <- exp(temp$estimate)
  #temp[temp$effect == "ran_pars", "estimate_orig_scale"] <- NA

  temp$stars <- sigStars(temp$p.value)
  temp
}
  
for(k in myEndpoints)
{  
  print(k)
  
  testC <- data.table(full.gathered)[Parameter == k & StudyFraction == "BM-1 Floating",]
  
  modelC_Float <- lm(log(value + 1) ~ Day + factor(Dose):Day, data = testC)
  
  testC <- data.table(full.gathered)[Parameter == k & StudyFraction == "BM-1 Scaffold",]
  
  modelC_Scaff <- lm(log(value + 1) ~ Day + factor(Dose):Day, data = testC)
  

  temp <- chew(modelC_Float, k)
  temp$effect <- "fixed"
  temp$group <- NA
  output_lm <- rbind(output_lm, temp)
  output_lm <- output_lm[!is.na(output_lm$estimate),]
  
  temp <- chew(modelC_Scaff, k)
  temp$effect <- "fixed"
  temp$group <- NA
  output_lm_scaff <- rbind(output_lm_scaff, temp)
  output_lm_scaff <- output_lm_scaff[!is.na(output_lm_scaff$estimate),]

}
```

```
## [1] "EarlyErythroid"
## [1] "EarlyMyeloid"
## [1] "LateErythroid"
## [1] "ltHSC"
## [1] "Platelets"
```

```
write.csv(as.data.frame(output_lm), file = "output_BM-1_lm.csv")
write.csv(as.data.frame(output_lm_scaff), file = "output_BM-1_lmscaff.csv")
```

```
pander::pander(sessionInfo())
```

**R version 4.1.0 (2021-05-18)**

**Platform:** x86\_64-w64-mingw32/x64 (64-bit)

**locale:** *LC\_COLLATE=English\_United Kingdom.1252*, *LC\_CTYPE=English\_United Kingdom.1252*, *LC\_MONETARY=English\_United Kingdom.1252*, *LC\_NUMERIC=C* and *LC\_TIME=English\_United Kingdom.1252*

**attached base packages:** *grid*, *stats*, *graphics*, *grDevices*, *utils*, *datasets*, *methods* and *base*

**other attached packages:** *naturalsort(v.0.1.3)*, *here(v.1.0.1)*, *broom.mixed(v.0.2.7)*, *MASS(v.7.3-54)*, *lmerTest(v.3.1-3)*, *lme4(v.1.1-27.1)*, *Matrix(v.1.3-3)*, *tidyr(v.1.1.3)*, *dplyr(v.1.0.7)*, *data.table(v.1.14.0)*, *magrittr(v.2.0.1)*, *pca3d(v.0.10.2)*, *ggbiplot(v.0.55)*, *scales(v.1.1.1)*, *plyr(v.1.8.6)* and *ggplot2(v.3.3.5)*

**loaded via a namespace (and not attached):** *tidyselect(v.1.1.1)*, *xfun(v.0.29)*, *bslib(v.0.3.1)*, *pander(v.0.6.4)*, *purrr(v.0.3.4)*, *splines(v.4.1.0)*, *lattice(v.0.20-44)*, *colorspace(v.2.0-2)*, *vctrs(v.0.3.8)*, *generics(v.0.1.2)*, *htmltools(v.0.5.2)*, *yaml(v.2.2.1)*, *utf8(v.1.2.2)*, *rlang(v.0.4.11)*, *nloptr(v.1.2.2.2)*, *jquerylib(v.0.1.4)*, *pillar(v.1.6.2)*, *glue(v.1.4.2)*, *withr(v.2.4.2)*, *DBI(v.1.1.2)*, *lifecycle(v.1.0.0)*, *stringr(v.1.4.0)*, *munsell(v.0.5.0)*, *gtable(v.0.3.0)*, *htmlwidgets(v.1.5.3)*, *evaluate(v.0.14)*, *knitr(v.1.33)*, *fastmap(v.1.1.0)*, *fansi(v.0.5.0)*, *broom(v.0.7.9)*, *Rcpp(v.1.0.7)*, *backports(v.1.2.1)*, *jsonlite(v.1.7.2)*, *ellipse(v.0.4.2)*, *digest(v.0.6.27)*, *stringi(v.1.7.3)*, *rprojroot(v.2.0.2)*, *numDeriv(v.2016.8-1.1)*, *tools(v.4.1.0)*, *rgl(v.0.107.14)*, *sass(v.0.4.0)*, *tibble(v.3.1.3)*, *crayon(v.1.4.1)*, *pkgconfig(v.2.0.3)*, *ellipsis(v.0.3.2)*, *minqa(v.1.2.4)*, *assertthat(v.0.2.1)*, *rmarkdown(v.2.11)*, *R6(v.2.5.1)*, *boot(v.1.3-28)*, *nlme(v.3.1-152)* and *compiler(v.4.1.0)*
