# Supplementary material for: Optimal experimental design for efficient toxicity testing in microphysiological systems: A bone marrow application
Source: Front Pharmacol. 2023 Mar 31;14:1142581. doi: 10.3389/fphar.2023.1142581 (PMC10103791; doi:10.3389/fphar.2023.1142581)
Supplement: Supplementary file 2 [file DataSheet1.zip › Data package/Reports/mixedModel_PAPER_BM2.html]

Bone Marrow MPS - compare 2018-04/05


Code 

- Show All Code
- Hide All Code

# Bone Marrow MPS - compare 2018-04/05

#### Statistician: Jonathan Cairns

#### 5 April 2019

Read in data:

```
source("createFull.R")
```

```
## Warning: package 'naturalsort' was built under R version 4.1.2
```

```
## Warning: package 'here' was built under R version 4.1.3
```

```
## here() starts at C:/Users/kqxt813/Box Sync/BM-MPS-DoEpaper/Data package
```

```
## 
## Attaching package: 'here'
```

```
## The following object is masked from 'package:plyr':
## 
##     here
```

```
##create full.gathered
interestingCols <- c("EarlyErythroid", "LateErythroid", "EarlyMyeloid", "ltHSC", "Platelets", "LineageDiffd", "LateGranulocytes")
metaDataCols <- c(colnames(full)[1:10], "Study", "Group", "Day", "StudyFraction")

##specify the columns of interest


mySel <- c(colnames(full) %>% grep(pattern = "CD"), which(colnames(full) %in% c(interestingCols, paste0(interestingCols, "_pct"))))

full.gathered <- full %>% gather_(
  key_col      = 'Parameter',
  value_col    = 'value',
  gather_cols  = colnames(full)[mySel])

forPCA <- as.data.frame(full)[,c(metaDataCols, colnames(full) %>% grep(pattern = "CD", value=TRUE), paste0(c(interestingCols, "DeadCells"), "_pct"))]
```

##Mixed model

Final models

```
plotVariance <- function(x)
{
  mySDs <- as.data.frame(VarCorr(x))
  mySDs$pct <- mySDs$vcov/sum(mySDs$vcov)
  p <- ggplot(mySDs, aes(x="", y=pct, fill=grp)) + geom_bar(width=1, stat="identity") + coord_polar("y", start=0)
}


testC <- data.table(full.gathered)[Parameter == "LateErythroid" & StudyFraction == "BM-2 Floating",]

modelC_lm <- lm(log(value + 1) ~ factor(Dose)*Day, data = testC)

summary(modelC_lm)
```

```
## 
## Call:
## lm(formula = log(value + 1) ~ factor(Dose) * Day, data = testC)
## 
## Residuals:
##     Min      1Q  Median      3Q     Max 
## -4.0971 -0.5549  0.1871  0.7250  2.0184 
## 
## Coefficients:
##                        Estimate Std. Error t value Pr(>|t|)    
## (Intercept)            8.849135   0.482620  18.336  < 2e-16 ***
## factor(Dose)1         -0.225172   0.682528  -0.330 0.742389    
## factor(Dose)10        -0.006457   0.682528  -0.009 0.992477    
## Dayd7                  0.940851   0.682528   1.378 0.172153    
## Dayd14                 0.531685   0.682528   0.779 0.438435    
## Dayd21                 0.334462   0.682528   0.490 0.625540    
## Dayd28                -0.954374   0.682528  -1.398 0.166146    
## factor(Dose)1:Dayd7   -1.098873   0.965240  -1.138 0.258558    
## factor(Dose)10:Dayd7  -5.151604   0.965240  -5.337 9.69e-07 ***
## factor(Dose)1:Dayd14  -2.058053   0.965240  -2.132 0.036267 *  
## factor(Dose)10:Dayd14 -6.132887   0.965240  -6.354 1.47e-08 ***
## factor(Dose)1:Dayd21  -1.193769   0.965240  -1.237 0.220035    
## factor(Dose)10:Dayd21 -5.743329   0.965240  -5.950 7.97e-08 ***
## factor(Dose)1:Dayd28  -0.977267   0.965240  -1.012 0.314573    
## factor(Dose)10:Dayd28 -3.791249   0.965240  -3.928 0.000189 ***
## ---
## Signif. codes:  0 '***' 0.001 '**' 0.01 '*' 0.05 '.' 0.1 ' ' 1
## 
## Residual standard error: 1.182 on 75 degrees of freedom
## Multiple R-squared:  0.8039, Adjusted R-squared:  0.7672 
## F-statistic: 21.96 on 14 and 75 DF,  p-value: < 2.2e-16
```

```
modelC.flush <- lmer(log(value + 1) ~ factor(Dose)*Day + (1 | Flusher:Day) + (1 | Incubator) + (1 | Incubator:TubeNo), data = testC)

summary(modelC.flush)
```

```
## Linear mixed model fit by REML. t-tests use Satterthwaite's method [
## lmerModLmerTest]
## Formula: log(value + 1) ~ factor(Dose) * Day + (1 | Flusher:Day) + (1 |  
##     Incubator) + (1 | Incubator:TubeNo)
##    Data: testC
## 
## REML criterion at convergence: 215.9
## 
## Scaled residuals: 
##      Min       1Q   Median       3Q      Max 
## -1.83128 -0.42422  0.06362  0.56531  1.95987 
## 
## Random effects:
##  Groups           Name        Variance  Std.Dev.
##  Incubator:TubeNo (Intercept) 7.376e-01 0.858812
##  Flusher:Day      (Intercept) 4.347e-01 0.659329
##  Incubator        (Intercept) 6.576e-05 0.008109
##  Residual                     3.992e-01 0.631815
## Number of obs: 90, groups:  Incubator:TubeNo, 18; Flusher:Day, 10; Incubator, 2
## 
## Fixed effects:
##                        Estimate Std. Error        df t value Pr(>|t|)    
## (Intercept)            8.849135   0.637847 10.742194  13.873 3.37e-08 ***
## factor(Dose)1         -0.225172   0.615561 26.380010  -0.366 0.717432    
## factor(Dose)10        -0.006457   0.615561 26.380010  -0.010 0.991709    
## Dayd7                  0.940851   0.753511  5.878620   1.249 0.259226    
## Dayd14                 0.531685   0.753511  5.878621   0.706 0.507413    
## Dayd21                 0.334462   0.753511  5.878620   0.444 0.673005    
## Dayd28                -0.954374   0.753511  5.878621  -1.267 0.253177    
## factor(Dose)1:Dayd7   -1.098873   0.515875 56.001557  -2.130 0.037569 *  
## factor(Dose)10:Dayd7  -5.151604   0.515875 56.001557  -9.986 4.81e-14 ***
## factor(Dose)1:Dayd14  -2.058053   0.515875 56.001557  -3.989 0.000194 ***
## factor(Dose)10:Dayd14 -6.132887   0.515875 56.001557 -11.888  < 2e-16 ***
## factor(Dose)1:Dayd21  -1.193769   0.515875 56.001557  -2.314 0.024357 *  
## factor(Dose)10:Dayd21 -5.743329   0.515875 56.001557 -11.133 8.10e-16 ***
## factor(Dose)1:Dayd28  -0.977267   0.515875 56.001557  -1.894 0.063342 .  
## factor(Dose)10:Dayd28 -3.791249   0.515875 56.001557  -7.349 9.16e-10 ***
## ---
## Signif. codes:  0 '***' 0.001 '**' 0.01 '*' 0.05 '.' 0.1 ' ' 1
```

```
## 
## Correlation matrix not shown by default, as p = 15 > 12.
## Use print(x, correlation=TRUE)  or
##     vcov(x)        if you need it
```

```
plot(plotVariance(modelC.flush) + scale_fill_manual(values = cbbPalette))
```

```
modelC.CU <- lmer(log(value + 1) ~ factor(Dose)*Day + (1 | Incubator) + (1 | Incubator:ControlUnit), data = testC)
```

```
## boundary (singular) fit: see ?isSingular
```

```
summary(modelC.CU)
```

```
## Linear mixed model fit by REML. t-tests use Satterthwaite's method [
## lmerModLmerTest]
## Formula: log(value + 1) ~ factor(Dose) * Day + (1 | Incubator) + (1 |  
##     Incubator:ControlUnit)
##    Data: testC
## 
## REML criterion at convergence: 244.4
## 
## Scaled residuals: 
##     Min      1Q  Median      3Q     Max 
## -3.3588 -0.4000  0.1210  0.6124  1.6431 
## 
## Random effects:
##  Groups                Name        Variance Std.Dev.
##  Incubator:ControlUnit (Intercept) 0.3733   0.6110  
##  Incubator             (Intercept) 0.0000   0.0000  
##  Residual                          0.9423   0.9707  
## Number of obs: 90, groups:  Incubator:ControlUnit, 6; Incubator, 2
## 
## Fixed effects:
##                       Estimate Std. Error      df t value Pr(>|t|)    
## (Intercept)             9.0834     0.4759 36.5313  19.087  < 2e-16 ***
## factor(Dose)1          -0.4594     0.5668 71.0788  -0.811   0.4204    
## factor(Dose)10          0.0148     0.5680 71.2944   0.026   0.9793    
## Dayd7                   0.9408     0.5604 70.4674   1.679   0.0976 .  
## Dayd14                  0.5317     0.5604 70.4674   0.949   0.3460    
## Dayd21                  0.3345     0.5604 70.4674   0.597   0.5526    
## Dayd28                 -0.9544     0.5604 70.4674  -1.703   0.0930 .  
## factor(Dose)1:Dayd7    -1.0989     0.7926 70.4674  -1.386   0.1700    
## factor(Dose)10:Dayd7   -5.1516     0.7926 70.4674  -6.500 9.83e-09 ***
## factor(Dose)1:Dayd14   -2.0581     0.7926 70.4674  -2.597   0.0114 *  
## factor(Dose)10:Dayd14  -6.1329     0.7926 70.4674  -7.738 5.34e-11 ***
## factor(Dose)1:Dayd21   -1.1938     0.7926 70.4674  -1.506   0.1365    
## factor(Dose)10:Dayd21  -5.7433     0.7926 70.4674  -7.246 4.29e-10 ***
## factor(Dose)1:Dayd28   -0.9773     0.7926 70.4674  -1.233   0.2217    
## factor(Dose)10:Dayd28  -3.7912     0.7926 70.4674  -4.783 9.16e-06 ***
## ---
## Signif. codes:  0 '***' 0.001 '**' 0.01 '*' 0.05 '.' 0.1 ' ' 1
```

```
## 
## Correlation matrix not shown by default, as p = 15 > 12.
## Use print(x, correlation=TRUE)  or
##     vcov(x)        if you need it
```

```
## optimizer (nloptwrap) convergence code: 0 (OK)
## boundary (singular) fit: see ?isSingular
```

```
plot(plotVariance(modelC.CU) + scale_fill_manual(values = cbbPalette))
```

Select the best mixed model

```
lc1 <- lmerControl(optimizer = "Nelder_Mead")

modelC_Unit_Flush <- lmer(log(value + 1) ~ factor(Dose)*Day + (1 | Incubator) + (1 | Incubator:ControlUnit) + (1 | Flusher:Day), data = testC, control = lc1)
```

```
## boundary (singular) fit: see ?isSingular
```

```
modelC_Unit <- lmer(log(value + 1) ~ factor(Dose)*Day + (1 | Incubator) + (1 | Incubator:ControlUnit), data = testC, control = lc1)
```

```
## boundary (singular) fit: see ?isSingular
```

```
modelC_Flush <- lmer(log(value + 1) ~ factor(Dose)*Day + (1 | Incubator) + (1 | Flusher:Day), data = testC, control = lc1)
modelC_Full <- lmer(log(value + 1) ~ factor(Dose)*Day + (1 | Incubator) + (1 | Incubator:ControlUnit) + (1 | Incubator:TubeNo) + (1 | Flusher:Day), data = testC, control = lc1)
```

```
## boundary (singular) fit: see ?isSingular
```

```
summary(modelC_Unit_Flush)
```

```
## Linear mixed model fit by REML. t-tests use Satterthwaite's method [
## lmerModLmerTest]
## Formula: log(value + 1) ~ factor(Dose) * Day + (1 | Incubator) + (1 |  
##     Incubator:ControlUnit) + (1 | Flusher:Day)
##    Data: testC
## Control: lc1
## 
## REML criterion at convergence: 234.7
## 
## Scaled residuals: 
##     Min      1Q  Median      3Q     Max 
## -3.2312 -0.4203  0.0794  0.5602  1.8818 
## 
## Random effects:
##  Groups                Name        Variance Std.Dev.
##  Flusher:Day           (Intercept) 0.3599   0.5999  
##  Incubator:ControlUnit (Intercept) 0.3950   0.6285  
##  Incubator             (Intercept) 0.0000   0.0000  
##  Residual                          0.7404   0.8604  
## Number of obs: 90, groups:  
## Flusher:Day, 10; Incubator:ControlUnit, 6; Incubator, 2
## 
## Fixed effects:
##                       Estimate Std. Error       df t value Pr(>|t|)    
## (Intercept)            9.09222    0.61250 11.51957  14.845 7.27e-09 ***
## factor(Dose)1         -0.46826    0.50274 66.67014  -0.931  0.35500    
## factor(Dose)10         0.01421    0.50387 66.84683   0.028  0.97759    
## Dayd7                  0.94085    0.77891  8.49588   1.208  0.25963    
## Dayd14                 0.53168    0.77891  8.49588   0.683  0.51304    
## Dayd21                 0.33446    0.77891  8.49588   0.429  0.67832    
## Dayd28                -0.95437    0.77891  8.49588  -1.225  0.25335    
## factor(Dose)1:Dayd7   -1.09887    0.70255 66.19701  -1.564  0.12256    
## factor(Dose)10:Dayd7  -5.15160    0.70255 66.19701  -7.333 4.07e-10 ***
## factor(Dose)1:Dayd14  -2.05805    0.70255 66.19701  -2.929  0.00465 ** 
## factor(Dose)10:Dayd14 -6.13289    0.70255 66.19701  -8.729 1.29e-12 ***
## factor(Dose)1:Dayd21  -1.19377    0.70255 66.19701  -1.699  0.09398 .  
## factor(Dose)10:Dayd21 -5.74333    0.70255 66.19701  -8.175 1.26e-11 ***
## factor(Dose)1:Dayd28  -0.97727    0.70255 66.19701  -1.391  0.16887    
## factor(Dose)10:Dayd28 -3.79125    0.70255 66.19701  -5.396 9.84e-07 ***
## ---
## Signif. codes:  0 '***' 0.001 '**' 0.01 '*' 0.05 '.' 0.1 ' ' 1
```

```
## 
## Correlation matrix not shown by default, as p = 15 > 12.
## Use print(x, correlation=TRUE)  or
##     vcov(x)        if you need it
```

```
## optimizer (Nelder_Mead) convergence code: 0 (OK)
## boundary (singular) fit: see ?isSingular
```

```
summary(modelC_Unit)
```

```
## Linear mixed model fit by REML. t-tests use Satterthwaite's method [
## lmerModLmerTest]
## Formula: log(value + 1) ~ factor(Dose) * Day + (1 | Incubator) + (1 |  
##     Incubator:ControlUnit)
##    Data: testC
## Control: lc1
## 
## REML criterion at convergence: 244.4
## 
## Scaled residuals: 
##     Min      1Q  Median      3Q     Max 
## -3.3588 -0.4000  0.1210  0.6124  1.6431 
## 
## Random effects:
##  Groups                Name        Variance Std.Dev.
##  Incubator:ControlUnit (Intercept) 0.3733   0.6110  
##  Incubator             (Intercept) 0.0000   0.0000  
##  Residual                          0.9423   0.9707  
## Number of obs: 90, groups:  Incubator:ControlUnit, 6; Incubator, 2
## 
## Fixed effects:
##                       Estimate Std. Error      df t value Pr(>|t|)    
## (Intercept)             9.0834     0.4759 36.5318  19.087  < 2e-16 ***
## factor(Dose)1          -0.4594     0.5668 71.0788  -0.811   0.4204    
## factor(Dose)10          0.0148     0.5680 71.2944   0.026   0.9793    
## Dayd7                   0.9408     0.5604 70.4674   1.679   0.0976 .  
## Dayd14                  0.5317     0.5604 70.4674   0.949   0.3460    
## Dayd21                  0.3345     0.5604 70.4674   0.597   0.5526    
## Dayd28                 -0.9544     0.5604 70.4674  -1.703   0.0930 .  
## factor(Dose)1:Dayd7    -1.0989     0.7926 70.4674  -1.386   0.1700    
## factor(Dose)10:Dayd7   -5.1516     0.7926 70.4674  -6.500 9.83e-09 ***
## factor(Dose)1:Dayd14   -2.0581     0.7926 70.4674  -2.597   0.0114 *  
## factor(Dose)10:Dayd14  -6.1329     0.7926 70.4674  -7.738 5.34e-11 ***
## factor(Dose)1:Dayd21   -1.1938     0.7926 70.4674  -1.506   0.1365    
## factor(Dose)10:Dayd21  -5.7433     0.7926 70.4674  -7.246 4.29e-10 ***
## factor(Dose)1:Dayd28   -0.9773     0.7926 70.4674  -1.233   0.2217    
## factor(Dose)10:Dayd28  -3.7912     0.7926 70.4674  -4.783 9.16e-06 ***
## ---
## Signif. codes:  0 '***' 0.001 '**' 0.01 '*' 0.05 '.' 0.1 ' ' 1
```

```
## 
## Correlation matrix not shown by default, as p = 15 > 12.
## Use print(x, correlation=TRUE)  or
##     vcov(x)        if you need it
```

```
## optimizer (Nelder_Mead) convergence code: 0 (OK)
## boundary (singular) fit: see ?isSingular
```

```
summary(modelC_Flush)
```

```
## Linear mixed model fit by REML. t-tests use Satterthwaite's method [
## lmerModLmerTest]
## Formula: log(value + 1) ~ factor(Dose) * Day + (1 | Incubator) + (1 |  
##     Flusher:Day)
##    Data: testC
## Control: lc1
## 
## REML criterion at convergence: 256.6
## 
## Scaled residuals: 
##     Min      1Q  Median      3Q     Max 
## -3.2189 -0.4716  0.1314  0.5677  1.8200 
## 
## Random effects:
##  Groups      Name        Variance Std.Dev.
##  Flusher:Day (Intercept) 0.35288  0.5940  
##  Incubator   (Intercept) 0.08171  0.2859  
##  Residual                1.13678  1.0662  
## Number of obs: 90, groups:  Flusher:Day, 10; Incubator, 2
## 
## Fixed effects:
##                        Estimate Std. Error        df t value Pr(>|t|)    
## (Intercept)            8.849135   0.637776  9.567222  13.875 1.18e-07 ***
## factor(Dose)1         -0.225172   0.615569 69.999985  -0.366   0.7156    
## factor(Dose)10        -0.006457   0.615569 69.999985  -0.010   0.9917    
## Dayd7                  0.940851   0.855455  9.183326   1.100   0.2994    
## Dayd14                 0.531685   0.855455  9.183326   0.622   0.5494    
## Dayd21                 0.334462   0.855455  9.183326   0.391   0.7047    
## Dayd28                -0.954374   0.855455  9.183326  -1.116   0.2929    
## factor(Dose)1:Dayd7   -1.098873   0.870546 69.999985  -1.262   0.2110    
## factor(Dose)10:Dayd7  -5.151604   0.870546 69.999985  -5.918 1.09e-07 ***
## factor(Dose)1:Dayd14  -2.058053   0.870546 69.999985  -2.364   0.0209 *  
## factor(Dose)10:Dayd14 -6.132887   0.870546 69.999985  -7.045 1.03e-09 ***
## factor(Dose)1:Dayd21  -1.193769   0.870546 69.999985  -1.371   0.1747    
## factor(Dose)10:Dayd21 -5.743329   0.870546 69.999985  -6.597 6.71e-09 ***
## factor(Dose)1:Dayd28  -0.977267   0.870546 69.999985  -1.123   0.2654    
## factor(Dose)10:Dayd28 -3.791249   0.870546 69.999985  -4.355 4.46e-05 ***
## ---
## Signif. codes:  0 '***' 0.001 '**' 0.01 '*' 0.05 '.' 0.1 ' ' 1
```

```
## 
## Correlation matrix not shown by default, as p = 15 > 12.
## Use print(x, correlation=TRUE)  or
##     vcov(x)        if you need it
```

```
summary(modelC_Full)
```

```
## Linear mixed model fit by REML. t-tests use Satterthwaite's method [
## lmerModLmerTest]
## Formula: log(value + 1) ~ factor(Dose) * Day + (1 | Incubator) + (1 |  
##     Incubator:ControlUnit) + (1 | Incubator:TubeNo) + (1 | Flusher:Day)
##    Data: testC
## Control: lc1
## 
## REML criterion at convergence: 213.2
## 
## Scaled residuals: 
##      Min       1Q   Median       3Q      Max 
## -1.93969 -0.41987  0.04501  0.55822  1.86235 
## 
## Random effects:
##  Groups                Name        Variance Std.Dev.
##  Incubator:TubeNo      (Intercept) 0.4273   0.6537  
##  Flusher:Day           (Intercept) 0.4042   0.6358  
##  Incubator:ControlUnit (Intercept) 0.3216   0.5671  
##  Incubator             (Intercept) 0.0000   0.0000  
##  Residual                          0.3994   0.6320  
## Number of obs: 90, groups:  
## Incubator:TubeNo, 18; Flusher:Day, 10; Incubator:ControlUnit, 6; Incubator, 2
## 
## Fixed effects:
##                       Estimate Std. Error       df t value Pr(>|t|)    
## (Intercept)            9.02897    0.63841 12.30611  14.143 5.61e-09 ***
## factor(Dose)1         -0.40500    0.53819 27.51225  -0.753 0.458125    
## factor(Dose)10         0.01254    0.53986 27.83224   0.023 0.981637    
## Dayd7                  0.94085    0.73305  6.40411   1.283 0.243813    
## Dayd14                 0.53168    0.73305  6.40411   0.725 0.493909    
## Dayd21                 0.33446    0.73305  6.40411   0.456 0.663270    
## Dayd28                -0.95437    0.73305  6.40411  -1.302 0.237812    
## factor(Dose)1:Dayd7   -1.09887    0.51600 55.94539  -2.130 0.037618 *  
## factor(Dose)10:Dayd7  -5.15160    0.51600 55.94539  -9.984 4.90e-14 ***
## factor(Dose)1:Dayd14  -2.05805    0.51600 55.94539  -3.988 0.000195 ***
## factor(Dose)10:Dayd14 -6.13289    0.51600 55.94539 -11.885  < 2e-16 ***
## factor(Dose)1:Dayd21  -1.19377    0.51600 55.94539  -2.314 0.024394 *  
## factor(Dose)10:Dayd21 -5.74333    0.51600 55.94539 -11.130 8.29e-16 ***
## factor(Dose)1:Dayd28  -0.97727    0.51600 55.94539  -1.894 0.063409 .  
## factor(Dose)10:Dayd28 -3.79125    0.51600 55.94539  -7.347 9.27e-10 ***
## ---
## Signif. codes:  0 '***' 0.001 '**' 0.01 '*' 0.05 '.' 0.1 ' ' 1
```

```
## 
## Correlation matrix not shown by default, as p = 15 > 12.
## Use print(x, correlation=TRUE)  or
##     vcov(x)        if you need it
```

```
## optimizer (Nelder_Mead) convergence code: 0 (OK)
## boundary (singular) fit: see ?isSingular
```

```
BIC(modelC_Unit_Flush)
```

```
## [1] 320.2317
```

```
BIC(modelC_Unit)
```

```
## [1] 325.4289
```

```
BIC(modelC_Flush)
```

```
## [1] 337.6119
```

```
BIC(modelC_Full)
```

```
## [1] 303.2261
```

```
anova(modelC_Unit_Flush, modelC_Unit, modelC_Flush, modelC_Full)
```

```
## refitting model(s) with ML (instead of REML)
```

```
## Data: testC
## Models:
## modelC_Unit: log(value + 1) ~ factor(Dose) * Day + (1 | Incubator) + (1 | Incubator:ControlUnit)
## modelC_Flush: log(value + 1) ~ factor(Dose) * Day + (1 | Incubator) + (1 | Flusher:Day)
## modelC_Unit_Flush: log(value + 1) ~ factor(Dose) * Day + (1 | Incubator) + (1 | Incubator:ControlUnit) + (1 | Flusher:Day)
## modelC_Full: log(value + 1) ~ factor(Dose) * Day + (1 | Incubator) + (1 | Incubator:ControlUnit) + (1 | Incubator:TubeNo) + (1 | Flusher:Day)
##                   npar    AIC    BIC  logLik deviance  Chisq Df Pr(>Chisq)    
## modelC_Unit         18 280.97 325.96 -122.48   244.97                         
## modelC_Flush        18 299.90 344.90 -131.95   263.90  0.000  0               
## modelC_Unit_Flush   19 276.04 323.53 -119.02   238.04 25.862  1  3.667e-07 ***
## modelC_Full         20 254.16 304.16 -107.08   214.16 23.878  1  1.027e-06 ***
## ---
## Signif. codes:  0 '***' 0.001 '**' 0.01 '*' 0.05 '.' 0.1 ' ' 1
```

Decomposition of variance across endpoints

```
myEndpoints <- c("EarlyErythroid", "EarlyMyeloid", "LateErythroid", "ltHSC", "Platelets")

plotVariance <- function(x)
{
  mySDs <- as.data.frame(VarCorr(x))
  mySDs$pct <- mySDs$vcov/sum(mySDs$vcov)
  p <- ggplot(mySDs, aes(x="", y=pct, fill=grp)) + geom_bar(width=1, stat="identity") + coord_polar("y", start=0)
}

for(k in myEndpoints)
{  
  print(k)
  
  testC <- data.table(full.gathered)[Parameter == k & StudyFraction == "BM-2 Floating",]
  
  summary(lm(log(value + 1) ~ factor(Dose)*Day, data = testC))
  
  ##with flusher
  modelC.flush <- lmer(log(value + 1) ~ factor(Dose)*Day + (1 | Flusher:Day) + (1 | Incubator) + (1 | Incubator:TubeNo), data = testC)
  #summary(modelC.flush)
  plot(plotVariance(modelC.flush) + scale_fill_manual(values = cbbPalette) + ggtitle(k))
  
  ##with control unit
  modelC.CU <- lmer(log(value + 1) ~ factor(Dose)*Day + (1 | Incubator) + (1 | Incubator:ControlUnit), data = testC)
  #summary(modelC.CU)
  plot(plotVariance(modelC.CU) + scale_fill_manual(values = cbbPalette[c(2,5,4)]) + ggtitle(k))
  
  
  ##with both
  modelC.both <- lmer(log(value + 1) ~ factor(Dose)*Day + (1 | Incubator) + (1 | Incubator:ControlUnit) + (1 | Flusher:Day), data = testC, control = lc1)
  #summary(modelC.both)
  plot(plotVariance(modelC.both) + scale_fill_manual(values = cbbPalette[c(1,2,5,4)]) + ggtitle(k))
  
  ##with both + extra term
  modelC.full <- lmer(log(value + 1) ~ factor(Dose)*Day + (1 | Incubator) + (1 | Incubator:ControlUnit) + (1 | Incubator:TubeNo) + (1 | Flusher:Day), data = testC, control = lc1)
  #summary(modelC.full)
  plot(plotVariance(modelC.full) + scale_fill_manual(values = cbbPalette[c(1,2,5,3,4)]) + ggtitle(k))
}
```

```
## [1] "EarlyErythroid"
```

```
## boundary (singular) fit: see ?isSingular
## boundary (singular) fit: see ?isSingular
```

```
## boundary (singular) fit: see ?isSingular
```

```
## boundary (singular) fit: see ?isSingular
```

```
## [1] "EarlyMyeloid"
```

```
## boundary (singular) fit: see ?isSingular
```

```
## boundary (singular) fit: see ?isSingular
```

```
## boundary (singular) fit: see ?isSingular
```

```
## boundary (singular) fit: see ?isSingular
```

```
## Warning: Model failed to converge with 1 negative eigenvalue: -1.3e+00
```

```
## [1] "LateErythroid"
```

```
## boundary (singular) fit: see ?isSingular
```

```
## boundary (singular) fit: see ?isSingular
```

```
## boundary (singular) fit: see ?isSingular
```

```
## [1] "ltHSC"
```

```
## boundary (singular) fit: see ?isSingular
```

```
## boundary (singular) fit: see ?isSingular
```

```
## boundary (singular) fit: see ?isSingular
```

```
## boundary (singular) fit: see ?isSingular
```

```
## [1] "Platelets"
```

```
## boundary (singular) fit: see ?isSingular
```

```
## boundary (singular) fit: see ?isSingular
```

```
## boundary (singular) fit: see ?isSingular
```

From all of this, we claim that modelC.full is the best as it has the lowest BIC, and its variance decomposition is consistent with the other models. Output results from BM-2 to file:

```
output_lm <- NULL
output_flush <- NULL
output_CU <- NULL
output_both <- NULL
output_full <- NULL

chew <- function(x, myK, ...)
{
  ##collect information
  temp <- as.data.frame(tidy(x))

  temp$param <- myK

  temp$estimate_orig_scale <- exp(temp$estimate)
  if("effect" %in% colnames(temp))
  {
    temp[temp$effect == "ran_pars", "estimate_orig_scale"] <- NA
  }
  
  temp$stars <- sigStars(temp$p.value)
  temp
}
  
for(k in myEndpoints)
{  
  print(k)
  
  testC <- data.table(full.gathered)[Parameter == k & StudyFraction == "BM-2 Floating",]
  
  modelC_lm <- lm(log(value + 1) ~ factor(Dose)*Day, data = testC)
  ##with flusher
  modelC_flush <- lmer(log(value + 1) ~ factor(Dose)*Day + (1 | Flusher:Day) + (1 | Incubator) + (1 | Incubator:TubeNo), data = testC, control = lc1)
  ##with control unit
  modelC_CU <- lmer(log(value + 1) ~ factor(Dose)*Day + (1 | Incubator) + (1 | Incubator:ControlUnit), data = testC, control = lc1)
  ##with both
  modelC_both <- lmer(log(value + 1) ~ factor(Dose)*Day + (1 | Incubator) + (1 | Incubator:ControlUnit) + (1 | Flusher:Day), data = testC, control = lc1)
  ##with both + extra term
  modelC_full <- lmer(log(value + 1) ~ factor(Dose)*Day + (1 | Incubator) + (1 | Incubator:ControlUnit) + (1 | Incubator:TubeNo) + (1 | Flusher:Day), data = testC, control = lc1)
  
  temp <- chew(modelC_lm, k)
  temp$effect <- "fixed"
  temp$group <- NA
  output_lm <- rbind(output_lm, temp)
  
  temp <- chew(modelC_flush, k)
  output_flush <- rbind(output_flush, temp)
  
  temp <- chew(modelC_CU, k)
  output_CU <- rbind(output_CU, temp)
  
  temp <- chew(modelC_both, k)
  output_both <- rbind(output_both, temp)
  
  temp <- chew(modelC_full, k)
  output_full <- rbind(output_full, temp)
}
```

```
## [1] "EarlyErythroid"
```

```
## boundary (singular) fit: see ?isSingular
## boundary (singular) fit: see ?isSingular
## boundary (singular) fit: see ?isSingular
## boundary (singular) fit: see ?isSingular
```

```
## [1] "EarlyMyeloid"
```

```
## boundary (singular) fit: see ?isSingular
## boundary (singular) fit: see ?isSingular
## boundary (singular) fit: see ?isSingular
## boundary (singular) fit: see ?isSingular
```

```
## Warning: Model failed to converge with 1 negative eigenvalue: -1.3e+00
```

```
## [1] "LateErythroid"
```

```
## boundary (singular) fit: see ?isSingular
## boundary (singular) fit: see ?isSingular
## boundary (singular) fit: see ?isSingular
## boundary (singular) fit: see ?isSingular
```

```
## [1] "ltHSC"
```

```
## boundary (singular) fit: see ?isSingular
## boundary (singular) fit: see ?isSingular
## boundary (singular) fit: see ?isSingular
## boundary (singular) fit: see ?isSingular
```

```
## [1] "Platelets"
```

```
## boundary (singular) fit: see ?isSingular
## boundary (singular) fit: see ?isSingular
## boundary (singular) fit: see ?isSingular
```

```
write.csv(as.data.frame(output_lm), file = "output_BM-2_lm.csv")
write.csv(as.data.frame(output_flush), file = "output_BM-2_flush.csv")
write.csv(as.data.frame(output_CU), file = "output_BM-2_CU.csv")
write.csv(as.data.frame(output_both), file = "output_BM-2_both.csv")
write.csv(as.data.frame(output_full), file = "output_BM-2_full.csv")
```

```
pander::pander(sessionInfo())
```

**R version 4.1.0 (2021-05-18)**

**Platform:** x86\_64-w64-mingw32/x64 (64-bit)

**locale:** *LC\_COLLATE=English\_United Kingdom.1252*, *LC\_CTYPE=English\_United Kingdom.1252*, *LC\_MONETARY=English\_United Kingdom.1252*, *LC\_NUMERIC=C* and *LC\_TIME=English\_United Kingdom.1252*

**attached base packages:** *grid*, *stats*, *graphics*, *grDevices*, *utils*, *datasets*, *methods* and *base*

**other attached packages:** *here(v.1.0.1)*, *naturalsort(v.0.1.3)*, *broom.mixed(v.0.2.7)*, *MASS(v.7.3-54)*, *lmerTest(v.3.1-3)*, *lme4(v.1.1-27.1)*, *Matrix(v.1.3-3)*, *tidyr(v.1.1.3)*, *dplyr(v.1.0.7)*, *data.table(v.1.14.0)*, *magrittr(v.2.0.1)*, *pca3d(v.0.10.2)*, *ggbiplot(v.0.55)*, *scales(v.1.1.1)*, *plyr(v.1.8.6)* and *ggplot2(v.3.3.5)*

**loaded via a namespace (and not attached):** *rgl(v.0.107.14)*, *Rcpp(v.1.0.7)*, *lattice(v.0.20-44)*, *assertthat(v.0.2.1)*, *rprojroot(v.2.0.2)*, *digest(v.0.6.27)*, *utf8(v.1.2.2)*, *R6(v.2.5.1)*, *backports(v.1.2.1)*, *ellipse(v.0.4.2)*, *evaluate(v.0.14)*, *highr(v.0.9)*, *pillar(v.1.6.2)*, *rlang(v.0.4.11)*, *minqa(v.1.2.4)*, *jquerylib(v.0.1.4)*, *nloptr(v.1.2.2.2)*, *rmarkdown(v.2.11)*, *labeling(v.0.4.2)*, *splines(v.4.1.0)*, *stringr(v.1.4.0)*, *pander(v.0.6.4)*, *htmlwidgets(v.1.5.3)*, *munsell(v.0.5.0)*, *broom(v.0.7.9)*, *compiler(v.4.1.0)*, *numDeriv(v.2016.8-1.1)*, *xfun(v.0.29)*, *pkgconfig(v.2.0.3)*, *htmltools(v.0.5.2)*, *tidyselect(v.1.1.1)*, *tibble(v.3.1.3)*, *fansi(v.0.5.0)*, *crayon(v.1.4.1)*, *withr(v.2.4.2)*, *nlme(v.3.1-152)*, *jsonlite(v.1.7.2)*, *gtable(v.0.3.0)*, *lifecycle(v.1.0.0)*, *DBI(v.1.1.2)*, *stringi(v.1.7.3)*, *farver(v.2.1.0)*, *bslib(v.0.3.1)*, *ellipsis(v.0.3.2)*, *generics(v.0.1.2)*, *vctrs(v.0.3.8)*, *boot(v.1.3-28)*, *tools(v.4.1.0)*, *glue(v.1.4.2)*, *purrr(v.0.3.4)*, *fastmap(v.1.1.0)*, *yaml(v.2.2.1)*, *colorspace(v.2.0-2)*, *knitr(v.1.33)* and *sass(v.0.4.0)*
