# Supplementary material for: Optimal experimental design for efficient toxicity testing in microphysiological systems: A bone marrow application
Source: Front Pharmacol. 2023 Mar 31;14:1142581. doi: 10.3389/fphar.2023.1142581 (PMC10103791; doi:10.3389/fphar.2023.1142581)
Supplement: Supplementary file 2 [file DataSheet1.zip › Data package/Reports/mixedModel_PAPER_BM3.html]

Bone Marrow MPS - compare 2018-04/05


Code 

- Show All Code
- Hide All Code

# Bone Marrow MPS - compare 2018-04/05

#### Statistician: Jonathan Cairns

#### 5 April 2019

Read in data:

```
## new gating not in the first study!!
source("createFull.R")
```

```
## Warning: package 'naturalsort' was built under R version 4.2.2
```

```
## create full_gathered
interestingCols <- c("EarlyErythroid", "LateErythroid", "EarlyMyeloid", "ltHSC", "Platelets", "LineageDiffd", "LateGranulocytes")
metaDataCols <- c(colnames(full)[1:10], "Study", "Group", "Day", "StudyFraction")

## specify the columns of interest


mySel <- c(colnames(full) %>% grep(pattern = "CD"), which(colnames(full) %in% c(interestingCols, paste0(interestingCols, "_pct"))))

full_gathered <- full %>% gather_(
  key_col      = "Parameter",
  value_col    = "value",
  gather_cols  = colnames(full)[mySel]
)
```

```
## Warning: `gather_()` was deprecated in tidyr 1.2.0.
## ℹ Please use `gather()` instead.
```

##Mixed model

Final models

```
plotVariance <- function(x) {
  mySDs <- as.data.frame(VarCorr(x))
  mySDs$pct <- mySDs$vcov / sum(mySDs$vcov)
  p <- ggplot(mySDs, aes(x = "", y = pct, fill = grp)) +
    geom_bar(width = 1, stat = "identity") +
    coord_polar("y", start = 0)
}


testC <- data.table(full_gathered)[Parameter == "LateErythroid" & StudyFraction == "BM-3 Floating", ]
```

Select the best mixed model

```
lc1 <- lmerControl(optimizer = "Nelder_Mead")

modelC_Unit_Flush <- lmer(log(value + 1) ~ factor(Dose) * Day + (1 | Incubator) + (1 | Incubator:ControlUnit) + (1 | Flusher:Day), data = testC, control = lc1)
modelC_Unit <- lmer(log(value + 1) ~ factor(Dose) * Day + (1 | Incubator) + (1 | Incubator:ControlUnit), data = testC, control = lc1)
```

```
## boundary (singular) fit: see help('isSingular')
```

```
modelC_Flush <- lmer(log(value + 1) ~ factor(Dose) * Day + (1 | Incubator) + (1 | Flusher:Day), data = testC, control = lc1)
modelC_Full <- lmer(log(value + 1) ~ factor(Dose) * Day + (1 | Incubator) + (1 | Incubator:ControlUnit) + (1 | Incubator:TubeNo) + (1 | Flusher:Day), data = testC, control = lc1)


summary(modelC_Unit_Flush)
```

```
## Linear mixed model fit by REML. t-tests use Satterthwaite's method [
## lmerModLmerTest]
## Formula: log(value + 1) ~ factor(Dose) * Day + (1 | Incubator) + (1 |  
##     Incubator:ControlUnit) + (1 | Flusher:Day)
##    Data: testC
## Control: lc1
## 
## REML criterion at convergence: 528.8
## 
## Scaled residuals: 
##     Min      1Q  Median      3Q     Max 
## -4.0913 -0.4445  0.0435  0.4813  2.8720 
## 
## Random effects:
##  Groups                Name        Variance Std.Dev.
##  Flusher:Day           (Intercept) 0.179970 0.42423 
##  Incubator:ControlUnit (Intercept) 0.322289 0.56770 
##  Incubator             (Intercept) 0.008363 0.09145 
##  Residual                          0.758071 0.87067 
## Number of obs: 208, groups:  
## Flusher:Day, 14; Incubator:ControlUnit, 6; Incubator, 2
## 
## Fixed effects:
##                        Estimate Std. Error        df t value Pr(>|t|)    
## (Intercept)             7.47707    0.52607  12.89849  14.213 2.96e-09 ***
## factor(Dose)1          -0.67133    0.50518 162.51516  -1.329 0.185749    
## factor(Dose)10          0.12122    0.50746 162.50687   0.239 0.811501    
## factor(Dose)20         -0.05095    0.50518 162.51516  -0.101 0.919798    
## factor(Dose)50         -0.21481    0.50610 162.37441  -0.424 0.671807    
## Dayd1                  -0.16074    0.66115  22.78471  -0.243 0.810087    
## Dayd4                   2.63749    0.66127  22.78303   3.989 0.000587 ***
## Dayd7                   2.45170    0.66115  22.78471   3.708 0.001172 ** 
## Dayd8                   1.60434    0.66115  22.78471   2.427 0.023570 *  
## Dayd11                  1.97750    0.66115  22.78471   2.991 0.006570 ** 
## Dayd14                  1.60544    0.66115  22.78471   2.428 0.023485 *  
## factor(Dose)1:Dayd1     0.33762    0.71403 162.18465   0.473 0.636969    
## factor(Dose)10:Dayd1   -0.08248    0.71403 162.18465  -0.116 0.908176    
## factor(Dose)20:Dayd1    0.29850    0.71403 162.18465   0.418 0.676469    
## factor(Dose)50:Dayd1   -0.11912    0.71403 162.18465  -0.167 0.867715    
## factor(Dose)1:Dayd4     0.24878    0.71414 162.23999   0.348 0.728017    
## factor(Dose)10:Dayd4   -1.16990    0.73045 161.83663  -1.602 0.111192    
## factor(Dose)20:Dayd4   -3.17136    0.71414 162.23999  -4.441 1.65e-05 ***
## factor(Dose)50:Dayd4   -5.47702    0.73060 161.84365  -7.497 4.06e-12 ***
## factor(Dose)1:Dayd7     0.18993    0.71403 162.18465   0.266 0.790583    
## factor(Dose)10:Dayd7   -1.66809    0.71403 162.18465  -2.336 0.020707 *  
## factor(Dose)20:Dayd7   -3.82346    0.71403 162.18465  -5.355 2.89e-07 ***
## factor(Dose)50:Dayd7   -6.34061    0.71403 162.18465  -8.880 1.20e-15 ***
## factor(Dose)1:Dayd8     0.32568    0.71403 162.18465   0.456 0.648923    
## factor(Dose)10:Dayd8   -1.66885    0.71403 162.18465  -2.337 0.020650 *  
## factor(Dose)20:Dayd8   -4.30795    0.71403 162.18465  -6.033 1.05e-08 ***
## factor(Dose)50:Dayd8   -7.06266    0.71403 162.18465  -9.891  < 2e-16 ***
## factor(Dose)1:Dayd11    0.28949    0.71403 162.18465   0.405 0.685692    
## factor(Dose)10:Dayd11  -1.52177    0.71403 162.18465  -2.131 0.034577 *  
## factor(Dose)20:Dayd11  -3.98471    0.71403 162.18465  -5.581 9.86e-08 ***
## factor(Dose)50:Dayd11  -8.09963    0.71403 162.18465 -11.343  < 2e-16 ***
## factor(Dose)1:Dayd14   -0.03307    0.71403 162.18465  -0.046 0.963122    
## factor(Dose)10:Dayd14  -1.40502    0.71403 162.18465  -1.968 0.050805 .  
## factor(Dose)20:Dayd14  -3.49480    0.71403 162.18465  -4.894 2.36e-06 ***
## factor(Dose)50:Dayd14  -8.39424    0.71403 162.18465 -11.756  < 2e-16 ***
## ---
## Signif. codes:  0 '***' 0.001 '**' 0.01 '*' 0.05 '.' 0.1 ' ' 1
```

```
## 
## Correlation matrix not shown by default, as p = 35 > 12.
## Use print(x, correlation=TRUE)  or
##     vcov(x)        if you need it
```

```
summary(modelC_Unit)
```

```
## Linear mixed model fit by REML. t-tests use Satterthwaite's method [
## lmerModLmerTest]
## Formula: log(value + 1) ~ factor(Dose) * Day + (1 | Incubator) + (1 |  
##     Incubator:ControlUnit)
##    Data: testC
## Control: lc1
## 
## REML criterion at convergence: 539.9
## 
## Scaled residuals: 
##     Min      1Q  Median      3Q     Max 
## -4.0473 -0.5126 -0.0122  0.5940  2.6810 
## 
## Random effects:
##  Groups                Name        Variance  Std.Dev. 
##  Incubator:ControlUnit (Intercept) 3.333e-01 5.773e-01
##  Incubator             (Intercept) 6.196e-11 7.871e-06
##  Residual                          8.583e-01 9.264e-01
## Number of obs: 208, groups:  Incubator:ControlUnit, 6; Incubator, 2
## 
## Fixed effects:
##                         Estimate Std. Error         df t value Pr(>|t|)    
## (Intercept)             7.588741   0.445641  46.952686  17.029  < 2e-16 ***
## factor(Dose)1          -0.782998   0.534871 167.953664  -1.464 0.145091    
## factor(Dose)10          0.007449   0.537636 168.253591   0.014 0.988962    
## factor(Dose)20         -0.162614   0.534871 167.953664  -0.304 0.761485    
## factor(Dose)50         -0.332952   0.536169 168.088842  -0.621 0.535451    
## Dayd1                  -0.366316   0.534871 167.953664  -0.685 0.494372    
## Dayd4                   2.366594   0.534871 167.953664   4.425 1.73e-05 ***
## Dayd7                   2.407519   0.534871 167.953664   4.501 1.26e-05 ***
## Dayd8                   1.477308   0.534871 167.953664   2.762 0.006385 ** 
## Dayd11                  1.855167   0.534871 167.953664   3.468 0.000665 ***
## Dayd14                  1.467348   0.534871 167.953664   2.743 0.006742 ** 
## factor(Dose)1:Dayd1     0.543192   0.756422 167.953664   0.718 0.473689    
## factor(Dose)10:Dayd1    0.123091   0.756422 167.953664   0.163 0.870928    
## factor(Dose)20:Dayd1    0.504071   0.756422 167.953664   0.666 0.506077    
## factor(Dose)50:Dayd1    0.086457   0.756422 167.953664   0.114 0.909139    
## factor(Dose)1:Dayd4     0.519680   0.756422 167.953664   0.687 0.493015    
## factor(Dose)10:Dayd4   -0.987350   0.775425 167.972506  -1.273 0.204670    
## factor(Dose)20:Dayd4   -2.900462   0.756422 167.953664  -3.834 0.000178 ***
## factor(Dose)50:Dayd4   -5.295352   0.775594 167.984115  -6.827 1.51e-10 ***
## factor(Dose)1:Dayd7     0.234103   0.756422 167.953664   0.309 0.757334    
## factor(Dose)10:Dayd7   -1.623911   0.756422 167.953664  -2.147 0.033242 *  
## factor(Dose)20:Dayd7   -3.779285   0.756422 167.953664  -4.996 1.46e-06 ***
## factor(Dose)50:Dayd7   -6.296429   0.756422 167.953664  -8.324 2.84e-14 ***
## factor(Dose)1:Dayd8     0.452705   0.756422 167.953664   0.598 0.550324    
## factor(Dose)10:Dayd8   -1.541817   0.756422 167.953664  -2.038 0.043088 *  
## factor(Dose)20:Dayd8   -4.180920   0.756422 167.953664  -5.527 1.22e-07 ***
## factor(Dose)50:Dayd8   -6.935630   0.756422 167.953664  -9.169  < 2e-16 ***
## factor(Dose)1:Dayd11    0.411825   0.756422 167.953664   0.544 0.586862    
## factor(Dose)10:Dayd11  -1.399438   0.756422 167.953664  -1.850 0.066060 .  
## factor(Dose)20:Dayd11  -3.862374   0.756422 167.953664  -5.106 8.84e-07 ***
## factor(Dose)50:Dayd11  -7.977296   0.756422 167.953664 -10.546  < 2e-16 ***
## factor(Dose)1:Dayd14    0.105029   0.756422 167.953664   0.139 0.889735    
## factor(Dose)10:Dayd14  -1.266924   0.756422 167.953664  -1.675 0.095817 .  
## factor(Dose)20:Dayd14  -3.356701   0.756422 167.953664  -4.438 1.64e-05 ***
## factor(Dose)50:Dayd14  -8.256149   0.756422 167.953664 -10.915  < 2e-16 ***
## ---
## Signif. codes:  0 '***' 0.001 '**' 0.01 '*' 0.05 '.' 0.1 ' ' 1
```

```
## 
## Correlation matrix not shown by default, as p = 35 > 12.
## Use print(x, correlation=TRUE)  or
##     vcov(x)        if you need it
```

```
## optimizer (Nelder_Mead) convergence code: 0 (OK)
## boundary (singular) fit: see help('isSingular')
```

```
summary(modelC_Flush)
```

```
## Linear mixed model fit by REML. t-tests use Satterthwaite's method [
## lmerModLmerTest]
## Formula: log(value + 1) ~ factor(Dose) * Day + (1 | Incubator) + (1 |  
##     Flusher:Day)
##    Data: testC
## Control: lc1
## 
## REML criterion at convergence: 563.1
## 
## Scaled residuals: 
##     Min      1Q  Median      3Q     Max 
## -3.7894 -0.5025  0.0322  0.5191  2.7191 
## 
## Random effects:
##  Groups      Name        Variance Std.Dev.
##  Flusher:Day (Intercept) 0.1764   0.4200  
##  Incubator   (Intercept) 0.1149   0.3389  
##  Residual                0.9902   0.9951  
## Number of obs: 208, groups:  Flusher:Day, 14; Incubator, 2
## 
## Fixed effects:
##                        Estimate Std. Error        df t value Pr(>|t|)    
## (Intercept)             7.49054    0.55980  15.85268  13.381 4.72e-10 ***
## factor(Dose)1          -0.68480    0.57688 165.88678  -1.187 0.236896    
## factor(Dose)10         -0.03083    0.57727 165.90586  -0.053 0.957474    
## factor(Dose)20         -0.06442    0.57688 165.88678  -0.112 0.911227    
## factor(Dose)50         -0.25720    0.57688 165.88678  -0.446 0.656286    
## Dayd1                  -0.17495    0.71546  28.23127  -0.245 0.808591    
## Dayd4                   2.62690    0.71558  28.22016   3.671 0.000999 ***
## Dayd7                   2.44864    0.71546  28.23127   3.422 0.001913 ** 
## Dayd8                   1.59556    0.71546  28.23127   2.230 0.033865 *  
## Dayd11                  1.96904    0.71546  28.23127   2.752 0.010231 *  
## Dayd14                  1.59590    0.71546  28.23127   2.231 0.033830 *  
## factor(Dose)1:Dayd1     0.35183    0.81583 165.88580   0.431 0.666847    
## factor(Dose)10:Dayd1   -0.06828    0.81583 165.88580  -0.084 0.933406    
## factor(Dose)20:Dayd1    0.31271    0.81583 165.88580   0.383 0.701991    
## factor(Dose)50:Dayd1   -0.10491    0.81583 165.88580  -0.129 0.897836    
## factor(Dose)1:Dayd4     0.25937    0.81594 165.94964   0.318 0.750973    
## factor(Dose)10:Dayd4   -1.08778    0.83450 165.45023  -1.304 0.194215    
## factor(Dose)20:Dayd4   -3.16077    0.81594 165.94964  -3.874 0.000154 ***
## factor(Dose)50:Dayd4   -5.37297    0.83456 165.45207  -6.438 1.26e-09 ***
## factor(Dose)1:Dayd7     0.19298    0.81583 165.88580   0.237 0.813302    
## factor(Dose)10:Dayd7   -1.66503    0.81583 165.88580  -2.041 0.042845 *  
## factor(Dose)20:Dayd7   -3.82041    0.81583 165.88580  -4.683 5.86e-06 ***
## factor(Dose)50:Dayd7   -6.33755    0.81583 165.88580  -7.768 7.86e-13 ***
## factor(Dose)1:Dayd8     0.33446    0.81583 165.88580   0.410 0.682366    
## factor(Dose)10:Dayd8   -1.66007    0.81583 165.88580  -2.035 0.043461 *  
## factor(Dose)20:Dayd8   -4.29917    0.81583 165.88580  -5.270 4.20e-07 ***
## factor(Dose)50:Dayd8   -7.05388    0.81583 165.88580  -8.646 4.36e-15 ***
## factor(Dose)1:Dayd11    0.29795    0.81583 165.88580   0.365 0.715420    
## factor(Dose)10:Dayd11  -1.51331    0.81583 165.88580  -1.855 0.065380 .  
## factor(Dose)20:Dayd11  -3.97625    0.81583 165.88580  -4.874 2.54e-06 ***
## factor(Dose)50:Dayd11  -8.09117    0.81583 165.88580  -9.918  < 2e-16 ***
## factor(Dose)1:Dayd14   -0.02352    0.81583 165.88580  -0.029 0.977035    
## factor(Dose)10:Dayd14  -1.39547    0.81583 165.88580  -1.710 0.089044 .  
## factor(Dose)20:Dayd14  -3.48525    0.81583 165.88580  -4.272 3.26e-05 ***
## factor(Dose)50:Dayd14  -8.38470    0.81583 165.88580 -10.277  < 2e-16 ***
## ---
## Signif. codes:  0 '***' 0.001 '**' 0.01 '*' 0.05 '.' 0.1 ' ' 1
```

```
## 
## Correlation matrix not shown by default, as p = 35 > 12.
## Use print(x, correlation=TRUE)  or
##     vcov(x)        if you need it
```

```
summary(modelC_Full)
```

```
## Linear mixed model fit by REML. t-tests use Satterthwaite's method [
## lmerModLmerTest]
## Formula: log(value + 1) ~ factor(Dose) * Day + (1 | Incubator) + (1 |  
##     Incubator:ControlUnit) + (1 | Incubator:TubeNo) + (1 | Flusher:Day)
##    Data: testC
## Control: lc1
## 
## REML criterion at convergence: 510.9
## 
## Scaled residuals: 
##     Min      1Q  Median      3Q     Max 
## -3.8515 -0.4036  0.0182  0.5324  2.4026 
## 
## Random effects:
##  Groups                Name        Variance Std.Dev.
##  Incubator:TubeNo      (Intercept) 0.204726 0.45247 
##  Flusher:Day           (Intercept) 0.193365 0.43973 
##  Incubator:ControlUnit (Intercept) 0.284822 0.53369 
##  Incubator             (Intercept) 0.007129 0.08443 
##  Residual                          0.584388 0.76445 
## Number of obs: 208, groups:  
## Incubator:TubeNo, 30; Flusher:Day, 14; Incubator:ControlUnit, 6; Incubator, 2
## 
## Fixed effects:
##                        Estimate Std. Error        df t value Pr(>|t|)    
## (Intercept)             7.46416    0.53079  13.20925  14.062 2.50e-09 ***
## factor(Dose)1          -0.65841    0.51531 105.17527  -1.278 0.204164    
## factor(Dose)10          0.12837    0.52073 102.59588   0.247 0.805775    
## factor(Dose)20         -0.03803    0.51531 105.17527  -0.074 0.941308    
## factor(Dose)50         -0.20972    0.51769 103.75345  -0.405 0.686231    
## Dayd1                  -0.14691    0.62597  17.83265  -0.235 0.817120    
## Dayd4                   2.65358    0.62608  17.83638   4.238 0.000503 ***
## Dayd7                   2.45467    0.62597  17.83265   3.921 0.001016 ** 
## Dayd8                   1.61288    0.62597  17.83265   2.577 0.019104 *  
## Dayd11                  1.98573    0.62597  17.83265   3.172 0.005321 ** 
## Dayd14                  1.61473    0.62597  17.83265   2.580 0.018986 *  
## factor(Dose)1:Dayd1     0.32379    0.62711 142.59792   0.516 0.606432    
## factor(Dose)10:Dayd1   -0.09631    0.62711 142.59792  -0.154 0.878153    
## factor(Dose)20:Dayd1    0.28467    0.62711 142.59792   0.454 0.650566    
## factor(Dose)50:Dayd1   -0.13295    0.62711 142.59792  -0.212 0.832407    
## factor(Dose)1:Dayd4     0.23269    0.62723 142.64647   0.371 0.711200    
## factor(Dose)10:Dayd4   -1.20797    0.64290 142.92352  -1.879 0.062288 .  
## factor(Dose)20:Dayd4   -3.18745    0.62723 142.64647  -5.082 1.15e-06 ***
## factor(Dose)50:Dayd4   -5.50885    0.64294 142.89010  -8.568 1.54e-14 ***
## factor(Dose)1:Dayd7     0.18695    0.62711 142.59792   0.298 0.766043    
## factor(Dose)10:Dayd7   -1.67106    0.62711 142.59792  -2.665 0.008594 ** 
## factor(Dose)20:Dayd7   -3.82643    0.62711 142.59792  -6.102 9.38e-09 ***
## factor(Dose)50:Dayd7   -6.34358    0.62711 142.59792 -10.116  < 2e-16 ***
## factor(Dose)1:Dayd8     0.31713    0.62711 142.59792   0.506 0.613846    
## factor(Dose)10:Dayd8   -1.67739    0.62711 142.59792  -2.675 0.008351 ** 
## factor(Dose)20:Dayd8   -4.31650    0.62711 142.59792  -6.883 1.71e-10 ***
## factor(Dose)50:Dayd8   -7.07121    0.62711 142.59792 -11.276  < 2e-16 ***
## factor(Dose)1:Dayd11    0.28126    0.62711 142.59792   0.449 0.654465    
## factor(Dose)10:Dayd11  -1.53000    0.62711 142.59792  -2.440 0.015924 *  
## factor(Dose)20:Dayd11  -3.99293    0.62711 142.59792  -6.367 2.48e-09 ***
## factor(Dose)50:Dayd11  -8.10786    0.62711 142.59792 -12.929  < 2e-16 ***
## factor(Dose)1:Dayd14   -0.04236    0.62711 142.59792  -0.068 0.946245    
## factor(Dose)10:Dayd14  -1.41431    0.62711 142.59792  -2.255 0.025638 *  
## factor(Dose)20:Dayd14  -3.50409    0.62711 142.59792  -5.588 1.13e-07 ***
## factor(Dose)50:Dayd14  -8.40353    0.62711 142.59792 -13.400  < 2e-16 ***
## ---
## Signif. codes:  0 '***' 0.001 '**' 0.01 '*' 0.05 '.' 0.1 ' ' 1
```

```
## 
## Correlation matrix not shown by default, as p = 35 > 12.
## Use print(x, correlation=TRUE)  or
##     vcov(x)        if you need it
```

```
BIC(modelC_Unit_Flush)
```

```
## [1] 736.9291
```

```
BIC(modelC_Unit)
```

```
## [1] 742.7723
```

```
BIC(modelC_Flush)
```

```
## [1] 765.9052
```

```
BIC(modelC_Full)
```

```
## [1] 724.3903
```

```
anova(modelC_Unit_Flush, modelC_Unit, modelC_Flush, modelC_Full)
```

```
## refitting model(s) with ML (instead of REML)
```

```
## Data: testC
## Models:
## modelC_Unit: log(value + 1) ~ factor(Dose) * Day + (1 | Incubator) + (1 | Incubator:ControlUnit)
## modelC_Flush: log(value + 1) ~ factor(Dose) * Day + (1 | Incubator) + (1 | Flusher:Day)
## modelC_Unit_Flush: log(value + 1) ~ factor(Dose) * Day + (1 | Incubator) + (1 | Incubator:ControlUnit) + (1 | Flusher:Day)
## modelC_Full: log(value + 1) ~ factor(Dose) * Day + (1 | Incubator) + (1 | Incubator:ControlUnit) + (1 | Incubator:TubeNo) + (1 | Flusher:Day)
##                   npar    AIC    BIC  logLik deviance  Chisq Df Pr(>Chisq)    
## modelC_Unit         38 612.09 738.92 -268.05   536.09                         
## modelC_Flush        38 646.55 773.37 -285.27   570.55  0.000  0               
## modelC_Unit_Flush   39 606.70 736.87 -264.35   528.70 41.843  1  9.891e-11 ***
## modelC_Full         40 588.47 721.97 -254.23   508.47 20.235  1  6.849e-06 ***
## ---
## Signif. codes:  0 '***' 0.001 '**' 0.01 '*' 0.05 '.' 0.1 ' ' 1
```

Decomposition of variance across endpoints

```
myEndpoints <- c("EarlyErythroid", "EarlyMyeloid", "LateErythroid", "ltHSC", "Platelets")

plotVariance <- function(x) {
  mySDs <- as.data.frame(VarCorr(x))
  mySDs$pct <- mySDs$vcov / sum(mySDs$vcov)
  p <- ggplot(mySDs, aes(x = "", y = pct, fill = grp)) +
    geom_bar(width = 1, stat = "identity") +
    coord_polar("y", start = 0)
}

for (k in myEndpoints)
{
  print(k)

  testC <- data.table(full_gathered)[Parameter == k & StudyFraction == "BM-3 Floating", ]

  summary(lm(log(value + 1) ~ factor(Dose) * Day, data = testC))

  ## with flusher
  modelC_flush <- lmer(log(value + 1) ~ factor(Dose) * Day + (1 | Flusher:Day) + (1 | Incubator) + (1 | Incubator:TubeNo), data = testC)
  plot(plotVariance(modelC_flush) + scale_fill_manual(values = cbbPalette) + ggtitle(k))

  ## with control unit
  modelC_CU <- lmer(log(value + 1) ~ factor(Dose) * Day + (1 | Incubator) + (1 | Incubator:ControlUnit), data = testC)
  plot(plotVariance(modelC_CU) + scale_fill_manual(values = cbbPalette[c(2, 5, 4)]) + ggtitle(k))


  ## with both
  modelC_both <- lmer(log(value + 1) ~ factor(Dose) * Day + (1 | Incubator) + (1 | Incubator:ControlUnit) + (1 | Flusher:Day), data = testC, control = lc1)
  plot(plotVariance(modelC_both) + scale_fill_manual(values = cbbPalette[c(1, 2, 5, 4)]) + ggtitle(k))

  ## with both + extra term
  modelC_full <- lmer(log(value + 1) ~ factor(Dose) * Day + (1 | Incubator) + (1 | Incubator:ControlUnit) + (1 | Incubator:TubeNo) + (1 | Flusher:Day), data = testC, control = lc1)
  plot(plotVariance(modelC_full) + scale_fill_manual(values = cbbPalette[c(1, 2, 5, 3, 4)]) + ggtitle(k))
}
```

```
## [1] "EarlyErythroid"
```

```
## Warning in checkConv(attr(opt, "derivs"), opt$par, ctrl = control$checkConv, :
## Model failed to converge with max|grad| = 0.00388521 (tol = 0.002, component 1)
```

```
## boundary (singular) fit: see help('isSingular')
```

```
## boundary (singular) fit: see help('isSingular')
```

```
## boundary (singular) fit: see help('isSingular')
```

```
## [1] "EarlyMyeloid"
```

```
## boundary (singular) fit: see help('isSingular')
```

```
## boundary (singular) fit: see help('isSingular')
```

```
## [1] "LateErythroid"
```

```
## [1] "ltHSC"
```

```
## boundary (singular) fit: see help('isSingular')
```

```
## boundary (singular) fit: see help('isSingular')
```

```
## [1] "Platelets"
```

```
## boundary (singular) fit: see help('isSingular')
```

Plots for Figures 6G and 6H:

```
myEndpoints <- c("LateErythroid", "Platelets")

plotVariance <- function(x) {
  mySDs <- as.data.frame(VarCorr(x))
  mySDs$grp[mySDs$grp == "Flusher:Day"] <- "Operator:Day"
  mySDs$grp <- factor(mySDs$grp)
  mySDs$grp <- relevel(mySDs$grp, ref = "Operator:Day")
  
  mySDs$pct <- mySDs$vcov / sum(mySDs$vcov)
  p <- ggplot(mySDs, aes(x = "", y = pct, fill = grp)) +
    geom_bar(width = 1, stat = "identity") +
    coord_polar("y", start = 0)
}


k = "LateErythroid"
  
  testC <- data.table(full_gathered)[Parameter == k & StudyFraction == "BM-3 Floating", ]

  ## with both + extra term
  modelC_full <- lmer(log(value + 1) ~ factor(Dose) * Day + (1 | Incubator) + (1 | Incubator:ControlUnit) + (1 | Incubator:TubeNo) + (1 | Flusher:Day), data = testC, control = lc1)
  plot(
    plotVariance(modelC_full) +
         scale_fill_manual(values = cbbPalette[c(1, 2, 5, 3, 4)]) +
         theme_void() +
         theme(plot.title = element_text(size=30), legend.position="none") +
         ggtitle("Late Erythroid")
    )
```

```
k = "Platelets"
  
  testC <- data.table(full_gathered)[Parameter == k & StudyFraction == "BM-3 Floating", ]

  
  ## with both + extra term
  modelC_full <- lmer(log(value + 1) ~ factor(Dose) * Day + (1 | Incubator) + (1 | Incubator:ControlUnit) + (1 | Incubator:TubeNo) + (1 | Flusher:Day), data = testC, control = lc1)
```

```
## boundary (singular) fit: see help('isSingular')
```

```
  plot(
    plotVariance(modelC_full) +
         scale_fill_manual(values = cbbPalette[c(1, 2, 5, 3, 4)]) +
         theme_void() +
         theme(
           plot.title = element_text(size=30),
           legend.title = element_text(size=30),
           legend.text = element_text(size = 30),
           legend.key.width = unit(1.5,"cm"),
           legend.key.height = unit(1.5,"cm")
         ) +
         ggtitle(k)
    )
```

From all of this, we claim that modelC.full is the best as it has the
lowest BIC, and its variance decomposition is consistent with the other
models. Output results from BM-3 to file:

```
output_lm <- NULL
output_flush <- NULL
output_CU <- NULL
output_both <- NULL
output_full <- NULL

chew <- function(x, myK, ...) {
  ## collect information
  temp <- as.data.frame(tidy(x))

  temp$param <- myK

  temp$estimate_orig_scale <- exp(temp$estimate)
  if ("effect" %in% colnames(temp)) {
    temp[temp$effect == "ran_pars", "estimate_orig_scale"] <- NA
  }

  temp$stars <- sigStars(temp$p.value)
  temp
}

for (k in myEndpoints) {
  print(k)

  testC <- data.table(full_gathered)[Parameter == k & StudyFraction == "BM-3 Floating", ]

  modelC_lm <- lm(log(value + 1) ~ factor(Dose) * Day, data = testC)
  ## with flusher
  modelC_flush <- lmer(log(value + 1) ~ factor(Dose) * Day + (1 | Flusher:Day) + (1 | Incubator) + (1 | Incubator:TubeNo), data = testC, control = lc1)
  ## with control unit
  modelC_CU <- lmer(log(value + 1) ~ factor(Dose) * Day + (1 | Incubator) + (1 | Incubator:ControlUnit), data = testC, control = lc1)
  ## with both
  modelC_both <- lmer(log(value + 1) ~ factor(Dose) * Day + (1 | Incubator) + (1 | Incubator:ControlUnit) + (1 | Flusher:Day), data = testC, control = lc1)
  ## with both + extra term
  modelC_full <- lmer(log(value + 1) ~ factor(Dose) * Day + (1 | Incubator) + (1 | Incubator:ControlUnit) + (1 | Incubator:TubeNo) + (1 | Flusher:Day), data = testC, control = lc1)

  temp <- chew(modelC_lm, k)
  temp$effect <- "fixed"
  temp$group <- NA
  output_lm <- rbind(output_lm, temp)

  temp <- chew(modelC_flush, k)
  output_flush <- rbind(output_flush, temp)

  temp <- chew(modelC_CU, k)
  output_CU <- rbind(output_CU, temp)

  temp <- chew(modelC_both, k)
  output_both <- rbind(output_both, temp)

  temp <- chew(modelC_full, k)
  output_full <- rbind(output_full, temp)
}
```

```
## [1] "LateErythroid"
```

```
## boundary (singular) fit: see help('isSingular')
```

```
## [1] "Platelets"
```

```
## boundary (singular) fit: see help('isSingular')
```

```
write.csv(as.data.frame(output_lm), file = "output_BM-3_lm.csv")
write.csv(as.data.frame(output_flush), file = "output_BM-3_flush.csv")
write.csv(as.data.frame(output_CU), file = "output_BM-3_CU.csv")
write.csv(as.data.frame(output_both), file = "output_BM-3_both.csv")
write.csv(as.data.frame(output_full), file = "output_BM-3_full.csv")
```

```
pander::pander(sessionInfo())
```

**R version 4.2.1 (2022-06-23 ucrt)**

**Platform:** x86\_64-w64-mingw32/x64 (64-bit)

**locale:** *LC\_COLLATE=English\_United
Kingdom.utf8*, *LC\_CTYPE=English\_United Kingdom.utf8*,
*LC\_MONETARY=English\_United Kingdom.utf8*, *LC\_NUMERIC=C*
and *LC\_TIME=English\_United Kingdom.utf8*

**attached base packages:** *grid*,
*stats*, *graphics*, *grDevices*, *utils*,
*datasets*, *methods* and *base*

**other attached packages:**
*naturalsort(v.0.1.3)*, *here(v.1.0.1)*,
*broom.mixed(v.0.2.9.4)*, *broom(v.1.0.1)*,
*MASS(v.7.3-57)*, *lmerTest(v.3.1-3)*,
*lme4(v.1.1-30)*, *Matrix(v.1.5-1)*,
*tidyr(v.1.2.1)*, *dplyr(v.1.0.10)*,
*data.table(v.1.14.2)*, *magrittr(v.2.0.3)*,
*ggbiplot(v.0.55)*, *scales(v.1.2.1)*,
*plyr(v.1.8.7)* and *ggplot2(v.3.4.0)*

**loaded via a namespace (and not attached):**
*Rcpp(v.1.0.9)*, *lattice(v.0.20-45)*,
*listenv(v.0.9.0)*, *rprojroot(v.2.0.3)*,
*assertthat(v.0.2.1)*, *digest(v.0.6.29)*,
*utf8(v.1.2.2)*, *parallelly(v.1.34.0)*,
*R6(v.2.5.1)*, *backports(v.1.4.1)*,
*evaluate(v.0.17)*, *highr(v.0.9)*,
*pillar(v.1.8.1)*, *rlang(v.1.0.6)*,
*rstudioapi(v.0.14)*, *minqa(v.1.2.4)*,
*furrr(v.0.3.1)*, *jquerylib(v.0.1.4)*,
*nloptr(v.2.0.3)*, *rmarkdown(v.2.17)*,
*labeling(v.0.4.2)*, *splines(v.4.2.1)*,
*pander(v.0.6.5)*, *stringr(v.1.4.1)*,
*munsell(v.0.5.0)*, *compiler(v.4.2.1)*,
*numDeriv(v.2016.8-1.1)*, *xfun(v.0.31)*,
*pkgconfig(v.2.0.3)*, *globals(v.0.16.2)*,
*htmltools(v.0.5.3)*, *tidyselect(v.1.2.0)*,
*tibble(v.3.1.8)*, *codetools(v.0.2-18)*,
*fansi(v.1.0.3)*, *future(v.1.31.0)*,
*withr(v.2.5.0)*, *nlme(v.3.1-157)*,
*jsonlite(v.1.8.2)*, *gtable(v.0.3.1)*,
*lifecycle(v.1.0.3)*, *DBI(v.1.1.3)*,
*cli(v.3.4.1)*, *stringi(v.1.7.8)*,
*cachem(v.1.0.6)*, *farver(v.2.1.1)*,
*bslib(v.0.4.0)*, *ellipsis(v.0.3.2)*,
*generics(v.0.1.3)*, *vctrs(v.0.5.1)*,
*boot(v.1.3-28)*, *tools(v.4.2.1)*,
*forcats(v.0.5.2)*, *glue(v.1.6.2)*,
*purrr(v.0.3.5)*, *parallel(v.4.2.1)*,
*fastmap(v.1.1.0)*, *yaml(v.2.3.5)*,
*colorspace(v.2.0-3)*, *knitr(v.1.40)* and
*sass(v.0.4.2)*
