# Supplementary material for: Optimal experimental design for efficient toxicity testing in microphysiological systems: A bone marrow application
Source: Front Pharmacol. 2023 Mar 31;14:1142581. doi: 10.3389/fphar.2023.1142581 (PMC10103791; doi:10.3389/fphar.2023.1142581)
Supplement: Supplementary file 2 [file DataSheet1.zip › Data package/Reports/mixedModel_plot_PAPER_BM-1.html]

Bone Marrow MPS - compare 2018-04/05


Code 

- Show All Code
- Hide All Code

# Bone Marrow MPS - compare 2018-04/05

#### Statistician: Jonathan Cairns

#### 5 April 2019

Read in data:

```
files <- dir(pattern = "output_BM-1")
x <- lapply(files, fread)

files <- gsub("output_BM-1_", "", files, fixed = TRUE)
files <- gsub(".csv", "", files, fixed = TRUE)
names(x) <- files
```

Assemble output

```
output <- NULL

for(i in seq_along(x))
{
  temp <- x[[i]]
  temp <- temp[is.na(temp$group),]
  temp <- temp[grepl(pattern = ":", temp$term),]
  
  extra_info <- as.data.table(do.call(rbind, strsplit(temp$term, ":")))
  colnames(extra_info) <- c("Dose", "Day")
  extra_info$Dose <- gsub("factor(Dose)", "", extra_info$Dose, fixed = TRUE)
  extra_info$Day <- gsub("Day", "", extra_info$Day)
  temp <- cbind(temp, extra_info)
  temp$minus_log10_p <- -log10(temp$p.value)
  temp$model <- files[i]
  
  output <- rbind(output, temp, fill = TRUE)
}

output$Day <- naturalfactor(output$Day)
```

plot heatmaps

Everything (supplementary)

```
  p <- ggplot(aes(x=Day, y=Dose, fill=minus_log10_p), data = output) + 
    geom_tile(colour="black") + 
    #geom_label(aes(label = stars), color = "black", fill = "white") + 
    #geom_text(aes(label = stars), color = ifelse(output$p.value < 0.005, "black", "white")) +
    geom_text(aes(label = stars), color = "black", size = 7) +
    theme_bw() + 
    scale_fill_gradient2(low = "blue",mid = "yellow", high = "red", midpoint = 15) + 
    facet_grid(model ~ param) #+
    
  print(p)
```

Model comparison

```
  p <- ggplot(aes(x=Day, y=Dose, fill=minus_log10_p), data = output[param == "LateErythroid",]) + 
    geom_tile(colour="black") + 
    #geom_label(aes(label = stars), color = "black", fill = "white") + 
    #geom_text(aes(label = stars), color = ifelse(output$p.value < 0.005, "black", "white")) +
    geom_text(aes(label = stars), color = "black", size = 8) +
    theme_bw() + 
    scale_fill_gradient2(low = "blue",mid = "yellow", high = "red", midpoint = 15) + 
    facet_grid(. ~ model) #+
    
  print(p)
```

```
p <- ggplot(aes(x=Day, y=Dose, fill=estimate), data = output) + 
    geom_tile(colour="black") + 
    geom_text(aes(label = stars)) +
    theme_bw() + 
    scale_fill_gradient(low = "dark blue", high = "yellow") + 
    facet_grid(model ~ param) #+
    
  print(p)
```

```
p <- ggplot(aes(x=Day, y=Dose, fill=minus_log10_p), data = output) + 
    geom_tile(colour="black") + 
    geom_text(aes(label = signif(p.value, 3))) +
    theme_bw() + 
    scale_fill_gradient2(low = "white",mid = "yellow", high = "red", midpoint = 15) + 
    facet_grid(model ~ param) #+
    
  print(p)
```

```
pander::pander(sessionInfo())
```

**R version 4.1.0 (2021-05-18)**

**Platform:** x86\_64-w64-mingw32/x64 (64-bit)

**locale:** *LC\_COLLATE=English\_United Kingdom.1252*, *LC\_CTYPE=English\_United Kingdom.1252*, *LC\_MONETARY=English\_United Kingdom.1252*, *LC\_NUMERIC=C* and *LC\_TIME=English\_United Kingdom.1252*

**attached base packages:** *grid*, *stats*, *graphics*, *grDevices*, *utils*, *datasets*, *methods* and *base*

**other attached packages:** *naturalsort(v.0.1.3)*, *broom.mixed(v.0.2.7)*, *MASS(v.7.3-54)*, *lmerTest(v.3.1-3)*, *lme4(v.1.1-27.1)*, *Matrix(v.1.3-3)*, *tidyr(v.1.1.3)*, *dplyr(v.1.0.7)*, *data.table(v.1.14.0)*, *magrittr(v.2.0.1)*, *pca3d(v.0.10.2)*, *ggbiplot(v.0.55)*, *scales(v.1.1.1)*, *plyr(v.1.8.6)* and *ggplot2(v.3.3.5)*

**loaded via a namespace (and not attached):** *tidyselect(v.1.1.1)*, *xfun(v.0.29)*, *bslib(v.0.3.1)*, *pander(v.0.6.4)*, *purrr(v.0.3.4)*, *splines(v.4.1.0)*, *lattice(v.0.20-44)*, *colorspace(v.2.0-2)*, *vctrs(v.0.3.8)*, *generics(v.0.1.2)*, *htmltools(v.0.5.2)*, *yaml(v.2.2.1)*, *utf8(v.1.2.2)*, *rlang(v.0.4.11)*, *nloptr(v.1.2.2.2)*, *jquerylib(v.0.1.4)*, *pillar(v.1.6.2)*, *glue(v.1.4.2)*, *withr(v.2.4.2)*, *DBI(v.1.1.2)*, *lifecycle(v.1.0.0)*, *stringr(v.1.4.0)*, *munsell(v.0.5.0)*, *gtable(v.0.3.0)*, *htmlwidgets(v.1.5.3)*, *evaluate(v.0.14)*, *labeling(v.0.4.2)*, *knitr(v.1.33)*, *fastmap(v.1.1.0)*, *fansi(v.0.5.0)*, *highr(v.0.9)*, *broom(v.0.7.9)*, *Rcpp(v.1.0.7)*, *backports(v.1.2.1)*, *jsonlite(v.1.7.2)*, *farver(v.2.1.0)*, *ellipse(v.0.4.2)*, *digest(v.0.6.27)*, *stringi(v.1.7.3)*, *numDeriv(v.2016.8-1.1)*, *tools(v.4.1.0)*, *rgl(v.0.107.14)*, *sass(v.0.4.0)*, *tibble(v.3.1.3)*, *crayon(v.1.4.1)*, *pkgconfig(v.2.0.3)*, *ellipsis(v.0.3.2)*, *minqa(v.1.2.4)*, *assertthat(v.0.2.1)*, *rmarkdown(v.2.11)*, *R6(v.2.5.1)*, *boot(v.1.3-28)*, *nlme(v.3.1-152)* and *compiler(v.4.1.0)*
