# Supplementary material for: Optimal experimental design for efficient toxicity testing in microphysiological systems: A bone marrow application
Source: Front Pharmacol. 2023 Mar 31;14:1142581. doi: 10.3389/fphar.2023.1142581 (PMC10103791; doi:10.3389/fphar.2023.1142581)
Supplement: Supplementary file 2 [file DataSheet1.zip › Data package/Reports/mixedModel_plot_PAPER_BM-3.html]

Bone Marrow MPS - compare 2018-04/05


Code 

- Show All Code
- Hide All Code

# Bone Marrow MPS - compare 2018-04/05

#### Statistician: Jonathan Cairns

#### 5 April 2019

Read in data:

```
files <- dir(pattern = "output_BM-3")
x <- lapply(files, fread)

files <- gsub("output_BM-3_", "", files, fixed = TRUE)
files <- gsub(".csv", "", files, fixed = TRUE)
names(x) <- files
```

Assemble output

```
output <- NULL

for (i in seq_along(x)) {
  temp <- x[[i]]
  temp <- temp[is.na(temp$group), ]
  temp <- temp[grepl(pattern = ":", temp$term), ]

  extra_info <- as.data.table(do.call(rbind, strsplit(temp$term, ":")))
  colnames(extra_info) <- c("Dose", "Day")
  extra_info$Dose <- gsub("factor(Dose)", "", extra_info$Dose, fixed = TRUE)
  extra_info$Day <- gsub("Day", "", extra_info$Day)
  temp <- cbind(temp, extra_info)
  temp$minus_log10_p <- -log10(temp$p.value)
  temp$model <- files[i]

  output <- rbind(output, temp, fill = TRUE)
}

output$Day <- naturalfactor(output$Day)
```

plot heatmaps

Everything (supplementary)

```
p <- ggplot(aes(x = Day, y = Dose, fill = minus_log10_p), data = output) +
  geom_tile(colour = "black") +
  # geom_label(aes(label = stars), color = "black", fill = "white") +
  # geom_text(aes(label = stars), color = ifelse(output$p.value < 0.005, "black", "white")) +
  geom_text(aes(label = stars), color = "black", size = 7) +
  theme_bw() +
  scale_fill_gradient2(low = "blue", mid = "yellow", high = "red", midpoint = 15) +
  facet_grid(model ~ param) #+

print(p)
```

Model comparison

```
p <- ggplot(aes(x = Day, y = Dose, fill = minus_log10_p), data = output[param == "LateErythroid", ]) +
  geom_tile(colour = "black") +
  # geom_label(aes(label = stars), color = "black", fill = "white") +
  # geom_text(aes(label = stars), color = ifelse(output$p.value < 0.005, "black", "white")) +
  geom_text(aes(label = stars), color = "black", size = 8) +
  theme_bw() +
  scale_fill_gradient2(low = "blue", mid = "yellow", high = "red", midpoint = 15) +
  facet_grid(. ~ model) #+

print(p)
```

Figure 6I - rename “flush” to “operator”.

```
my_data <- output[param == "LateErythroid", ]
my_data$model[my_data$model == "flush"] <- "operator"

p <- ggplot(aes(x = Day, y = Dose, fill = minus_log10_p), data = my_data) +
  geom_tile(colour = "black") +
  # geom_label(aes(label = stars), color = "black", fill = "white") +
  # geom_text(aes(label = stars), color = ifelse(output$p.value < 0.005, "black", "white")) +
  geom_text(aes(label = stars), color = "black", size = 8) +
  theme_bw() +
  scale_fill_gradient2(low = "blue", mid = "yellow", high = "red", midpoint = 15) +
  facet_grid(. ~ model) +
  theme(
      plot.title = element_text(size=20),
      legend.title = element_text(size=20),
      legend.text = element_text(size=20),
      legend.key.width = unit(1,"cm"),
      legend.key.height = unit(1,"cm"),
      axis.text=element_text(size=20),
      axis.title=element_text(size=20),
      strip.text.x=element_text(size=20)
  )

print(p)
```

```
p <- ggplot(aes(x = Day, y = Dose, fill = estimate), data = output) +
  geom_tile(colour = "black") +
  geom_text(aes(label = stars)) +
  theme_bw() +
  scale_fill_gradient(low = "dark blue", high = "yellow") +
  facet_grid(model ~ param) #+

print(p)
```

```
p <- ggplot(aes(x = Day, y = Dose, fill = minus_log10_p), data = output) +
  geom_tile(colour = "black") +
  geom_text(aes(label = signif(p.value, 3))) +
  theme_bw() +
  scale_fill_gradient2(low = "white", mid = "yellow", high = "red", midpoint = 15) +
  facet_grid(model ~ param) #+

print(p)
```

```
pander::pander(sessionInfo())
```

**R version 4.2.1 (2022-06-23 ucrt)**

**Platform:** x86\_64-w64-mingw32/x64 (64-bit)

**locale:** *LC\_COLLATE=English\_United
Kingdom.utf8*, *LC\_CTYPE=English\_United Kingdom.utf8*,
*LC\_MONETARY=English\_United Kingdom.utf8*, *LC\_NUMERIC=C*
and *LC\_TIME=English\_United Kingdom.utf8*

**attached base packages:** *grid*,
*stats*, *graphics*, *grDevices*, *utils*,
*datasets*, *methods* and *base*

**other attached packages:**
*naturalsort(v.0.1.3)*, *broom.mixed(v.0.2.9.4)*,
*MASS(v.7.3-57)*, *lmerTest(v.3.1-3)*,
*lme4(v.1.1-30)*, *Matrix(v.1.5-1)*,
*tidyr(v.1.2.1)*, *dplyr(v.1.0.10)*,
*data.table(v.1.14.2)*, *magrittr(v.2.0.3)*,
*ggbiplot(v.0.55)*, *scales(v.1.2.1)*,
*plyr(v.1.8.7)* and *ggplot2(v.3.4.0)*

**loaded via a namespace (and not attached):**
*Rcpp(v.1.0.9)*, *lattice(v.0.20-45)*,
*listenv(v.0.9.0)*, *assertthat(v.0.2.1)*,
*digest(v.0.6.29)*, *utf8(v.1.2.2)*,
*parallelly(v.1.34.0)*, *R6(v.2.5.1)*,
*backports(v.1.4.1)*, *evaluate(v.0.17)*,
*highr(v.0.9)*, *pillar(v.1.8.1)*,
*rlang(v.1.0.6)*, *rstudioapi(v.0.14)*,
*minqa(v.1.2.4)*, *furrr(v.0.3.1)*,
*jquerylib(v.0.1.4)*, *nloptr(v.2.0.3)*,
*rmarkdown(v.2.17)*, *labeling(v.0.4.2)*,
*splines(v.4.2.1)*, *pander(v.0.6.5)*,
*stringr(v.1.4.1)*, *munsell(v.0.5.0)*,
*broom(v.1.0.1)*, *compiler(v.4.2.1)*,
*numDeriv(v.2016.8-1.1)*, *xfun(v.0.31)*,
*pkgconfig(v.2.0.3)*, *globals(v.0.16.2)*,
*htmltools(v.0.5.3)*, *tidyselect(v.1.2.0)*,
*tibble(v.3.1.8)*, *codetools(v.0.2-18)*,
*fansi(v.1.0.3)*, *future(v.1.31.0)*,
*withr(v.2.5.0)*, *nlme(v.3.1-157)*,
*jsonlite(v.1.8.2)*, *gtable(v.0.3.1)*,
*lifecycle(v.1.0.3)*, *DBI(v.1.1.3)*,
*cli(v.3.4.1)*, *stringi(v.1.7.8)*,
*cachem(v.1.0.6)*, *farver(v.2.1.1)*,
*bslib(v.0.4.0)*, *generics(v.0.1.3)*,
*vctrs(v.0.5.1)*, *boot(v.1.3-28)*,
*tools(v.4.2.1)*, *forcats(v.0.5.2)*,
*glue(v.1.6.2)*, *purrr(v.0.3.5)*,
*parallel(v.4.2.1)*, *fastmap(v.1.1.0)*,
*yaml(v.2.3.5)*, *colorspace(v.2.0-3)*,
*knitr(v.1.40)* and *sass(v.0.4.2)*
